# Supplementary material for: Severe mental illness and health service utilisation for nonpsychiatric medical disorders: A systematic review and meta-analysis
Source: PLoS Med. 2020 Sep 14;17(9):e1003284. doi: 10.1371/journal.pmed.1003284 (PMC7489517; doi:10.1371/journal.pmed.1003284)
Supplement: S2 Appendix — (DOCX) [file pmed.1003284.s002.docx]

**The impact of comorbid severe mental illness on non-psychiatric health service utilisation: A systematic review and meta-analysis**

**Appendix 1: Search Strategy**

**PubMed (All Field Vocabulary)**

On 2018 October 26 (repeated February 2020)

| # | Searches | Results |
| --- | --- | --- |
| 1 | (Medical inpatient [All Fields] OR medical outpatient [All Fields] OR surgical inpatient [All Fields] OR surgical outpatient [All Fields] OR “emergency department [All Fields] OR primary care [All Fields] OR physical illness [All Fields] OR somatic illness [All Fields]) | **743370** |
| 2 | (Comorbidity [All Fields] OR comorbid*[All Fields]) | **190619** |
| 3 | (Severe mental illness [All Fields] OR serious mental illness [All Fields] OR SMI [All Fields] OR schizophrenia [All Fields] OR bipolar disorder [All Fields] OR personality disorder [All Fields]) | **356034** |
| 4 | (Health care use [All Fields] OR health service use [All Fields] OR health care utilisation [All Fields] OR health care utili* [All Fields] OR health service utili* [All Fields] Or length of stay [All Fields]) | **3262153** |
| 5 | #1 AND #2 AND #3 AND #4 | **2508** |
| Additional filters | ‘English language only’ | **1788** |

**PubMed (Controlled Vocabulary ‘MeSH terms’)**

On 2018 November 1 (repeated February 2020)

| # | Searches | Results |
| --- | --- | --- |
| 1 | (Inpatient [MeSH Terms] OR Hospital outpatient clinic [MeSH Terms] OR Outpatient clinic [MeSH Terms] OR Outpatient [MeSH Terms] OR Outpatient health service [MeSH Terms] OR Emergency care [MeSH Terms] OR Primary care [MeSH Terms] OR Chronic illness [MeSH Terms] OR Critical illness [MeSH Terms]) | **725158** |
| 2 | Comorbidities [MeSH Terms] | **96018** |
| 3 | (Schizophrenia and disorders with psychotic features [MeSH Terms] OR Psychosis [MeSH Terms] OR Bipolar disorder [MeSH Terms] OR Borderline personality disorder [MeSH Terms] OR Antisocial personality disorder [MeSH Terms] OR Avoidant personality disorder [MeSH Terms] OR Compulsive personality disorder [MeSH Terms] OR Dependent personality disorder [MeSH Terms] OR Histrionic personality disorder [MeSH Terms] OR Narcissistic personality disorder [MeSH Terms] OR Paranoid personality disorder [MeSH Terms]) | **199790** |
| 4 | (Length of stay [MeSH Terms] OR Medical care costs [MeSH Terms] OR Hospital readmission [MeSH Terms] OR Patient readmission [MeSH Terms] OR Consultations [MeSH Terms]) | **209308** |
| 5 | #1 And #2 AND #3 AND #4 | **109** |
| Additional filters | ‘English language only’ | **97** |

**PsycInfo (All Field Vocabulary)**

On 2018 October 26 (repeated February 2020)

| # | Searches | Results |
| --- | --- | --- |
| 1 | (TX Medical inpatient OR TX Medical outpatient OR TX Surgical inpatient  OR TX Surgical outpatient OR TX Emergency department OR TX Primary care OR TX Physical illness OR TX Somatic illness) | 141829 |
| 2 | (TX Comorbidity OR TX Comorbid*) | **71416** |
| 3 | (TX Severe mental illness OR TX Serious mental illness OR TX SMI OR TX Schizophrenia OR TX Psychosis OR TX Bipolar disorder OR TX Personality disorder) | 247045 |
| 4 | (TX Health care use OR TX Health service use OR TX Health care utilisation OR TX Health care utili* OR TX Health service utili* OR TX Length of stay) | **36070** |
| 5 | #1 And #2 AND #3 AND #4 | **121** |
| Additional filters | ‘English language only’ | **119** |

**PsycInfo (Controlled Vocabulary ‘Subject heading search’)**

On 2018 November 1 (repeated February 2020)

| # | Searches | Results |
| --- | --- | --- |
| 1 | (SU inpatients or hospitalization or 'hospitalized patients' OR SU outpatient clinics or ambulatory care or outpatient services or outpatient care OR SU emergency department or emergency room OR SU primary care OR SU physical illness or chronic illness or chronic disease OR SU critical ill patient or critical illness or critical patient) | 119877 |
| 2 | SU comorbidity or comorbidities or coocurence or comorbid | **48399** |
| 3 | (SU serious mental illness or severe mental illness OR SU SMI OR SU schizophrenia or psychosis or psychoses or psychotic disorder or schizophrenic disorder Or SU bipolar disorder OR SU Personality disorder) | 198033 |
| 4 | (SU Health service use OR SU health service utilization or utilisation OR SU length of stay or hospitalization or length in hospital OR SU length of stay or LOS or inpatient stay or time in hospital or time to discharge) | **53380** |
| 5 | #1 And #2 And #3 AND #4 | **638** |
| Additional filters | ‘English language only’ | **607** |

**Web of Science (All Field Vocabulary ‘Topic’)**

On 2018 November 2 (repeated February 2020)

| # | Searches | Results |
| --- | --- | --- |
| 1 | Topic: (Medical inpatient OR Medical outpatient OR Surgical inpatient OR Surgical outpatient OR Emergency department OR Primary care OR Physical illness OR Somatic illness) | **361581** |
| 2 | Topic: (Comorbidity OR Comorbid*) | **145481** |
| 3 | Topic: (Severe mental illness OR Serious mental illness OR SMI OR Schizophrenia OR Psychosis OR Bipolar disorder OR Personality disorder) | **272971** |
| 4 | Topic: (Health care use OR Health service use OR Health care utilisation OR Health care utili* OR Health service utili* OR Length of stay) | **433215** |
| 5 | #1 And #2 And #3 AND #4 | **845** |
| Additional filters | ‘English language only’ | **818** |

**Web of Science Core Collection employs no controlled vocabulary**

**Cochrane Library (All Field Vocabulary ‘Title, Abstract, Keyword’)**

On 2018 November 2 (repeated February 2020)

| # | Searches | Results |
| --- | --- | --- |
| 1 | ((Medical inpatient):ti,ab,kw OR (Medical outpatient):ti,ab,kw OR (Surgical inpatient):ti,ab,kw OR (Surgical outpatient):ti,ab,kw OR ("emergency department"):ti,ab,kw OR (primary care):ti,ab,kw OR (physical illness):ti,ab,kw OR (somatic illness):ti,ab,kw) | **71838** |
| 2 | ((Comorbidity):ti,ab,kw OR (Comorbid*):ti,ab,kw) | **17024** |
| 3 | ((Severe mental illness):ti,ab,kw OR (Serious mental illness):ti,ab,kw OR (SMI):ti,ab,kw OR (Schizophrenia):ti,ab,kw OR (Psychosis):ti,ab,kw OR (Bipolar disorder):ti,ab,kw OR (Personality disorder):ti,ab,kw) | **31039** |
| 4 | ((Health care use):ti,ab,kw OR (Health service use):ti,ab,kw OR (Health care utilisation):ti,ab,kw OR (Health care utili*):ti,ab,kw OR (Health service utili*):ti,ab,kw OR (Length of stay):ti,ab,kw) | **72019** |
| 5 | #1 AND #2 AND #3 AND #4 | **258** |
| Additional filters | Removed one editorial | **257** |

**Cochrane Library (Controlled Vocabulary)**

On 2018 November 2 (repeated February 2020)

| # | Searches | Results |
| --- | --- | --- |
| 1 | (MeSH descriptor: [Inpatients] explode all trees OR MeSH descriptor: [Outpatients] explode all trees OR MeSH descriptor: [Emergency Service, Hospital] explode all trees OR MeSH descriptor: [Primary Health Care] explode all trees OR MeSH descriptor: [Chronic Disease] explode all trees OR MeSH descriptor: [Critical Illness] explode all trees) | **17997** |
| 2 | (MeSH descriptor: [Comorbidity] explode all trees) | **3393** |
| 3 | (MeSH descriptor: [Bipolar and Related Disorders] explode all trees OR MeSH descriptor: [Personality Disorders] explode all trees OR MeSH descriptor: [Schizophrenia Spectrum and Other Psychotic Disorders] explode all trees OR MeSH descriptor: [Schizophrenia] explode all trees OR MeSH descriptor: [Psychotic Disorders] explode all trees) | **10999** |
| 4 | (MeSH descriptor: [Length of Stay] explode all trees OR MeSH descriptor: [Patient Readmission] explode all trees OR MeSH descriptor: [Health Care Costs] explode all trees OR MeSH descriptor: [Health Services] explode all trees) | **87698** |
| 5 | #1 And #2 AND #3 AND #4 | **11** |

**EMBASE (All fields)**

On 2018 November 2 (repeated February 2020)

| # | Searches | Results |
| --- | --- | --- |
| 1 | "Hospital patient" [all fields] OR "Outpatient" [all fields] OR "Emergency ward" [all fields] OR "Primary health care" [all fields] OR "Physical disease" [all fields] OR "Chronic disease" [all fields] | **836571** |
| 2 | Comorbidity [all fields] | **247551** |
| 3 | "Severe mental illness" [candidate term] OR "Serious mental illness" [candidate term] OR Schizophrenia [all fields] OR Psychosis [all fields]  "Bipolar disorder" [all fields] OR "Personality disorder" [all fields] | **369800** |
| 4 | "Health care utilization" [all fields] OR "Length of stay" [all fields] | **227679** |
| 5 | #1 And #2 And #3 AND #4 | **285** |

**EMBASE (Controlled vocabulary; Emtree explosion)**

On 2018 November 2 (repeated February 2020)

| # | Searches | Results |
| --- | --- | --- |
| 1 | (hospital patient'/exp OR 'outpatient'/exp OR 'emergency health service'/exp OR 'primary health care'/exp OR 'general practitioner'/exp OR 'physical disease'/exp) | **20989997** |
| 2 | ('comorbidity'/exp) | **217887** |
| 3 | ('psychosis'/exp OR 'schizophrenia'/exp OR 'bipolar disorder'/exp OR 'personality disorder'/exp) | **384271** |
| 4 | ('health care utilization'/exp OR 'length of stay'/exp OR 'hospital readmission'/exp) | **246333** |
| 5 | #1 AND #2 AND #3 AND#4 | **638** |
