# Supplementary material for: Severe mental illness and health service utilisation for nonpsychiatric medical disorders: A systematic review and meta-analysis
Source: PLoS Med. 2020 Sep 14;17(9):e1003284. doi: 10.1371/journal.pmed.1003284 (PMC7489517; doi:10.1371/journal.pmed.1003284)
Supplement: S3 Appendix — (DOCX) [file pmed.1003284.s003.docx]

**The impact of comorbid severe mental illness on non-psychiatric health service utilisation: A systematic review and meta-analysis**

**Appendix 2: List of excluded studies**

| **Authors** | **Title** | **Year** | **Journal** | **Reason for exclusion** |
| --- | --- | --- | --- | --- |
| Abrams, T. E.; Vaughan-Sarrazin, M.; Rosenthal, G. E. | Influence of Psychiatric Comorbidity on Surgical Mortality | 2010 | *Archives of Surgery* | Wrong outcome |
| Abrams, T. E.; Vaughan-Sarrazin, M.; Rosenthal, G. E. | Preexisting comorbid psychiatric conditions and mortality in nonsurgical intensive care patients | 2010 | *American Journal of Critical Care* | Wrong outcome |
| Albrecht, J.; Hirshon, J. M.; Goldberg, R.; Day, H. R.; Morgan, D. J.; Comer, A. C.; Harris, A. D.; Furuno, J. P. | Severe mental illness and hospital readmission in older diabetic adults | 2011 | *J Am Geriatr Soc* | Conference abstract |
| Albrecht, J.; Hirshon, J. M.; Goldberg, R.; Langenberg, P.; Day, H.; Morgan, D.; Comer, A.; Harris, A.; Furuno, J. | Severe mental illness and hospital readmission in diabetic adults | 2011 | *Am J Epidemiol* | Conference abstract |
| Albrecht, J.; Hirshon, J. M.; Goldberg, R.; Langenberg, P.; Day, H.; Morgan, D.; Comer, A.; Harris, A.; Furuno, J. | Severe mental illness and acute hospital readmission in diabetic patients | 2012 | *Am J Med Qual* | Incorrect definition of SMI |
| An, J.; Le, Q. A.; Dang, T. | Association between different types of comorbidity and disease burden in patients with diabetes | 2019 | *J Diabetes* | Wrong patient group |
| Annapureddy, N.; Saha, A.; Nadkarni, G. | Hospital readmissions for SLE in the United States: A national database study | 2017 | *Arthritis and Rheumatology* | Conference abstract |
| Anttalainen, U.; Polo, O.; Vahlberg, T.; Saaresranta, T. | Reimbursed drugs in patients with sleep-disordered breathing: A static-charge-sensitive bed study | 2010 | *Sleep Med* | Wrong outcome |
| Arredondo, S.; Dome, M. R.; Gilder, R. E.; Roden-Foreman, J. W.; Foreman, M. L.; Petrey, L. B. | Identifying trauma superutilizers readmissions with 11-year data model | 2017 | *Journal of the American College of Surgeons* | Conference abstract |
| Ashton, C. M.; Septimus, J.; Petersen, N. J.; Souchek, J.; Menke, T. J.; Collins, T. C.; Wray, N. P. | Healthcare use by veterans treated for diabetes mellitus in the Veterans Affairs medical care system | 2003 | *American Journal of Managed Care* | The impact of SMI on HSU was not assessed |
| Bade, B. C.; DeRycke, E. C. ; Ramsey, C.; Skanderson, M.; Crothers, K.; Haskell, S.; Bean-Mayberry, B.; Brandt, C.; Bastian, L. A.; Akgun, K. M. | Sex differences in veterans admitted to the hospital for chronic obstructive pulmonary disease exacerbation | 2019 | *Ann Am Thorac Soc* | The impact of SMI on HSU was not assessed |
| Badheka, A. O.; Patel, N. J.; Panaich, S. S.; Patel, S. V.; Jhamnani, S.; Singh, V.; Pant, S.; Patel, N.; Patel, N.; Arora, S.; Thakkar, B.; Manvar, S.; Dhoble, A.; Patel, A.; Savani, C.; Patel, J.; Chothani, A.; Savani, G. T.; Deshmukh, A.; Grines, C. L.; Curtis, J.; Mangi, A. A.; Cleman, M.; Forrest, J. K. | Effect of hospital volume on outcomes of transcatheter aortic valve implantation | 2015 | *American Journal of Cardiology* | The impact of SMI on HSU was not assessed |
| Bartels, S. J.; Pratt, S. I.; Mueser, K. T.; Naslund, J. A.; Wolfe, R. S.; Santos, M.; Xie, H.; Riera, E. G. | Integrated IMR for psychiatric and general medical illness for adults aged 50 or older with serious mental illness | 2014 | *Psychiatr Serv* | Wrong patient group |
| Battersby, M.; Kidd, M. R.; Licinio, J.; Aylward, P.; Baker, A.; Ratcliffe, J.; Quinn, S.; Castle, D. J.; Zabeen, S.; Fairweather-Schmidt, A. K.; et al., | Improving cardiovascular health and quality of life in people with severe mental illness: study protocol for a randomised controlled trial | 2018 | *Trials* | Study protocol |
| Baumeister, H.; Haschke, A.; Munzinger, M.; Hutter, N.; Tully, P. J. | Inpatient and outpatient costs in patients with coronary artery disease and mental disorders: a systematic review | 2015 | *Biopsychosocial Medicine* | Systematic review |
| Becker, C.; Brobert, G. P.; Almqvist, P. M.; Johansson, S.; Jick, S. S.; Meier, C. R. | Migraine incidence, comorbidity and health resource utilization in the UK | 2008 | *Cephalalgia* | The impact of SMI on HSU was not assessed |
| Bera, R.; Karson, C.; Offord, S.; Zubek, D.; Lau, G.; Lin, J. | Comparison of healthcare resource usage and costs before and after initiating long-AC ting injectable antipsychotics among medicaid insured patients with schizophrenia | 2013 | *Schizophr Bull* | Conference abstract |
| Bhandari, S.; Venkatesan, T. | Clinical Characteristics, Comorbidities and Hospital Outcomes in Hospitalizations with Cyclic Vomiting Syndrome: A Nationwide Analysis | 2017 | *Digestive Diseases and Sciences* | The impact of SMI on HSU was not assessed |
| Boaz, T. L.; Becker, M. A.; Andel, R.; McCutchan, N. | Rehospitalization risk factors for psychiatric treatment among elderly Medicaid beneficiaries following hospitalization for a physical health condition | 2017 | *Aging Ment Health* | Wrong outcome |
| Bouza, C.; Lopez-Cuadrado, T.; Amate, J. M. | Hospital admissions due to physical disease in people with schizophrenia: a national population-based study | 2010 | *Gen Hosp Psychiatry* | Wrong patient group |
| Bresee, L. C.; Majumdar, S. R.; Patten, S. B.; Johnson, J. A. | Diabetes, cardiovascular disease, and health care use in people with and without schizophrenia | 2011 | *European Psychiatry* | Wrong patient group |
| Breslau, J.; Leckman-Westin, E.; Han, B.; Pritam, R.; Guarasi, D.; Horvitz-Lennon, M.; Scharf, D. M.; Finnerty, M. T.; Yu, H. | Impact of a mental health based primary care program on emergency department visits and inpatient stays | 2018 | *Gen Hosp Psychiatry* | Wrong patient group |
| Brink, M.; Green, A.; Bojesen, A. B.; Lamberti, J. S.; Conwell, Y.; Andersen, K. | Physical Health, Medication, and Healthcare Utilization among 70-Year-Old People with Schizophrenia: A Nationwide Danish Register Study | 2017 | *American Journal of Geriatric Psychiatry* | Wrong patient group |
| Brostedt, E. M.; Msghina, M.; Persson, M.; Wettermark, B. | Health care use, drug treatment and comorbidity in patients with schizophrenia or non-affective psychosis in Sweden: A cross-sectional study | 2017 | *BMC Psychiatry* | Wrong patient group |
| Butler, H.; O'Brien, A. J. | Access to specialist palliative care services by people with severe and persistent mental illness: A retrospective cohort study | 2018 | *Int J Ment Health Nurs* | Incorrect definition of SMI |
| Cai, Xueya; Li, Yue | Are AMI Patients with Comorbid Mental Illness More Likely to be Admitted to Hospitals with Lower Quality of AMI Care? | 2013 | *PLoS One* | The impact of SMI on HSU was not assessed |
| Cailhol, L.; Francois, M.; Thalamas, C.; Garrido, C.; Birmes, P.; Pourcel, L.; Lapeyre-Mestre, M.; Paris, J. | Is borderline personality disorder only a mental health problem? | 2016 | *Personal Ment Health* | Wrong outcome |
| Calderón-Larranaga, A.; Abad-DÃ­ez, J. M.; Gimeno-Feliu, L. A.; Marta-Moreno, J.; GonzÃ¡lez-Rubio, F.; Clerencia-Sierra, M.; Poblador-Plou, B.; Poncel-FalcÃ³, A.; Prados-Torres, A. | Global health care use by patients with type-2 diabetes: Does the type of comorbidity matter? | 2015 | *European Journal of Internal Medicine* | The impact of SMI on HSU was not assessed |
| Carter, P.; Carter, A.; Reynolds, J.; Uppal, H.; Chandran, S.; Potluri, R. | The impact of psychiatric comorbidities on the length of hospital stay in patients with heart failure | 2016 | *Heart* | Conference abstract |
| Carter, P. R.; Reynolds, J.; Carter, A. J.; Uppal, H.; Chandran, S.; Potluri, R. | The impact of psychiatric co-morbidities on the length of stay in patients with heart failure | 2016 | *European Heart Journal* | Conference abstract |
| Cavanaugh, PK; Chen, AF; Rasouli, MR; et al. | Complications and Mortality in Chronic Renal Failure Patients Undergoing Total Joint Arthroplasty: A Comparison Between Dialysis and Renal Transplant Patients. | 2016 | *Journal of Arthroplasty* | Reported incorrect results |
| Cealicu Toma, F.; MoamaÃ¯, J. | Multimorbidity in affective disorders: Impact on length of stay | 2016 | *European Psychiatry* | Conference abstract |
| Centorrino, F.; Mark, T. L.; Talamo, A.; Oh, K.; Chang, J. | Health and Economic Burden of Metabolic Comorbidity Among Individuals With Bipolar Disorder | 2009 | *J Clin Psychopharmacol* | Wrong patient group |
| Chang, H. J.; Liao, C. C.; Hu, C. J.; Shen, W. W.; Chen, T. L. | Psychiatric Disorders after Epilepsy Diagnosis: A Population-Based Retrospective Cohort Study | 2013 | *PLoS One* | The impact of SMI on HSU was not assessed |
| Chopra, T.; Marchaim, D.; Lynch, Y.; Kosmidis, C.; Zhao, J. J.; Dhar, S.; Gheyara, N.; Turner, D.; Gulish, D.; Wood, M.; Alangaden, G.; Kaye, K. S. | Epidemiology and outcomes associated with surgical site infection following bariatric surgery | 2012 | *Am J Infect Control* | The impact of SMI on HSU was not assessed |
| Chwastiak, L. A.; Jackson, S. L.; Russo, J.; DeKeyser, P.; Kiefer, M.; Belyeu, B.; Mertens, K.; Chew, L.; Lin, E. | A collaborative care team to integrate behavioral health care and treatment of poorly-controlled type 2 diabetes in an urban safety net primary care clinic | 2017 | *Gen Hosp Psychiatry* | The impact of SMI on HSU was not assessed |
| Cooper, M. N.; Lin, A.; Alvares, G. A.; de Klerk, N. H.; Jones, T. W.; Davis, E. A. | Psychiatric disorders during early adulthood in those with childhood onset type 1 diabetes: Rates and clinical risk factors from population-based follow-up | 2017 | *Pediatric Diabetes* | The impact of SMI on HSU was not assessed |
| Crits-Christoph, P.; Gallop, R.; Noll, E.; Rothbard, A.; Diehl, C. K.; Gibbons, M. B. C.; Gross, R.; Rhodes, K. V. | Impact of a medical home model on costs and utilization among comorbid HIV-positive medicaid patients | 2018 | *American Journal of Managed Care* | The impact of SMI on HSU was not assessed |
| Daumit, G. L.; McGinty, E. E.; Pronovost, P.; Dixon, L. B.; Guallar, E.; Ford, D. E.; Cahoon, E. K.; Boonyasai, R. T.; Thompson, D. | Patient safety events and harms during medical and surgical hospitalizations for persons with serious mental illness | 2016 | *Psychiatric Services* | Wrong patient group |
| DeStefano, L.; Fults, M.; Rao, S.; Matthews, J.; Malhan, S.; Schwartz, K.; Kish, K. | The influence of psychiatric health on breast abscesses in the non-lactating patient: A community hospital's experience | 2017 | *Annals of Surgical Oncology* | Conference abstract |
| Durbin, A.; Brown, H. K.; Bansal, S.; Antoniou, T.; Jung, J. K. H.; Lunsky, Y. | How HIV affects health and service use for adults with intellectual and developmental disabilities | 2017 | *Journal of intellectual disability research* | The impact of SMI on HSU was not assessed |
| Ebuenyi, I.; Taylor, C.; Oâ€™Flynn, D.; Matthew Prina, A.; Passchier, R.; Mayston, R. | The Impact of co-morbid severe mental illness and HIV upon mental and physical health and social outcomes: a systematic review | 2018 | *AIDS Care - Psychological and Socio-Medical Aspects of AIDS/HIV* | Systematic review |
| Fine, M. J.; Smith, D. N.; Singer, D. E. | Hospitalization decision in patients with community-acquired pneumonia: a prospective cohort study | 1990 | *Am J Med* | The impact of SMI on HSU was not assessed |
| Fineberg, S. J.; Nandyala, S. V.; Marquez-Lara, A.; Oglesby, M. W.; Pelton, M. A.; Patel, A. A.; Singh, K. | Risk factors for urinary complications after cervical spine surgery | 2013 | *Spine Journal* | Wrong outcome |
| Furlanetto, L. M.; Da Silva, R. V.; Bueno, J. R. | The impact of psychiatric comorbidity on length of stay of medical inpatients | 2003 | *Gen Hosp Psychiatry* | The impact of SMI on HSU was not assessed |
| Germack, H. D.; Noor, E. Alam M.; Wang, X.; Hanrahan, N. | Association of Comorbid Serious Mental Illness Diagnosis with 30-Day Medical and Surgical Readmissions | 2019 | *JAMA Psychiatry* | Incorrect definition of SMI |
| Gervaix, J.; Haour, G.; Michel, M.; Chevreul, K. | Impact of mental illness on care for somatic comorbidities in France: a nation-wide hospital-based observational study | 2019 | *Epidemiol Psychiatr Sci* | The impact of SMI on HSU was not assessed |
| Goulet, J. L.; Kerns, R. D.; Bair, M.; Becker, W. C.; Brennan, P.; Burgess, D. J.; Carroll, C. M.; Dobscha, S.; Driscoll, M. A.; Fenton, B. T.; Fraenkel, L.; Haskell, S. G.; Heapy, A. A.; Higgins, D. M.; Hoff, R. A.; Hwang, U.; Justice, A. C.; Piette, J. D.; Sinnott, P.; Wandner, L.; Womack, J. A.; Brandt, C. A. | The musculoskeletal diagnosis cohort: examining pain and pain care among veterans | 2016 | *Pain* | The impact of SMI on HSU was not assessed |
| Greenberg, J. K.; Ladner, T. R.; Olsen, M. A.; Shannon, C. N.; Liu, J.; Yarbrough, C. K.; Piccirillo, J. F.; Wellons, J. C.; Smyth, M. D.; Park, T. S.; Limbrick, D. D. | Complications and Resource Use Associated with Surgery for Chiari Malformation Type 1 in Adults: A Population Perspective | 2015 | *Neurosurgery* | The impact of SMI on HSU was not assessed |
| Greenwood, K. L.; LaMori, J. C.; Smith, B.; Doshi, D.; Davis, C. | Impact of Behavioral Health Screening on Proactive Identification of Patients at Risk for Hospital Readmission | 2019 | *Popul Health Manag* | Incorrect definition of SMI |
| Gunzler, D. D.; Morris, N.; Dalton, J. E.; McCormick, R.; Dawson, N. V.; Thomas, C.; Kanuch, S.; Cassidy, K. A.; Athey, M.; Fuentes-Casiano, E.; et al., | Clinic Appointment Attendance in Adults with Serious Mental Illness and Diabetes | 2017 | *Am J Health Behav* | The impact of SMI on HSU was not assessed |
| Gupta, N. M.; Yu, P. C.; Imrey, P.; Rothberg, M. B. | Impact of alcohol-related diagnoses on outcomes of patients hospitalized with pneumonia | 2018 | *J Gen Intern Med* | Full text unavailable |
| Han, C. L.; Lofland, J. H.; Zhao, N.; Schenkel, B. | Increased Prevalence of Psychiatric Disorders and Health Care-Associated Costs Among Patients With Moderate-to-Severe Psoriasis | 2011 | *Journal of Drugs in Dermatology* | Full text unavailable |
| Handschin, A. E.; Vetter, S.; Jung, F. J.; Guggenheim, M.; KÃ¼nzi, W.; Giovanoli, P. | A Case-matched controlled study on high-voltage electrical injuries vs thermal burns | 2009 | *Journal of Burn Care and Research* | The impact of SMI on HSU was not assessed |
| Hanrahan, N. P.; Bressi, S.; Marcus, S.C.; Solomon, P. | Examining the impact of comorbid serious mental illness on rehospitalisation among medical and surgical inpatients | 2016 | *General Hospital Psychiatry* | Incorrect definition of SMI |
| Hasan, R.; Nicolaidis, C. | Mental health contributions to costand length of hospitalization in adolescents and young adults with chronic medical conditions | 2018 | *J Gen Intern Med* | Conference abstract |
| Hassan, M.; Rajagopalan, K.; Stafkey-Mailey, D.; Farrelly, E.; Eaddy, M.; Loebel, A. | Impact of metabolic comorbidities on inpatient cost and rehospitalization rates for patients diagnosed with schizophrenia | 2013 | *Value in Health* | Conference abstract |
| Hassan, A.; Wu, S. S.; Schmidt, P.; Dai, Y.; Simuni, T.; Giladi, N.; Bloem, B. R.; Malaty, I. A.; Okun, M. S. | High rates and the risk factors for emergency room visits and hospitalization in Parkinson's disease | 2013 | *Parkinsonism Relat Disord* | The impact of SMI on HSU was not assessed |
| Henriksen, D. P.; Pottegard, A.; Laursen, C. B.; Jensen, T. G.; Hallas, J.; Pedersen, C.; Lassen, A. T. | Risk factors for hospitalization due to community-acquired sepsis - a population-based case-control study | 2015 | *PLoS One* | Incorrect definition of SMI |
| Hepp, Z.; Lage, M. J.; Espaillat, R.; Gossain, V. V. | The association between adherence to levothyroxine and economic and clinical outcomes in patients with hypothyroidism in the US | 2018 | *J Med Econ* | The impact of SMI on HSU was not assessed |
| Himelhoch, S.; Chander, G.; Fleishman, J. A.; Hellinger, J.; Gaist, P.; Gebo, K. A. | Access to HAART and utilisation of inpatient medical hospitals services among HIV-infected patients with co-occurring serious mental illness and injection drug use | 2007 | *Gen Hosp Psychiatry* | Incorrect definition of SMI |
| Ho, W.; Wang, J.; Chien, K. L.; Wu, F. L. L.; Lin, Z. F. | The impact of comorbidity and age on treatments for acute coronary syndrome | 2016 | *Pharmacoepidemiol Drug Saf* | The impact of SMI on HSU was not assessed |
| Hoover, D. R.; Sambamoorthi, U.; Walkup, J. T.; Crystal, S. | Mental illness and length of inpatient stay for Medicaid recipients with AIDS | 2004 | *HSR: Health Services Research* | Incorrect definition of SMI |
| Humphreys, J.; Ahalt, C.; Stijacic-Cenzer, I.; Widera, E.; Williams, B. | Six-Month Emergency Department Use among Older Adults Following Jail Incarceration | 2018 | *Journal of Urban Health-Bulletin of the New York Academy of Medicine* | Incorrect definition of SMI |
| Hustedt, J. W.; Chung, A.; Bohl, D. D.; Olmscheid, N.; Edwards, S. | Evaluating the Effect of Comorbidities on the Success, Risk, and Cost of Digital Replantation | 2016 | *J Hand Surg Am* | Patients under the age of 18 |
| Iglay, K.; Santorelli, M. L.; Hirshfield, K. M.; Williams, J. M.; Rhoads, G. G.; Lin, Y.; Demissie, K. | Impact of Preexisting Mental Illness on All-Cause and Breast Cancer-Specific Mortality in Elderly Patients With Breast Cancer | 2017 | *J Clin Oncol* | Wrong outcome |
| Inneh, I. A.; Lewis, C. G.; Schutzer, S. F. | Focused Risk Analysis: Regression Model Based on 5,314 Total Hip and Knee Arthroplasty Patients from a Single Institution | 2014 | *Journal of Arthroplasty* | Incorrect definition of SMI |
| Ivanova, J. I.; Bergman, R.; Birnbaum, H. G.; Colice, G. L.; Silverman, R. A.; McLaurin, K. | Effect of asthma exacerbations on health care costs among asthmatic patients with moderate and severe persistent asthma | 2012 | *J Allergy Clin Immunol* | The impact of SMI on HSU was not assessed |
| Iyer, U. R.; Merchant, A. M. | Outcomes of Ventral Hernia Repair in Solid Organ Transplant Patients: A Regression Analysis of the National Inpatient Sample | 2019 | *J Surg Res* | The impact of SMI on HSU was not assessed |
| Jackson, C.; DuBard, A.; Swartz, M.; Mahan, A.; McKee, J.; Pikoulas, T.; Moran, K.; Lancaster, M. | Readmission Patterns and Effectiveness of Transitional Care Among Medicaid Patients With Schizophrenia and Medical Comorbidity | 2015 | *N C Med J* | Wrong patient group |
| Jayatilleke, N.; Hayes, R. D.; Chang, C. K.; Stewart, R. | Acute general hospital admissions in people with serious mental illness | 2018 | *Psychol Med* | Wrong patient group |
| Jensen, L. F.; Pedersen, A. F.; Andersen, B.; Vestergaard, M.; Vedsted, P. | Non-participation in breast cancer screening for women with chronic diseases and multimorbidity: a population-based cohort study | 2015 | *BMC Cancer* | Wrong outcome |
| Jette, N.; Patten, S.; Williams, J.; Becker, W.; Wiebe, S. | Comorbidity of migraine and psychiatric disorders - A national population-based study | 2008 | *Headache* | Wrong outcome |
| Jimenez-Almonte, J. H.; Nzegwu, I. N.; Liu, B.; Akhtar, Z.; Grabau, J. D.; Mehdi, S. K.; Jacobs, C.; Cassidy, R. C. | 60. Spine patients demystified: what are the predictive factors of poor surgical outcomes in patients after elective lumbar spine surgery? | 2019 | *The Spine J* | Conference abstract |
| Jorgensen, C. C.; Knop, J.; Nordentoft, M.; Kehlet, H.; Lundbeck, Fdn | Psychiatric Disorders and Psychopharmacologic Treatment as Risk Factors in Elective Fast-track Total Hip and Knee Arthroplasty | 2015 | *Anesthesiology* | Incorrect definition of SMI |
| Kang, J. H.; Xirasagar, S.; Lin, H. C. | Lower mortality among stroke patients with schizophrenia: A nationwide population-based study | 2011 | *Psychosom Med* | Wrong outcome |
| Karunakaran, A.; Zhao, H.; Rubin, D. J. | Predischarge and Postdischarge Risk Factors for Hospital Readmission among Patients with Diabetes | 2018 | *Med Care* | The impact of SMI on HSU was not assessed |
| Khoury, H.; Sanaiha, Y.; Rudasill, S. E.; Mardock, A. L.; Sareh, S.; Benharash, P. | Readmissions Following Isolated Coronary Artery Bypass Graft Surgery in the United States (from the Nationwide Readmissions Database 2010 to 2014) | 2019 | *Am J Cardiol* | The impact of SMI on HSU was not assessed |
| Kim, C. Y.; Sivasundaram, L.; LaBelle, M. W.; Trivedi, N. N.; Liu, R. W.; Gillespie, R. J. | Predicting adverse events, length of stay, and discharge disposition following shoulder arthroplasty: a comparison of the Elixhauser Comorbidity Measure and Charlson Comorbidity Index | 2018 | *Journal of Shoulder and Elbow Surgery* | The impact of SMI on HSU was not assessed |
| Kim, A. M.; Rossi, K. C.; JettÃ©, N.; Yoo, J. Y.; Hung, K.; Dhamoon, M. S. | Increased risk of hospital admission for mood disorders following admission for epilepsy | 2018 | *Neurology* | Wrong outcome |
| King, D. A.; Hussein, E.; Bar-Lavie, Y. | Admission rate and outcome of patients with major psychiatric disorders in the intensive care unit | 2017 | *Am J Respir Crit Care Med* | Conference abstract |
| Kisely, S.; Xiao, J.;Lawrence, D.; Jian, L. | Is the effect of compulsory community treatment on preventable deaths from physical disorders mediated by better access to specialised medical procedures? | 2014 | *Can J Psychiatry* | Wrong patient group |
| Kisely, S.; Ehrlich, C.; Kendall, E.; Lawrence, D. | Using Avoidable Admissions to Measure Quality of Care for Cardiometabolic and Other Physical Comorbidities of Psychiatric Disorders: A Population-Based, Record-Linkage Analysis | 2015 | *Canadian Journal of Psychiatry-Revue Canadienne De Psychiatrie* | The impact of SMI on HSU was not assessed |
| Klein, Colin; Prokhorov, Tatiana; Miniovitz, Ala; Dobronevsky, Eugenia; Rabey, Jose Martin | Admission of Parkinsonian patients to a neurological ward in a community hospital | 2009 | *Journal of Neural Transmission* | Wrong outcome |
| Kunik, Mark E.; Cully, Jeffrey A.; Snow, A. Lynn; Souchek, Julie; Sullivan, Greer; Ashton, Carol M. | Treatable Comorbid Conditions and Use of VA Health Care Services Among Patients With Dementia | 2005 | *Psychiatric Services* | Wrong patient group |
| Kwesiga, E.; Joks, R.; Durkin, H.; Goodman, B. | Association of psychiatric comorbidities with lengths of stay and reimbursement in admitted asthma patients | 2017 | *Annals of Allergy, Asthma and Immunology* | Conference abstract |
| Lapar, D. J.; Bhamidipati, C. M.; Mery, C. M.; Stukenborg, G. J.; Jones, D. R.; Schirmer, B. D.; Kron, I. L.; Ailawadi, G. | Primary payer status affects mortality for major surgical operations | 2010 | *Annals of Surgery* | The impact of SMI on HSU was not assessed |
| Lee, S.; Black, D.; Held, M. | Associations of multiplicity of comorbid health conditions, serious mental illness, and health care costs | 2016 | *Soc Work Health Care* | The impact of SMI on HSU was not assessed |
| Lilly, F. R.; Culpepper, J.; Stuart, M.; Steinwachs, D. | Stroke survivors with severe mental illness: Are they at-risk for increased non-psychiatric hospitalizations? | 2017 | *PLoS One* | Incorrect definition of SMI |
| Louis, E. D.; Henchcliffe, C.; Bateman, B. T.; Schumacher, C. | Young-onset Parkinson's disease: Hospital utilization and medical comorbidity in a nationwide survey | 2007 | *Neuroepidemiology* | The impact of SMI on HSU was not assessed |
| Lunsky, Y.; Lin, E.; Balogh, R.; Klein-Geltink, J.; Wilton, A. S.; Kurdyak, P. | Emergency department visits and use of outpatient physician services by adults with developmental disability and psychiatric disorder | 2012 | *Can J Psychiatry* | Wrong patient group |
| Lunsky, Y.; Durbin, A.; Brown, H. K.; Bansal, S.; Heifetz, M.; Antoniou, T. | Health profiles and associated service use among adults with HIV and intellectual and developmental disabilities | 2017 | *Aids* | The impact of SMI on HSU was not assessed |
| Lyketsos, C. G.; Dunn, G.; Kaminsky, M. J.; Breakey, W. R. | Medical comorbidity in psychiatric inpatients: relation to clinical outcomes and hospital length of stay | 2002 | *Psychosomatics* | Wrong patient group |
| Margolis, J. M.; Masters, E. T.; Cappelleri, J. C.; Smith, D. M.; Faulkner, S. | Evaluating increased resource use in fibromyalgia using electronic health records | 2016 | *Clinicoeconomics and Outcomes Research* | Incorrect definition of SMI |
| McIntyre, R. S.; Konarski, J. Z.; Wilkins, K.; Bouffard, B.; Soczynska, J. K.; Kennedy, S. H. | The prevalence and impact of migraine headache in bipolar disorder: Results from the Canadian Community Health Survey | 2006 | *Headache* | Wrong patient group |
| McPherson, S.; Barbosa-Leiker, C.; Daratha, K.; Short, R.; McDonell, M. G.; Alicic, R.; Roll, J.; Tuttle, K. | Association of co-occurring serious mental illness with emergency hospitalization in people with chronic kidney disease | 2014 | *Am J Nephrol* | Incorrect definition of SMI |
| Meyer, J. P.; Qiu, J. J.; Chen, N. E.; Larkin, G. L.; Altice, F. L. | Emergency Department Use by Released Prisoners with HIV: An Observational Longitudinal Study | 2012 | *PLoS One* | The impact of SMI on HSU was not assessed |
| Minen, M.; Tanev, K. | Influence of psychiatric comorbidities in migraineurs in the emergency department: A cross sectional analysis | 2014 | *Neurology* | Conference abstract |
| Nagaraja, V.; Cohen, M. G.; Suh, W.; Alasnag, M.; Genereux, P.; Potts, J.; Gunning, M.; Nolan, J.; Bagur, R.; Mamas, M. A. | Non-Cardiovascular Comorbidities as Evaluated by Elixhauser Comorbidity Score in Individuals Undergoing TAVR | 2019 | *J Heart Team* | The impact of SMI on HSU was not assessed |
| Nasri, B.; Tada, M.; Yuu, K.; Calin, M. | Laparoscopic versus open surgery in patients with psychiatric comorbidity: A retrospective study at the largest psychiatric center in Japan | 2016 | *Surgical Endoscopy and Other Interventional Techniques* | Conference abstract |
| Nasri, B.; Yuu, K.; Tada, M.; Nishi, N.; Calin, M. | Short term outcomes of laparoscopic colorectal resection in psychiatric population: A multicenters retrospective study in Japan | 2017 | *Surgical Endoscopy and Other Interventional Techniques* | Conference abstract |
| Nath, Sara Bressi; Marcus, Steven C. | Impact of psychotic disorders on discharge dispositions of adults 65 or older after a general medical inpatient stay | 2012 | *Psychiatric Services* | Wrong outcome |
| Navon, L. | Hospitalization Trends and Comorbidities Among People With HIV/AIDS Compared With the Overall Hospitalized Population, Illinois, 2008-2014 | 2018 | *Public Health Reports* | Wrong outcome |
| Nepple, K.; Owens, P.; Strope, S.; Sandhu, G.; Kallogjeri, D.; Kibel, A. | Hospital readmission after radical cystectomy for bladder cancer: Results of a population-based analysis | 2012 | *Journal of Urology* | Conference abstract |
| Newman, W. C.; Neal, D. W.; Hoh, B. L. | A new comorbidities index for risk stratification for treatment of unruptured cerebral aneurysms | 2016 | *J Neurosurg* | Full text unavailable |
| Ng, T. P.; Feng, L.; Chiam, P. C.; Kua, E. H. | Psychiatric morbidity and acute hospitalization in elderly people | 2006 | *Int Psychogeriatr* | The impact of SMI on HSU was not assessed |
| Noll, E. L.; Rothbard, A. B.; Hadley, T. R.; Hurford, M. O. | Quality of Diabetes Care Among Adult Medicaid Enrollees With Mental Disorders | 2016 | *Psychiatric Services* | Incorrect definition of SMI |
| Nossel, I. R.; Calmes, C. A.; Brown, C. H.; Kreyenbuhl, J.; Goldberg, R. W.; Fang, L. J.; Dixon, L. B. | Patterns of Emergency Department Use for Medical Conditions Among Persons With Serious Mental Illness | 2010 | *Psychiatric Services* | Wrong patient group |
| Nuno, T.; Skrepnek, G. | Predictors of inpatient mortality, charges, and length of stay among the very old with colon cancer in the United States | 2015 | *Health Econom* | Conference abstract |
| Olsson, I.; Dahl, A. A. | Personality problems are considerably associated with somatic morbidity and health care utilisation | 2009 | *European Psychiatry* | Incorrect definition of PD |
| Pan, Y. J.; Kuo, K. H.; Yeh, L. L. | Healthcare cost, service use and mortality in major psychiatric disorders in Taiwan | 2019 | *J Affect Disord* | Incorrect definition of SMI |
| Parian, A. M.; Chen, P. H.; Lazarev, M.; Limketkai, B. N. | Comorbid psychiatric diagnoses are common among IBD hospitalizations and increase health care utilization: A nationwide analysis | 2016 | *Gastroenterology* | Conference abstract |
| Patel, K.; Bhiv; kar, S.; Desai, R.; Antin, T. | The burden of psychiatric illnesses in adult patients with beta-thalassemia: a 5-year nationwide inpatient evaluation in the United States | 2019 | *Ann Hematol* | The impact of SMI on HSU was not assessed |
| Rajaee, S. S.; Kanim, L. E.; Bae, H. W. | National trends in revision spinal fusion in the USA: patient characteristics and complications | 2014 | *Bone Joint J* | The impact of SMI on HSU was not assessed |
| Ramkumar, P. N.; Gwam, C.; Navarro, S. M.; Haeberle, H. S.; Karnuta, J. M.; Delanois, R. E.; Mont, M. A. | Discharge to the skilled nursing facility: patient risk factors and perioperative outcomes after total knee arthroplasty | 2019 | *Ann Transl Med* | Wrong outcome |
| Rapp, S. R.; Parisi, S. A.; Wallace, C. E. | Comorbid psychiatric disorders in elderly medical patients: a 1-year prospective study | 1991 | *J Am Geriatr Soc* | The impact of SMI on HSU was not assessed |
| Rasoul, D.; Wong, S.; Chandran, S.; Uppal, H.; Sarma, J.; Potluri, R. | Psychiatric co-morbidities and tendencies in patients with non-ischaemic heart failure (NIHF) - A large observational cohort study spanning 14 years | 2016 | *Heart* | Conference abstract |
| Rehou, S.; Dolp, R.; McCann, M. R.; Jeschke, M. G. | Burn patients that exceed the average length of stay | 2018 | *Journal of Burn Care and Research* | Conference abstract |
| Ren, Z.; Hsu, D. Y.; Brieva, J.; Silverberg, N. B.; Langan, S. M.; Silverberg, J. I. | Hospitalization, inpatient burden and comorbidities associated with bullous pemphigoid in the U.S.A | 2017 | *British Journal of Dermatology* | The impact of SMI on HSU was not assessed |
| Ride, J.; Kasteridis, P.; Gutacker, N.; Aragon, M. J. A.; Jacobs, R. | Healthcare Costs for People with Serious Mental Illness in England: An Analysis of Costs Across Primary Care, Hospital Care, and Specialist Mental Healthcare | 2020 | *Appl Health Econ Health Policy* | Wrong patient group |
| Rossi, K. C.; Kim, A. M.; JettÃ©, N.; Yoo, J. Y.; Hung, K.; Dhamoon, M. S. | Increased risk of hospital admission for ICD-9-CM psychotic episodes following admission for epilepsy | 2018 | *Epilepsia* | Wrong outcome |
| Rothbard, A. B.; Metraux, S.; Blank, M. B. | Cost of care for Medicaid recipients with serious mental illness and HIV infection or AIDS | 2003 | *Psychiatric Services* | Incorrect definition of SMI |
| Rothbard, A. B.; Lee, S.; Blank, M. B. | Cost of treating seriously mentally ill persons with HIV following highly active retroviral therapy (HAART) | 2009 | *Journal of Mental Health Policy and Economics* | Incorrect definition of SMI |
| Rothbard, A. B.; Miller, K.; Lee, S.; Blank, M. B. | Revised cost estimates of Medicaid recipients with serious mental illness and HIV-AIDS | 2009 | *Psychiatric Services* | Incorrect definition of SMI |
| Rothrock, R. J.; Morra, R. P., Jr.; Deutsch, B. C.; Neifert, S. N.; Cho, S. K.; Caridi, J. M. | Effect of Psychiatric Comorbidities on In-Hospital Outcomes and Cost for Cervical Spondylotic Myelopathy | 2019 | *World Neurosurg* | Incorrect definition of SMI |
| Rumsfeld, Mary E. Plomondon; P. Michael Ho; Li, Wang; Gwendolyn, T. Greiner; James, H. Shore; Joseph, T. Sakai; Stephan, D. Fihn; John, S. | Severe mental illness and mortality of hospitalized ACS patients in the VHA | 2007 | *BMC Health Serv Res* | The impact of SMI on HSU was not assessed |
| Sajatovic, M.; Popli, A.; Semple, W. | Ten-year use of hospital-based services by geriatric veterans with schizophrenia and bipolar disorder | 1996 | *Psychiatric Services* | Full text unavailable |
| Salsberry, P. J.; Chipps, E.; Kennedy, C. | Use of general medical services among medicaid patients with severe and persistent mental illness | 2005 | *Psychiatric Services* | Wrong patient group |
| Savani, C.; Kumar, V.; Ghattas, J.; De Souza, L.; Rodriguez, J.; Chung, M.; Kesayan, T.; Kim, G.; Myers, M.; Brock, C. | Risk of worse short-term outcomes associated with hospitalization for migraine and co-morbid nationwide population-based cohort study | 2019 | *Neurology* | Conference abstract |
| Shen, Chan; Sambamoorthi, Usha; Rust, George | Co-occurring mental illness and health care utilization and expenditures in adults with obesity and chronic physical illness | 2008 | *Disease Management* | The impact of SMI on HSU was not assessed |
| Sicras-Mainar, A.; Navarro-Artieda, R.; IbÃ¡Ã±ez-Nolla, J.; PÃ©rez-Ronco, J. | Incidence, resource use and costs associated with postherpetic neuralgia: A population-based retrospective study | 2012 | *Revista de Neurologia* | Non-english article |
| Su, C. H.; Chiu, H. C.; Hsieh, H. M.; Yen, J. Y.; Lee, M. H.; Li, C. Y.; Chang, K. P.; Huang, C. J. | Healthcare Utilization and Expenditures for Persons with Diabetes Comorbid with Mental Illnesses | 2016 | *Psychiatric Quarterly* | The impact of SMI on HSU was not assessed |
| Szekendi, M.K.; Williams, M.V.; Carrier, D.; Hensley, L.; Thomas, S.; Cerese, J. | The characteristics of patients frequently admitted to academic medical centres in the United States | 2015 | *Journal of Hospital Medicine* | Wrong outcome |
| Taherian, M.; Monteith, T. S. | Inpatient migraine management: The impact of mood disorders on hospital outcomes (2002 to 2012) | 2016 | *Headache* | Conference abstract |
| Tanner, E.; Cholankeril, G.; Hu, M.; Somasundar, P. | Pre-operative predictors for skilled nursing facility placement in geriatric colon cancer patients | 2017 | *J Am Geriatr Soc* | Conference abstract |
| Tong, C.; Etter, K.; Bhattacharyya, S.; Shaw, R.; Do Rego, B. | Psu36 Predictors of Post-Acute Costs in Elective Total Knee Arthroplasty among Medicare Beneficiaries: A Tale of Two Models | 2019 | *Surgery* | Conference abstract |
| Wei, W.; G; hi, K.; Blauer-Peterson, C.; Johnson, J. | Impact of Pain Severity and Opioid Use on Health Care Resource Utilization and Costs Among Patients with Knee and Hip Osteoarthritis | 2019 | *J Manag Care Spec Pharm* | The impact of SMI on HSU was not assessed |
| Weisberg, D.; Chatterjee, P.; Bethea, E. D.; Yialamas, M.; Christopher, K. B. | Mortality after critical care hospitalization in patients with psychotic disorders | 2016 | *J Gen Intern Med* | Conference abstract |
| Wieghard, N. E.; Hart, K. D.; Herzig, D. O.; Lu, K. C.; Tsikitis, V. L. | Psychiatric Illness is a Disparity in the Surgical Management of Rectal Cancer | 2015 | *Annals of Surgical Oncology* | The impact of SMI on HSU was not assessed |
| Wolff, J.; Heister, T.; Normann, C.; Kaier, K. | Hospital costs associated with psychiatric comorbidities: a retrospective study | 2018 | *BMC Health Serv Res* | The impact of SMI on HSU was not assessed |
| Yue, B.; Fang, S.; Krittanawong, C.; Wei, X.; Khandaker, M.; Herzog, E. | Depression and psychosis are associated with shorter length of hospital stay in patients with stress (Takotsubo) cardiomyopathy: A nationwide analysis of 2014 | 2017 | *Circulation* | Conference abstract |
| Zatzick, D. F.; Kang, S. M.; Kim, S. Y.; Leigh, P.; Kravitz, R.; Drake, C.; Sue, S.; Wisner, D. | Patients with recognized psychiatric disorders in trauma surgery: Incidence, inpatient length of stay, and cost | 2000 | *Journal of Trauma-Injury Infection and Critical Care* | Patients under the age of 18 |
