# Supplementary material for: Severe mental illness and health service utilisation for nonpsychiatric medical disorders: A systematic review and meta-analysis
Source: PLoS Med. 2020 Sep 14;17(9):e1003284. doi: 10.1371/journal.pmed.1003284 (PMC7489517; doi:10.1371/journal.pmed.1003284)
Supplement: S4 Appendix — (DOCX) [file pmed.1003284.s004.docx]

**The impact of comorbid severe mental illness on non-psychiatric health service utilisation: A systematic review and meta-analysis**

**Appendix 4: Subgroup analyses**

Inpatient hospital admissions

| Subgroups | Analyses (n) | Test for overall effect | | | | Test for subgroup differences | | |
| --- | --- | --- | --- | --- | --- | --- | --- | --- |
|  |  | *Pooled OR (CI^95^)* | *I^2^* | *Z* | *p value* | *χ^2^* | *I^2^* | *p value* |
| All analyses | 17 | 1.84 (1.21-2.80) | 100% | 2.83 | 0.005 |  |  |  |
| Hospital admission type |  |  |  |  |  | 2.07 | 51.7% | 0.15 |
| *All-cause** | 8 | 2.34 (1.32-4.16) | 100% | 2.90 | 0.004 |  |  |  |
| *Medical* | 9 | 1.48 (1.15-2.80) | 99% | 3.05 | 0.002 |  |  |  |
| SMI type |  |  |  |  |  | 11.35 | 82.4% | **0.003** |
| *Schizophrenia* | 6 | 2.37 (1.09-5.16) | 100% | 2.18 | 0.03 |  |  |  |
| *Bipolar disorder* | 7 | 1.35 (1.07-1.71) | 98% | 2.54 | 0.01 |  |  |  |
| *Psychosis* | 4 | 2.14 (1.86-2.46) | 0% | 10.75 | <0.001 |  |  |  |
| Sample size |  |  |  |  |  | 0.03 | 0% | 0.87 |
| *≤1,131,375* | 9 | 1.83 (1.02-3.29) | 100% | 2.01 | 0.04 |  |  |  |
| *>1,131,375* | 8 | 1.92 (1.68-2.20) | 52% | 9,61 | <0.001 |  |  |  |
| Country |  |  |  |  |  | 3.32 | 69.9% | 0.07 |
| *Canada* | 4 | 3.25 (1.46-7.24) | 100% | 2.89 | 0.004 |  |  |  |
| *USA* | 13 | 1.52 (1.26-1.83) | 98% | 4.40 | <0.001 |  |  |  |
| *All-cause: psychiatric reasons for inpatient admission cannot be definitively ruled out | | | | | | | | |

Length of hospital stay (days)

| Subgroups | Analyses (n) | Test for overall effect | | | | Test for subgroup differences | | |
| --- | --- | --- | --- | --- | --- | --- | --- | --- |
|  |  | *Pooled SMD (CI^95^)* | *I^2^* | *Z* | *p value* | *χ^2^* | *I^2^* | *p value* |
| All analyses | 17 | 0.59 (0.36-0.83) | 100% | 4.93 | <0.001 |  |  |  |
| Hospital admission type |  |  |  |  |  | 0.67 | 0% | 0.41 |
| *All-cause** | 2 | 0.44 (0.11-0.76) | 99% | 2.65 | 0.008 |  |  |  |
| *Medical* | 15 | 0.62 (0.34-0.90) | 100% | 4.33 | <0.001 |  |  |  |
| Reason for admission |  |  |  |  |  | 0.73 | 0% | 0.39 |
| *Long-term condition* | 13 | 0.61 (0.24-0.99) | 100% | 3.22 | 0.001 |  |  |  |
| *Trauma* | 4 | 0.44 (0.33-0.56) | 75% | 7.65 | <0.001 |  |  |  |
| SMI type |  |  |  |  |  | 15.73 | 80.9% | **0.001** |
| *Schizophrenia* | 10 | 0.86 (0.50-1.21) | 100% | 4.78 | <0.001 |  |  |  |
| *Bipolar disorder* | 3 | 0.11 (-0.03-0.25) | 88% | 1.49 | 0.14 |  |  |  |
| *Psychosis* | 2 | 0.47 (-0.36-1.31) | 75% | 1.11 | 0.27 |  |  |  |
| *SMI*** | 2 | 0.13 (-0.16-0.42) | 98% | 0.88 | 0.38 |  |  |  |
| Sample size |  |  |  |  |  | 0.43 | 0% | 0.51 |
| *≤26,502* | 9 | 0.79 (-0.41-2.00 | 100% | 1.30 | 0.19 |  |  |  |
| *>26,502* | 8 | 0.39 (0.26-0.52) | 100% | 5.78 | <0.001 |  |  |  |
| Country |  |  |  |  |  | 1.76 | 43.2% | 0.18 |
| *USA* | 7 | 0.38 (0.28-0.49) | 98% | 7.05 | <0.001 |  |  |  |
| *Not USA* | 10 | 0.76 (0.22-1.30) | 100% | 2.74 | 0.006 |  |  |  |
| Study quality |  |  |  |  |  | 7.19 | 86.1% | **0.007** |
| *Good* | 5 | 0.20 (-0.09-0.49) | 98% | 1.35 | 0.18 |  |  |  |
| *Poor* | 12 | 0.76 (0.47-1.05) | 100% | 5.19 | <0.001 |  |  |  |
| *All-cause: psychiatric reasons for inpatient admission cannot be definitively ruled out  **SMI: a composite measure of severe mental illness, i.e. patients with schizophrenia, bipolar disorder, and/or psychosis were analysed together  SMD = standardised mean difference | | | | | | | | |

30-day hospital readmission

| Subgroups | Analyses (n) | Test for overall effect | | | | Test for subgroup differences | | |
| --- | --- | --- | --- | --- | --- | --- | --- | --- |
|  |  | *Pooled OR (CI^95^)* | *I^2^* | *Z* | *p value* | *χ^2^* | *I^2^* | *p value* |
| All analyses | 16 | 1.37 (1.28-1.47) | 83% | 8.90 | <0.001 |  |  |  |
| Hospital admission type |  |  |  |  |  | 0.50 | 0% | 0.48 |
| *All-cause** | 4 | 1.36 (1.22-1.52) | 83% | 5.57 | <0.001 |  |  |  |
| *Medical* | 12 | 1.44 (1.28-1.61) | 78% | 6.28 | <0.001 |  |  |  |
| SMI type |  |  |  |  |  | 1.98 | 49.4% | 0.16 |
| *Psychosis* | 12 | 1.35 (1.26-1.45) | 84% | 8.50 | <0.001 |  |  |  |
| *Other SMI* | 4 | 2.33 (1.09-4.99) | 84% | 2.19 | 0.03 |  |  |  |
| Sample size |  |  |  |  |  | 1.13 | 11.3% | 0.29 |
| *<298,751* | 8 | 1.45 (1.31-1.59) | 82% | 7.51 | <0.001 |  |  |  |
| *>298,751* | 8 | 1.33 (1.18-1.50) | 77% | 4.54 | <0.001 |  |  |  |
| Country |  |  |  |  |  | 13.89 | 92.8% | **<0.001** |
| *USA* | 9 | 1.28 (1.19-1.39) | 88% | 6.22 | <0.001 |  |  |  |
| *Europe* | 7 | 1.65 (1.48-1.85) | 0% | 9.04 | <0.001 |  |  |  |
| *All-cause: psychiatric reasons for inpatient admission cannot be definitively ruled out | | | | | | | | |
