# Supplementary material for: Severe mental illness and health service utilisation for nonpsychiatric medical disorders: A systematic review and meta-analysis
Source: PLoS Med. 2020 Sep 14;17(9):e1003284. doi: 10.1371/journal.pmed.1003284 (PMC7489517; doi:10.1371/journal.pmed.1003284)

**The impact of comorbid severe mental illness on non-psychiatric health service utilisation: A systematic review and meta-analysis**

**Appendix 4: Funnel plots**

Inpatient admissions


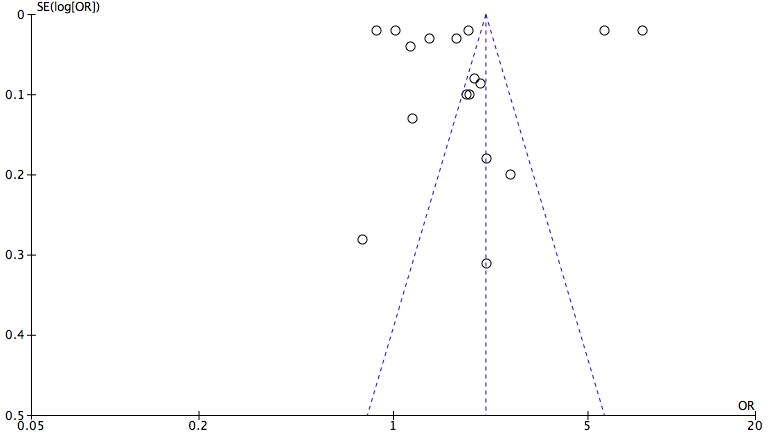


Length of hospital stay


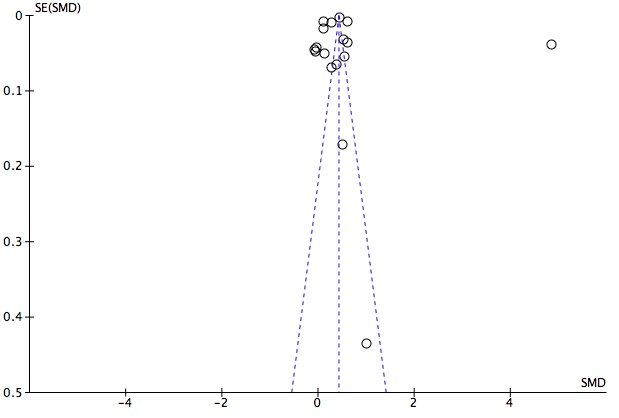


30-day hospital readmission


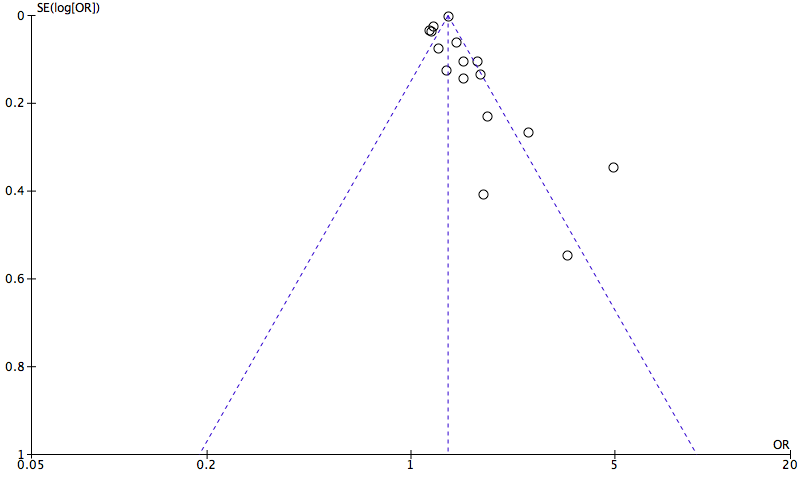


Use of emergency department


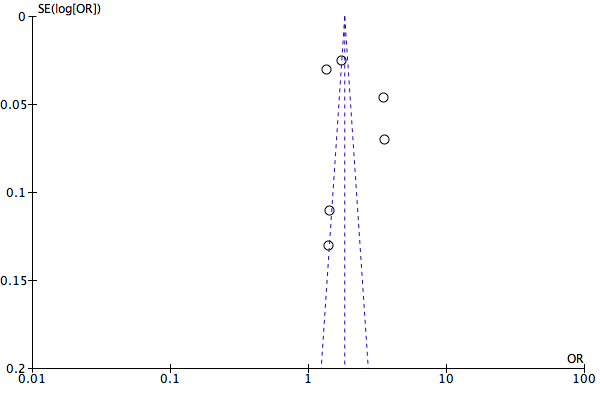

Supplement: S5 Appendix — (DOCX) [file pmed.1003284.s005.docx]
