# Supplementary material for: Severe mental illness and health service utilisation for nonpsychiatric medical disorders: A systematic review and meta-analysis
Source: PLoS Med. 2020 Sep 14;17(9):e1003284. doi: 10.1371/journal.pmed.1003284 (PMC7489517; doi:10.1371/journal.pmed.1003284)
Supplement: Attachment — Submitted filename: The impact of comorbid SMI on non-psych HSU_Reviewer3.docx [file pmed.1003284.s007.docx]

PLOS Medicine

The impact of severe mental illness on health service utilisation for non-psychiatric medical disorders: A systematic review and meta-analysis

--Manuscript Draft--

| Manuscript Number: | PMEDICINE-D-20-00254R1 |
| --- | --- |
| Full Title: | The impact of severe mental illness on health service utilisation for non-psychiatric medical disorders: A systematic review and meta-analysis |
| Short Title: | Severe mental illness and health service use: A systematic review and meta-analysis |
| Article Type: | Research Article |
| Abstract: | Summary Background  Psychiatric comorbidity is known to impact upon use of non-psychiatric health services. The aim of this systematic review and meta-analysis was to assess the specific impact of SMI on the use of inpatient, emergency, and primary care services for non- psychiatric medical disorders.  Methods  PubMed, Web of Science, PsychINFO, EMBASE, and The Cochrane Library were searched for relevant studies up to October 2018. Studies were included if they assessed the impact of SMI on non-psychiatric inpatient, emergency, and primary care service use in adults. Random effects meta-analyses of the effect of SMI on inpatient admissions, length of hospital stay, 30-day hospital readmission rates, and emergency department use were performed. This review protocol is registered in PROSPERO (CRD42019119516).  Results  61 studies were eligible for review. All were observational cohort studies. P atients with SMI were more than twice as likely to be admitted as non-psychiatric inpatients (pooled OR=2·02, CI 95 =1·29-3·15, p =0·002), had hospital stays that were increased by 0·74 days (pooled SMD=0·74 days, CI 95 =0·36-1.12, p =0·0002), were 33% more likely to be readmitted to hospital within 30 days (pooled OR=1·33, CI 95  =1·24-1·43, p <0·00001) and were more than twice as likely to attend the emergency department (pooled OR=2·10, CI 95 =1·45-3·03, p <0·0001) compared to patients without SMI. Narrative synthesis showed that SMI was associated with increased utilisation of primary care services.  Conclusions  SMI impacts significantly upon the use of non-psychiatric health services. Illustrating and quantifying this helps to build a case for and guide the delivery of system-wide integration of mental and physical health services. |
| Additional Information: |  |
| Question | Response |
| Financial Disclosure  Enter a financial disclosure statement that describes the sources of funding for the work included in this submission. Review the [submission guidelines](http://journals.plos.org/plosmedicine/s/submission-guidelines#loc-financial-disclosure-statement) for detailed requirements. View published research articles from [PLOS Medicine](http://journals.plos.org/plosmedicine/) for specific | The author(s) received no specific funding for this work |

| examples.  This statement is required for submission and will appear in the published article if the submission is accepted. Please make sure it is accurate. |  |
| --- | --- |
| Unfunded studies  Enter: The author(s) received no specific funding for this work.  Funded studies  Enter a statement with the following details:   - Initials of the authors who received each award - Grant numbers awarded to each author - The full name of each funder - URL of each funder website - Did the sponsors or funders play any role in the study design, data collection and analysis, decision to publish, or preparation of the manuscript? - NO - Include this sentence at the end of your statement: The funders had no role in study design, data collection and analysis, decision to publish, or preparation of the manuscript. - YES - Specify the role(s) played. |  |
| * typeset |  |
| Competing Interests  Use the instructions below to enter a competing interest statement for this submission. On behalf of all authors, disclose any [competing interests](http://journals.plos.org/plosmedicine/s/competing-interests) that could be perceived to bias this work—acknowledging all financial support and any other relevant financial or non- financial competing interests.  This statement will appear in the published article if the submission is accepted. Please make sure it is accurate. View published research articles from [PLOS Medicine](http://journals.plos.org/plosmedicine/) for specific examples. | The authors have declared that no competing interests exist. |

| NO authors have competing interests  Enter: The authors have declared that no competing interests exist.  Authors with competing interests  Enter competing interest details beginning with this statement:  I have read the journal's policy and the authors of this manuscript have the following competing interests: [insert competing interests here] |  |
| --- | --- |
| * typeset |  |
| Ethics Statement  You must include an Ethics Statement in the Methods section of your manuscript if your study involved:   - Human participants - Human specimens or tissue - Vertebrate animals or cephalopods - Vertebrate embryos or tissues - Field research   Not including a statement when required will delay the review process.  General guidance is provided below. Please consult the guidelines on human subjects research and animal research for detailed instructions. | I confirm that I have read and understood PLOS’ requirements for an Ethics Statement. |

| Format for specific study types  Human Subject Research (involving human participants and/or tissue)   - Give the name of the institutional review board or ethics committee that approved the study - Include the approval number and/or a statement indicating approval of this research - Indicate the form of consent obtained (written/oral) or the reason that consent was not obtained (e.g. the data were analyzed anonymously)   Animal Research (involving vertebrate animals, embryos or tissues)   - Provide the name of the Institutional Animal   Care and Use Committee (IACUC) or other relevant ethics board that reviewed the study protocol, and indicate whether they approved this research or granted a formal waiver of ethical approval   - Include an approval number if one was obtained - If the study involved non-human primates, add additional details about animal welfare and steps taken to ameliorate suffering   Field Research  Include the following details if this study involves the collection of plant, animal, or other materials from a natural setting:   - Field permit number - Name of the institution or relevant body that   granted permission |  |
| --- | --- |
| Please check the box to confirm your understanding of this policy, then read and agree to one of the statements. |  |
| Please respond:  as follow-up to "Ethics Statement  You must include an Ethics Statement in the Methods section of your manuscript if your study involved:   - Human participants | My study does not require an ethics statement. |

| - Human specimens or tissue - Vertebrate animals or cephalopods - Vertebrate embryos or tissues - Field research   Not including a statement when required will delay the review process.  General guidance is provided below. Please consult the guidelines on human subjects research and animal research for detailed instructions. |  |
| --- | --- |

| Format for specific study types  Human Subject Research (involving human participants and/or tissue)   - Give the name of the institutional review board or ethics committee that approved the study - Include the approval number and/or a statement indicating approval of this research - Indicate the form of consent obtained (written/oral) or the reason that consent was not obtained (e.g. the data were analyzed anonymously)   Animal Research (involving vertebrate animals, embryos or tissues)   - Provide the name of the Institutional Animal   Care and Use Committee (IACUC) or other relevant ethics board that reviewed the study protocol, and indicate whether they approved this research or granted a formal waiver of ethical approval   - Include an approval number if one was obtained - If the study involved non-human primates, add additional details about animal welfare and steps taken to ameliorate suffering   Field Research  Include the following details if this study involves the collection of plant, animal, or other materials from a natural setting:   - Field permit number - Name of the institution or relevant body that   granted permission |  |
| --- | --- |
| Please check the box to confirm your understanding of this policy, then read and agree to one of the statements. " |  |
| Data Availability  Authors are required to make all data underlying the findings described fully available, without restriction, and from the time of publication. PLOS allows rare exceptions to address legal and ethical concerns. See the [PLOS Data Policy](http://journals.plos.org/plosmedicine/s/data-availability) and | Yes - all data are fully available without restriction |

| [FAQ](http://journals.plos.org/plosmedicine/s/data-availability#loc-faqs-for-data-policy) for detailed information.  A Data Availability Statement describing where the data can be found is required at submission. Your answers to this question constitute the Data Availability Statement and will be published in the article, if accepted. |  |
| --- | --- |
| Important: Stating ‘data available on request from the author’ is not sufficient. If your data are only available upon request, select ‘No’ for the first question and explain your exceptional situation in the text box. |  |
| Do the authors confirm that all data underlying the findings described in their manuscript are fully available without restriction? |  |
| Describe where the data may be found in full sentences. If you are copying our sample text, replace any instances of XXX with the appropriate details.   - If the data are held or will be held in a public repository, include URLs, accession numbers or DOIs. If this information will only be available after acceptance, indicate this by ticking the box below. For example: All XXX files are available from the XXX database (accession number(s) XXX, XXX.). - If the data are all contained within the manuscript and/or Supporting Information files, enter the following: All relevant data are within the manuscript and its Supporting Information files. - If neither of these applies but you are able to provide details of access elsewhere, with or without limitations, please do so. For example:   Data cannot be shared publicly because of [XXX]. Data are available from the XXX Institutional Data Access / Ethics Committee (contact via XXX) for | As this is a systematic review of existing literature all relevant data are within the manuscript and its Supporting Information files. |

| researchers who meet the criteria for access to confidential data.  The data underlying the results presented in the study are available from (include the name of the third party and contact information or URL).   - This text is appropriate if the data are owned by a third party and authors do not have permission to share the data.   * typeset |  |
| --- | --- |
| Additional data availability information: | Tick here if the URLs/accession numbers/DOIs will be available only after acceptance of the manuscript for publication so that we can ensure their inclusion before publication. |

Main manuscript	[Click here to access/download;Manuscript;The impact of comorbid SMI on non-psych HSU_PLOSMedicine.docx](https://www.editorialmanager.com/pmedicine/download.aspx?id=651022&guid=ad13375e-f311-4c1d-a907-0ad7e639c6f8&scheme=1)

# The impact of severe mental illness on health service utilisation for non-psychiatric medical disorders: A systematic review and meta-analysis

Amy Ronaldson PhD^1,2*^, Lotte Elton MBBS MSc^1^, Simone Jayakumar MSc^1^, Anna Jieman BSc^1^, Kristoffer Halvorsrud PhD^1^, Kamaldeep Bhui CBE MD FRCPsych^1^

^1^Centre for Psychiatry, Wolfson Institute of Preventive Medicine, Barts & The London School of Medicine, Old Anatomy Building, Charterhouse Square, London EC1M 6BQ. A Collaborating Centre of the World Psychiatric Association.

^2^Department of Psychological Medicine, Institute of Psychiatry, Psychology and Neuroscience, King’s College London, De Crespigny Park, London SE5 8AF

**Word count:** 4,502

**Number of figures and tables:** 10

***Corresponding author and person to whom reprint requests should be addressed:**

Dr Amy Ronaldson, Department of Psychological Medicine, Institute of Psychiatry, Psychology and Neuroscience, King’s College London, De Crespigny Park, London SE5 8AF

[amy.ronaldson@kcl.ac.uk](mailto:amy.ronaldson@kcl.ac.uk)

**Author contact details:**

1st author: [amy.ronaldson@kcl.ac.uk](mailto:amy.ronaldson@kcl.ac.uk) 2nd author: [l.elton@qmul.ac.uk](mailto:l.elton@qmul.ac.uk)

3rd author: [simone.jayakumar@qmul.ac.uk](mailto:simone.jayakumar@qmul.ac.uk) 4th author: [anna-theresa.jieman.19@ucl.ac.uk](mailto:anna-theresa.jieman.19@ucl.ac.uk) 5th author: [k.halvorsrud@qmul.ac.uk](mailto:k.halvorsrud@qmul.ac.uk)

6th author: [k.s.bhui@qmul.ac.uk](mailto:k.s.bhui@qmul.ac.uk)

**Role of the funding source:** There was no funding source for the study

**Summary**

**Background:** Psychiatric comorbidity is known to impact upon use of non-psychiatric health services. The aim of this systematic review and meta-analysis was to assess the specific impact of SMI on the use of inpatient, emergency, and primary care services for non-psychiatric medical disorders.

**Methods:** PubMed, Web of Science, PsychINFO, EMBASE, and The Cochrane Library were searched for relevant studies up to October 2018. Studies were included if they assessed the impact of SMI on non-psychiatric inpatient, emergency, and primary care service use in adults. Random effects meta-analyses of the effect of SMI on inpatient admissions, length of hospital stay, 30-day hospital readmission rates, and emergency department use were performed. This review protocol is registered in PROSPERO (CRD42019119516).

**Results:** 61 studies were eligible for review. All were observational cohort studies. **P**atients with SMI were more than twice as likely to be admitted as non-psychiatric inpatients (pooled OR=2·02, CI^95^=1·29-3·15, *p* =0·002), had hospital stays that were increased by 0·74 days (pooled SMD=0·74 days, CI^95^=0·36-1.12, *p*=0·0002), were 33% more likely to be readmitted to hospital within 30 days (pooled OR=1·33, CI^95^=1·24-1·43, *p*<0·00001) and were more than twice as likely to attend the emergency department (pooled OR=2·10, CI^95^=1·45-3·03, *p*<0·0001) compared to patients without SMI. Narrative synthesis showed that SMI was associated with increased utilisation of primary care services.

**Conclusions:** SMI impacts significantly upon the use of non-psychiatric health services. Illustrating and quantifying this helps to build a case for and guide the delivery of system-wide integration of mental and physical health services.

**Keywords:** Severe mental illness; schizophrenia; psychosis; bipolar disorder; health service utilisation; inpatient services; emergency department; primary care

**Background**

Mental health conditions are associated with high disease burden, poor overall health outcomes, and high health service utilisation (1–4). Arguably, increased health service utilisation could be attributed to appropriate use of psychological and psychiatric services. However, an early review (1994) of the literature found that psychiatric comorbidity (particularly cognitive and organic mental disorders) was associated with increased length of stay in the general hospital (5). Building on this, a later review (2005) assessed nonorganic common mental disorders and found that depression was associated with higher use of general medical services (6). The most recent review (2018) found that medical inpatients with any psychiatric comorbidity had longer hospital stays, higher medical costs, and more readmission than inpatients without (7).

The evidence to date suggests that psychiatric comorbidity is associated with increased utilisation of general medical services. However, the impact of severe mental illness (SMI) on the use of general medical services has yet to be systematically reviewed. People with SMI, such as schizophrenia or psychotic disorder, are more likely to develop chronic physical illness than the general population (8) and the impact of physical illness on people with SMI is significantly greater (9). It is probable that this affects the use of non-psychiatric general medical services in this patient group. Moreover, there are serious inequalities in the provision of physical healthcare for patients with SMI (8–10) which likely has repercussions for how they use general medical services.

As part of a series of systematic reviews, the present study focused on the impact of SMI on the use of non-psychiatric inpatient, emergency and primary care services for patients with medical disorders. Where possible, meta-analysis was used to determine the effect that SMI had on specific outcomes. The importance of this review lies in highlighting the extent to which SMI impacts upon non-psychiatric services, thereby building a case for system-wide integration of medical and mental healthcare. Results from meta-analyses might be used by policy makers and health economists to improve delivery of integrated care.

**Methods**

This review protocol is registered in the PROSPERO International Prospective Register of Systematic Reviews (<https://www.crd.york.ac.uk/PROSPERO/>) (CRD42019119516). The protocol conforms to the Preferred Reporting Items for Systematic Reviews and Meta-Analysis

(PRISMA) guidelines. This research is part of a larger systematic review assessing the impact of SMI and personality disorder on non-psychiatric health service utilisation. The present study focused on the impact of SMI on inpatient, emergency, and primary care service use.

# Search strategy and selection criteria

We searched PUBMED, Web of Science, PsycINFO, EMBASE, and The Cochrane Library for relevant studies with no publication date restrictions (See Appendix 1 for the full search strategy for each database searched). The search was supplemented with hand searches of journals related to the field, and reference sections of relevant papers. Searches were carried out between 26th October 2018 and 2nd November 2018. The search strategy was developed and conducted by AR.

Different definitions of SMI exist, but it generally refers to illnesses associated with psychosis as adopted by the Quality and Outcomes Framework of the UK National Health Service (11). Therefore, in this review SMI included bipolar disorder, psychosis, schizophrenia, and/or schizoaffective disorder. SMI did not include major depressive disorder (MDD). Studies that included MDD in their definition of SMI were excluded unless results were presented separately for each SMI subtype. Patients with physical health conditions and/or receiving medical treatment who did not have SMI served as controls.

Health service utilisation for medical non-psychiatric disorders was the primary outcome. The current paper focused on inpatient, emergency and primary care service use. We excluded studies for service use related to psychiatric, psychological, mental, or behavioural disorders. We included observational cohort and case-control studies, and randomised controlled trials. We excluded reviews, case reports, and studies that used qualitative methods only.

For a study to be included, patients had to be 16 years or older. Where it was unstated whether all patients were over 16 years, the decision to include the study was based on the likelihood of the index medical condition occurring in a paediatric sample. For example, a sample of stroke patients will unlikely have a sizeable paediatric sample and would therefore be included, whereas a sample of patients undergoing trauma surgery could have a sizeable paediatric sample and would be excluded. All studies had to be published in peer-reviewed journals. Non- English language articles were excluded. Conference proceedings were also excluded. Where

conference proceedings emerged in the search, authors were contacted to ascertain whether the data had been published in a peer-reviewed journal.

Articles were independently screened in two stages: a title and abstract screen (AR, AJ), followed by the retrieval and screening of potentially relevant full-text articles by two reviewers using the criteria listed above (AR, LE). Inter-rater reliability for the full-text screen was assessed using Cohen’s kappa, which indicated moderate levels of agreement between the reviewers (κ 0·55, 77·6% agreement). Conflicts were resolved through discussion.

# Data extraction and quality assessment

Data were extracted by two reviewers (AR, SJ): AR extracted the data from the publications and SJ cross-checked 10% (n=8) of the extracted studies for accuracy. There was acceptable agreement on extraction (intraclass correlation coefficient = 0·63). Sample characteristics, methodological characteristics, and main health service utilisation outcomes were extracted. The data extraction tables were piloted and refined before extraction began. As all studies included in the review were observational studies, the Newcastle-Ottawa Scale (NOS) was used to assess the quality of each study (12). The NOS assesses the quality of each study using a system where ‘stars’ are awarded on three broad categories: selection of groups, comparability of groups, and discernment of the outcome of interest for the case-control or cohort. Each article is rated on nine variables and can earn a maximum of nine stars. More stars indicate less risk of bias in a given study, and the number of stars awarded allows a study to be deemed of good, fair, or poor quality. Due to the nature of the current review, if a study did not adjust for severity of physical illness and/or the presence of physical comorbidities (e.g. Charlson Comorbidity Index (13), Elixhauser Comorbidity Index (14), etc.) it was deemed to be of poor quality, regardless of the number of NOS ‘stars’ acquired. Quality assessment was carried out with the outcome of interest in mind, i.e. if a study had several clinical outcomes alongside a health service use outcome, the quality of the study would be assessed based on the health service use outcome.

# Data analysis

For all outcomes (inpatient service use, emergency service use, and primary care use) a narrative synthesis was carried out.

Meta-analysis and subgroup analysis were performed using Review Manager 5.3 of the Cochrane Collaboration (15). In all meta-analyses we used a random effects model since this model estimates effects while considering the heterogeneity between studies. For studies that reported continuous data, only those that provided both means and standard deviations/standard errors were included in the meta-analysis. Where required, standard deviations were calculated from confidence intervals using a verified formula (16). For studies that reported the likelihood of an outcome occurring, only adjusted studies (physical comorbidities/illness severity + other relevant factors) that provided odds ratios and 95% confidence intervals were included in the meta-analysis. All odds ratios and confidence interval limits were log-transformed (natural log). Standard error was then calculated using a verified formula (16).

Higgin’s *I2* was used to assess heterogeneity between studies. Where considerable heterogeneity is present (*I2* ≥ 90%)(16) statistical pooling is usually deemed inappropriate. However, we have reported pooled effect sizes for ease of interpretation even in cases where there was considerable heterogeneity due to the clinical relevance of results. These should be interpreted with caution.

Sources of heterogeneity were investigated using subgroup analysis. Sources investigated included type of health service use outcome (all-cause (i.e. psychiatric treatment is unlikely but cannot be definitively ruled out) versus medical), type of SMI, sample size (cut-off determined by median split), country where the study took place, and study quality where applicable. We assessed the degree of publication bias by visual examination of funnel plots. Publication bias was deemed to be absent if the plot showed an inverted symmetrical funnel. Where appropriate,

i.e. at least 10 studies included in meta-analysis (16), Egger’s test for funnel plot asymmetry was carried out (17). In all analyses, statistical significance was set at *p*≤ 0·05.

**Results**

**Study selection**

The systematic literature search resulted in a total of 4620 articles. After removing duplicates 3507 articles remained. Preliminary hand searches of journals and reference sections of relevant papers identified 8 more articles. After reviewing the titles and abstracts of these 3515

articles, a total of 196 articles were included for the full-text review (see PRISMA diagram in Figure 1). A list of excluded articles is provided in Appendix 2.

Of seventy-two eligible studies, sixty-one entered the review as these assessed the impact of SMI on inpatient, emergency, and/or primary care services. All were observational cohort studies, and most were retrospective cohorts where health service utilisation was compared between patients with and without SMI over time (n=52); some studies adopted matched case- control designs (n=9). Thirty-eight studies were deemed to be of good quality, two were fair, and 21 were poor.

The number of participants in the reviewed studies ranged from 27 to 10,777,210. The majority of the studies were from the United States (US: n=43), with the remainder in the United Kingdom (UK: n=6), Canada (n=3), Denmark (n=2), Taiwan (n=2), Australia (n=2), Israel (n=1), Japan, (n=1), and Sweden (n=1). The majority of studies were carried out in patients with specific medical index disorders (n=46). The most common medical index disorders were diabetes (n=10), heart failure (n=7), and total joint (knee and/or hip) arthroplasty (n=5). The remaining studies (n=15) were carried out in the general medical population, e.g. patients admitted to general medicine departments (18,19); all residents of nursing homes in Florida (Becker et al., 2010); all people on Taiwanese National Health Research Institute Database (21). Study periods ranged from 12 months to 18 years.

# The impact of severe mental illness on the use of non-psychiatric medical inpatient services

*Inpatient hospital admissions*

Table 1 describes the 21 studies (comprising 36 separate analyses) of the impact of SMI on non-psychiatric medical inpatient admissions (20–40). In 29 of these analyses, having SMI was associated with increased inpatient hospital admissions over the study period (12 months to 15·5 years). Seven analyses from four studies produced non-significant results (23,28,33,40) and one analysis revealed that in residents of assisted living facilities under 65 years, major psychotic disorder was associated with a reduced likelihood of hospitalisation for ambulatory care sensitive conditions (ACSCs, i.e. conditions where effective case management would prevent hospital admissions such as asthma, angina, and epilepsy)(23).

Results from 13 analyses from seven studies were deemed potentially appropriate for inclusion in a meta-analysis (22,24,26,31,33,34,40), i.e. they adjusted for physical comorbidities/illness severity and other relevant factors, and presented odds ratios relating to the likelihood of inpatient hospitalisation over the study period. However, Higgin’s *I2* indicated that the set of studies were extremely heterogeneous (χ2 (12)=5671·22, *p*<0·00001, *I^2^*=100%), therefore the estimation of the overall pooled effect should be interpreted with caution. The pooled odds ratio indicated that patients with SMI were more than twice as likely to be admitted as a non- psychiatric inpatient than patients without SMI (pooled OR=2·02, CI^95^=1·29-3·15, *p* =0·002; Figure 2).

In order to determine the source of heterogeneity, we examined estimates between analyses that examined all-cause hospital admissions versus medical admissions, SMI subtype, sample size (determined by median split; n>155,312 versus n≤155,312), and analyses in the USA versus Canada. None explained heterogeneity (see Appendix 3 for tables detailing subgroup analyses statistics). Study quality was not considered to be a potential source of heterogeneity as all but one study included in the meta-analysis was of good quality. Visual examination of the funnel plot (see Appendix 4) did not indicate significant publication bias but as only seven studies were included this could not be confirmed using Egger’s test (16).

*Length of hospital stay*

Table 2 describes the 26 studies (containing 33 separate analyses) that assessed the impact of SMI on non-psychiatric length of hospital stay (LOS) (18,19,21,28–30,32,38,39,41–57). In 26 of the 33 analyses, SMI was associated with increased LOS. Six analyses reported no significant associations and one study found SMI associated with shorter hospital stays (29).

Twelve studies (13 analyses) were potentially suitable for meta-analysis as they reported the mean LOS (and standard deviation or confidence intervals) for patients with and without SMI (21,28,30,39,42,47,48,51,52,54,55,57). The Higgin’s *I2* indicated that the set of studies were extremely heterogeneous (χ^2^(12)=13439·59, p<0·00001, *I^2^*=100%), therefore the estimation of the overall pooled effect should be interpreted with caution. The pooled standardised mean difference (SMD) indicated that non-psychiatric LOS was 0.74 days longer for patients with SMI compared to patients without (pooled SMD=0·74 days, CI^95^=0·36-1·12, *p*=0·0002; Figure 3).

Sources of potential heterogeneity were investigated using subgroup analysis (see Appendix 3). No significant sources of variance emerged. Visual examination of the funnel plot (see Appendix 4) suggested publication bias, but this was not confirmed by Egger’s test (*p*=0·269).

*Hospital readmission rates*

Table 3 describes the 17 studies (31 separate analyses) included in the review that examined the impact of SMI on non-psychiatric hospital readmission rates (27,58–73). In 27 of these analyses, SMI was associated with increased hospital readmissions. Four studies showed no significant association (61,63,65,66).

Nine studies (13 analyses) were suitable for meta-analysis (59,60,64,66,68–71,73). These studies examined the impact of SMI on 30-day readmission rates specifically, except for one study that looked at 28-day readmission rates (66). Higgin’s *I2* (χ^2^(12)=74·8, p<0·00001, *I^2^*=84%) suggested that meta-analysis was appropriate (16). The pooled odds ratio indicated that patients with SMI were 33% more likely than patients without SMI to be readmitted to hospital within 30 days of the index medical hospitalisation (pooled OR=1·33, CI^95^=1·24-1·43, *p*<0·00001; Figure 4).

Although meta-analysis was appropriate, there was still considerable heterogeneity between studies examining 30-day readmission rates. Geographical area (test for subgroup differences: χ^2^(1)=7·40, *p*=0·007) was a significant sources of variance between studies (see Appendix 3). Studies carried out in the USA indicated that patients with comorbid SMI were 28% more likely to be readmitted to hospital within 30 days (OR=1·28, CI^95^=1·19-1·39, *p*<0·00001) whereas studies carried out in Europe (UK and Denmark) indicated that SMI patients were 62% more likely to be readmitted (OR=1·62, CI^95^=1·40-1·88, *p*<0·00001). As all studies included in the meta-analysis were of good quality, study quality was not deemed to be a source of heterogeneity. Visual examination of the funnel plot (see Appendix 4) indicated that publication bias was likely, but as only nine studies were included, Egger’s test of publication bias was inappropriate.

**The impact of severe mental illness on emergency department use for medical disorders** Table 4 describes the 10 studies (15 analyses) included in the review that assessed the impact of SMI on the use of emergency care (21,24,29,31,32,37,61,67,74,75). In 14 out of 15 of the analyses, SMI was associated with increased use of emergency departments. This was true irrespective of adjustments for severity of medical disorders. One exception was Clark et al.’s

report that schizophrenia was not associated with emergency care for patients with alcohol withdrawal and previous admissions to medical intensive care units (61).

Meta-analysis was suitable for three studies (five analyses) (24,31,74). The Higgin’s *I2* value indicated that there was extreme variation between studies (χ^2^(4)=338·10, p<0·00001, *I^2^*=99%), therefore the estimation of the overall pooled effect should be interpreted with caution. The pooled odds ratio indicated that patients with SMI were more than twice as likely to attend the emergency department than patients without (pooled OR=2·10, CI^95^= 1·45-3·03, *p*<0·0001; Figure 5). Due to the small number of analyses included in the meta-analysis, subgroup analysis to determine sources of heterogeneity was not appropriate (16). All studies were of good quality meaning this did not contribute to the variation between them. Examination of the funnel plot indicated that publication bias was possible (see Appendix 4). Egger’s test of publication bias was inappropriate due to the number of studies included.

# The impact of comorbid severe mental illness on use of primary care

Six studies (8 separate analyses) looked at the impact of SMI on the use of primary care services (table 5) (24,29,74,76–78). Out of the eight analyses, six found that SMI was associated with increased primary care use. Five studies were of good quality and adjusted for physical illness severity, with the exception of Copeland et al who performed a cluster analysis (76). One study found that there was no significant effect of SMI on primary care use in epilepsy patients (74). Lichstein and colleagues reported that patients with schizophrenia were less likely to use medical homes for medical disorders (a model of primary care in the US) compared with patients without schizophrenia or depression (78). Due to heterogeneity across outcomes, meta- analysis was not possible.

**Discussion**

This systematic review and meta-analysis aimed to understand the impact of SMI on use of general inpatient, emergency, and primary care services. The evidence showed that SMI leads to increased use of general medical services. The results of this review highlight the extent to which patients with SMI need targeted and effective interventions, and system-wide integrated mental and physical healthcare.

The majority of studies indicated that non-psychiatric inpatient admissions, LOS, hospital readmission rates, and emergency department use were increased in medical patients with SMI compared to patients without SMI. This was confirmed with meta-analyses which showed that patients with SMI were more than

twice as likely to have an inpatient admission, had hospital stays that were increased by 0·74 days, and were 33% more likely to be readmitted within 30 days compared to patients without SMI. Meta-analysis also showed that patients with SMI were more than twice as likely to attend the emergency department. Most studies included in this review also showed that SMI was associated with increased use of primary care services. These findings are in line with previous reviews that have shown that health service utilisation is increased in patients with psychiatric comorbidity (5–7). However, this is the first time that the impact of SMI on the use of general medical services has been systematically reviewed and meta-analysed.

There are several factors that might explain why patients with SMI are using non-psychiatric healthcare services more than those without. The primary reason for increased service use is likely the considerable rates of physical illness seen in patients with SMI. Significant increases in levels of obesity, metabolic syndrome, diabetes, cardiovascular disease, viral disease, respiratory tract disease, and musculoskeletal disease are seen alongside SMI, and illness severity is usually more pronounced in these patients (9). Increased morbidity in SMI patients is largely down to a higher prevalence of modifiable risk factors (79), such as smoking (80), obesity (81), and alcohol and substance misuse (82). Use of psychotropic medications have also been associated with increased obesity, and antipsychotic medication is known to be diabetogenic (9,83). There is also evidence that disparities exist in the provision of healthcare for people with SMI (10). Psychiatric symptoms may prevent the patient from seeking adequate physical healthcare, and stigmatisation of patients can lead to diagnostic overshadowing and a lack of appropriate care and screening (84). A lack of adequate physical healthcare for patients with SMI means that their physical symptoms are likely to be much worse when they finally present in general medical services, potentially leading to an increase in use of primary care services, emergency department attendances, hospital admissions, and readmissions. This would likely impact upon LOS, but LOS in patients with SMI could also be affected by socioeconomic circumstances (85).

The majority of studies included in this review were of good quality with large sample sizes. In most cases SMI was defined using established diagnostic codes, and health service utilisation outcomes were obtained using medical record linkage. However, some studies (34%) were rated as poor quality largely down to failure to control for physical illness severity or the presence of comorbidities, meaning the strength of evidence differed across studies. Nevertheless, most studies showed that SMI led to an increase in the use of general medical

services and study quality was not a significant source of heterogeneity in the meta-analyses indicating that risk of bias did not seem to affect the overall findings of the review. However, most studies included in the meta-analysis looking at length of hospital stay were of poor quality which should be taken into account when interpreting the result. Only peer-reviewed publications were included in the review meaning that publication bias was likely, although examination of funnel plots indicated this was only the case for studies of 30-day hospital readmissions. Despite carrying out literature searches on several databases using comprehensive search strategies, it is possible that retrieval of all relevant research was not complete.

I2 values indicated considerable heterogeneity across studies. This was to be expected in a review of this kind where there is large variation between studies in terms of patient population, hospital setting, and health system. Examining sources of variation revealed that there were differences between the US and European health systems in terms of readmission rates. No other factors emerged as significant sources of heterogeneity across studies. Other patient and clinical characteristics likely explain the bulk of heterogeneity. This means that there is uncertainty around the magnitude of the impact of SMI on non-psychiatric health service utilisation and results of this review should be interpreted with this in mind.

Although the focus of this review was on non-psychiatric health service utilisation, it was not always possible to determine whether the health service outcome definitively excluded mental health treatment (e.g. use of the emergency department). Due to the nature of primary care it was not possible to rule out the use of primary care services for psychiatric reasons in any of the studies included in the review. However, a recent study reported that nine out of 10 of the most common patient-reported reasons for primary care visits were non-psychiatric (86) indicating that this outcome was likely to be mainly non-psychiatric.

**Conclusions**

The results of this systematic review and meta-analysis highlight the extent to which SMI impacts upon general medical services. Patients with SMI were more than twice as likely to have an inpatient admission, had hospital stays that were increased by 0·74 days, were 33% more likely to be readmitted within 30 days, and were more than twice as likely to attend the emergency department compared to patients without SMI. Most studies included in this review also showed that SMI was associated with increased use of primary care services. Illustrating

and quantifying this helps to build a case for system-wide integration of mental and physical healthcare. Additionally, the results of the meta-analyses might be used to guide clinicians, health economists, policy makers, and commissioners in the improvement of the delivery of physical healthcare for SMI patients. The Five year Forward View for Mental Health for NHS England aims to improve early detection of physical illness in SMI patients through the implementation of physical healthcare screening, assessment, and intervention (87). This is to be delivered across both primary and secondary care. Prevention, early detection, and timely delivery of treatment for physical illness will hopefully improve the physical health of patients with SMI and reduce use of non-psychiatric healthcare services.

**Declarations**

**Ethics approval and consent to participate**

Not applicable

# Consent for publication

Not applicable

# Availability of data and materials

The authors declare that the data supporting the findings of this study are available within the article [and its supplementary information files]

# Competing interests

The authors declare that they have no competing interests

# Funding

No funding source

# Author’s contribution

AR conceived the study. AR, AJ, LE, and SJ did the systematic search, selected papers, and extracted data. AR did the statistical analysis with help from KH. AR drafted the manuscript with critical revisions from all authors. KB provided supervision throughout. All authors reviewed the paper before submission.

**References**

1. Murray CJ, Richards MA, Newton JN, Fenton KA, Anderson HR, Atkinson C, et al. UK health performance: findings of the Global Burden of Disease Study 2010. The Lancet. 2013 Mar 29;381(9871):997–1020.
2. Wolff J, Heister T, Normann C, Kaier K. Hospital costs associated with psychiatric comorbidities: a retrospective study. BMC Health Serv Res. 2018 30;18(1):67.
3. de Oliveira C, Cheng J, Vigod S, Rehm J, Kurdyak P. Patients With High Mental Health Costs Incur Over 30 Percent More Costs Than Other High-Cost Patients. Health Affairs. 2016 Jan 1;35(1):36–43.
4. Hajek A, Bock J-O, König H-H. Association of general psychological factors with frequent attendance in primary care: a population-based cross-sectional observational study. BMC Fam Pract. 2017 Mar 24;18(1):48.
5. Saravay SM, Lavin M. Psychiatric comorbidity and length of stay in the general hospital. A critical review of outcome studies. Psychosomatics. 1994 Jun;35(3):233–52.
6. Koopmans GT, Donker MCH, Rutten FHH. Length of hospital stay and health services use of medical inpatients with comorbid noncognitive mental disorders: a review of the literature. Gen Hosp Psychiatry. 2005 Feb;27(1):44–56.
7. Jansen L, van Schijndel M, van Waarde J, van Busschbach J. Health-economic outcomes in hospital patients with medical-psychiatric comorbidity: A systematic review and meta-analysis. PLoS ONE. 2018;13(3):e0194029.
8. Viron MJ, Stern TA. The impact of serious mental illness on health and healthcare. Psychosomatics. 2010 Dec;51(6):458–65.
9. De Hert M, Correll CU, Bobes J, Cetkovich-Bakmas M, Cohen D, Asai I, et al. Physical illness in patients with severe mental disorders. I. Prevalence, impact of medications and disparities in health care. World Psychiatry. 2011 Feb;10(1):52–77.
10. Lawrence D, Kisely S. Inequalities in healthcare provision for people with severe mental illness. J Psychopharmacol. 2010 Nov;24(4_supplement):61–8.
11. Olier I, Springate DA, Ashcroft DM, Doran T, Reeves D, Planner C, et al. Modelling Conditions and Health Care Processes in Electronic Health Records: An Application to Severe Mental Illness with the Clinical Practice Research Datalink. PLOS ONE. 2016 Feb 26;11(2):e0146715.
12. Wells G, Shea B, O’Connell D, Peterson J, Welch V, Losos M, et al. The Newcastle-Ottawa Scale (NOS) for assessing the quality of non-randomised studies in meta-analyses [Internet]. Canada; 2012. Available from: <http://www.ohri.ca/programs/clinical_epidemiology/oxford.asp>
13. Charlson ME, Pompei P, Ales KL, MacKenzie CR. A new method of classifying prognostic comorbidity in longitudinal studies: development and validation. J Chronic Dis. 1987;40(5):373–83.
14. Elixhauser A, Steiner C, Harris DR, Coffey RM. Comorbidity measures for use with administrative data. Med Care. 1998 Jan;36(1):8–27.
15. Review Manager (RevMan) [computer program]. Copenhagen: The Nordic Cochrane Centre: The Cochrane Collaboration; 2014.
16. The Cochrane Collaboration. Cochrane Handbook for Systematic Reviews of Interventions [Internet]. 2011 [cited 2019 Sep 9]. Report No.: Version 5.1.0. Available from: https://handbook-5-1.cochrane.org/
17. Egger M, Davey Smith G, Schneider M, Minder C. Bias in meta-analysis detected by a simple, graphical test. BMJ. 1997 Sep 13;315(7109):629–34.
18. Briskman I, Bar G, Boaz M, Shargorodsky M. Impact of co-morbid mental illness on the diagnosis and management of patients hospitalized for medical conditions in a general hospital. International Journal of Psychiatry in Medicine. 2012;43(4):339–48.
19. Bressi SK, Marcus SC, Solomon PL. The impact of psychiatric comorbidity on general hospital length of stay. Psychiatric Quarterly. 2006;77(3):203–9.
20. Becker MA, Boaz TL, Andel R, Gum AM, Papadopoulos AS. Predictors of Preventable Nursing Home Hospitalizations: The Role of Mental Disorders and Dementia. Am J Geriatr Psychiatr. 2010 Jun;18(6):475–82.
21. Hsieh MH, Tang CH, Hsieh MH, Lee IH, Lai TJ, Lin YJ, et al. Medical costs and vasculometabolic comorbidities among patients with bipolar disorder in Taiwan - A population-based and matched-control study. Journal of Affective Disorders. 2012;141(2–3):449–56.
22. Basta MN, Fox JP, Kanchwala SK, Wu LC, Serletti JM, Kovach SJ, et al. Complicated breast cancer-related lymphedema: Evaluating health care resource utilization and associated costs of management. American Journal of Surgery. 2016;211(1):133–41.
23. Becker M, Boaz T, Andel R, DeMuth A. Predictors of Avoidable Hospitalizations Among Assisted Living Residents. J Am Med Dir Assoc. 2012 May;13(4):355–9.
24. Bresee LC, Majumdar SR, Patten SB, Johnson JA. Utilization of general and specialized cardiac care by people with schizophrenia. Psychiatric Services. 2012;63(3):237–42.
25. Chen P, Kales HC, Weintraub D, Blow FC, Jiang L, Ignacio RV, et al. Depression in veterans with Parkinson’s disease: Frequency, co-morbidity, and healthcare utilization. International Journal of Geriatric Psychiatry. 2007;22(6):543–8.
26. Cramer S, Chapa G, Kotsos T, Jenich H. Assessing multiple hospitalizations for health-plan- managed Medicaid diabetic members. Journal for healthcare quality : official publication of the National Association for Healthcare Quality. 2010;32(3):7–14.
27. Davydow DS, Ribe AR, Pedersen HS, Fenger-Gron M, Cerimele JM, Vedsted P, et al. Serious Mental Illness and Risk for Hospitalizations and Rehospitalizations for Ambulatory Care- sensitive Conditions in Denmark A Nationwide Population-based Cohort Study. Med Care. 2016 Jan;54(1):90–7.
28. Hendrie HC, Tu W, Tabbey R, Purnell CE, Ambuehl RJ, Callahan CM. Health outcomes and cost of care among older adults with schizophrenia: A 10-year study using medical records across the continuum of care. American Journal of Geriatric Psychiatry. 2014;22(5):427–36.
29. Hunter G, Yoon J, Blonigen DM, Asch SM, Zulman DM. Health Care Utilization Patterns Among High-Cost VA Patients With Mental Health Conditions. Psychiatr Serv. 2015 Sep;66(9):952–8.
30. Krein SL, Bingham CR, McCarthy JF, Mitchinson A, Payes J, Valenstein M. Diabetes treatment among VA patients with comorbid serious mental illness. Psychiatr Serv. 2006 Jul;57(7):1016– 21.
31. Kurdyak P, Vigod S, Duchen R, Jacob B, Stukel T, Kiran T. Diabetes quality of care and outcomes: Comparison of individuals with and without schizophrenia. General Hospital Psychiatry. 2017;46:7–13.
32. Lafeuille MH, Dean J, Fastenau J, Panish J, Olson W, Markowitz M, et al. Burden of schizophrenia on selected comorbidity costs. Expert Review of Pharmacoeconomics and Outcomes Research. 2014;14(2):259–67.
33. Lemke KW, Weiner JP, Clark JM. Development and validation of a model for predicting inpatient hospitalization. Med Care. 2012 Feb;50(2):131–9.
34. Li Y, Glance LG, Cai X, Mukamel DB. Mental illness and hospitalization for ambulatory care sensitive medical conditions. Medical Care. 2008;46(12):1249–56.
35. Lin H-C, Huang C-C, Chen S-F, Chen Y-H. Increased risk of avoidable hospitalization among patients with schizophrenia. The Canadian Journal of Psychiatry / La Revue canadienne de psychiatrie. 2011;56(3):171–8.
36. Mai Q, Holman CD, Sanfilippo FM, Emery JD. The impact of mental illness on potentially preventable hospitalisations: a population-based cohort study. BMC psychiatry. 2011 Oct 10;11:163.
37. Minen M, Tanev K. Influence of psychiatric comorbidities in migraineurs in the emergency department: A cross sectional analysis. Neurology [Internet]. 2014;82(10). Available from: <http://www.embase.com/search/results?subaction=viewrecord&from=export&id=L71468509>
38. Sayers SL, Hanrahan N, Kutney A, Clarke SP, Reis BF, Riegel B. Psychiatric comorbidity and greater hospitalization risk, longer length of stay, and higher hospitalization costs in older adults with heart failure. Journal of the American Geriatrics Society. 2007 Oct;55(10):1585–91.
39. Schoepf D, Uppal H, Potluri R, Heun R. Physical comorbidity and its relevance on mortality in schizophrenia: A naturalistic 12-year follow-up in general hospital admissions. European Archives of Psychiatry and Clinical Neuroscience. 2014;264(1):3–28.
40. Sullivan G, Han X, Moore S, Kotrla K. Disparities in hospitalization for diabetes among persons with and without co-occurring mental disorders. Psychiatric services (Washington, DC). 2006 Aug;57(8):1126–31.
41. Bailey EA, Wirtalla C, Sharoky CE, Kelz RR. Disparities in operative outcomes in patients with comorbid mental illness. Surgery (United States). 2018;163(4):667–71.
42. Banta JE, Andersen RM, Young AS, Kominski G, Cunningham WE. Psychiatric Comorbidity and Mortality Among Veterans Hospitalized for Congestive Heart Failure. Milit Med. 2010 Oct;175(10):732–41.
43. Beydoun MA, Beydoun HA, Gamaldo AA, Rostant OS, Dore GA, Zonderman AB, et al. Nationwide Inpatient Prevalence, Predictors, and Outcomes of Alzheimer’s Disease among

Older Adults in the United States, 2002-2012. Journal of Alzheimer’s Disease. 2015;48(2):361– 75.

44. Carter P, Reynolds J, Carter A, Potluri S, Uppal H, Chandran S, et al. The impact of psychiatric comorbidities on the length of hospital stay in patients with heart failure. Int J Cardiol. 2016 Mar 15;207:292–6.
45. Cholankeril G, Hu M, Tanner E, Cholankeril R, Reha J, Somasundar P. Skilled nursing facility placement in hospitalized elderly patients with colon cancer. Ejso. 2016 Nov;42(11):1660–6.
46. Dolp R, Rehou S, McCann MR, Jeschke MG. Contributors to the length-of-stay trajectory in burn-injured patients. Burns. 2018;44(8):2011–7.
47. Falsgraf E, Inaba K, De Roulet A, Johnson M, Benjamin E, Lam L, et al. Outcomes after traumatic injury in patients with preexisting psychiatric illness. Journal of Trauma and Acute Care Surgery. 2017;83(5):882–7.
48. Gholson JJ, Bedard NA, Dowdle SB, Brown TS, Gao YB, Callaghan JJ. Total Joint Arthroplasty in Patients With Schizophrenia: How Much Does It Increase the Risk of Complications? J Arthroplast. 2018 Jul;33(7):2082–6.
49. Kaplan GG, Hubbard J, Panaccione R, Shaheen AAM, Quan H, Nguyen GC, et al. Risk of comorbidities on postoperative outcomes in patients with inflammatory bowel disease. Archives of Surgery. 2011;146(8):959–64.
50. Maeda T, Babazono A, Nishi T, Tamaki K. Influence of psychiatric disorders on surgical outcomes and care resource use in Japan. General Hospital Psychiatry. 2014;36(5):523–7.
51. Menendez ME, Neuhaus V, Bot AGJ, Vrahas MS, Ring D. Do psychiatric comorbidities influence inpatient death, adverse events, and discharge after lower extremity fractures? Clinical Orthopaedics and Related Research. 2013;471(10):3336–48.
52. Protty MB, Lacey A, Smith D, Hannoodee S, Freeman P. Increased morbidity, mortality and length of in-hospital stay for patients with acute coronary syndrome with pre-morbid psychiatric diagnoses. International Journal of Cardiology. 2017;236:5–8.
53. Sams JD, Francis ML, Scaife SL, Robinson BS, Novicoff WM, Saleh KJ. Redefining Revision Total Hip Arthroplasty Based on Hospital Admission Status. Journal of Arthroplasty. 2012;27(5):758– 63.
54. Siddiqui N, Dwyer M, Stankovich J, Peterson G, Greenfield D, Si L, et al. Hospital length of stay variation and comorbidity of mental illness: a retrospective study of five common chronic medical conditions. BMC Health Serv Res. 2018 Jun 27;18(1):498.
55. Tarrier N, Gregg L, Edwards J, Dunn K. The influence of pre-existing psychiatric illness on recovery in burn injury patients: The impact of psychosis and depression. Burns. 2005;31(1):45–9.
56. Uldall KK, Koutsky LA, Bradshaw DH, Krone M. Use of hospital services by AIDS patients with psychiatric illness. Gen Hosp Psych. 1998 Sep;20(5):292–301.
57. Willers C, Lekander I, Sunnerhagen KS, Von Euler M. Pre-stroke psychosis and its association with levels of health, resource utilization and care process after ischemic stroke: A register- based study. European Stroke Journal. 2018;3(1):329–30.
58. Ahmedani BK, Solberg LI, Copeland LA, Fang-Hollingsworth Y, Stewart C, Hu J, et al. Psychiatric comorbidity and 30-day readmissions after hospitalization for heart failure, AMI, and pneumonia. Psychiatric Services. 2015;66(2):134–40.
59. Ali AM, Loeffler MD, Aylin P, Bottle A. Factors associated with 30-day readmission after primary total hip arthroplasty analysis of 514 455 procedures in the UK National Health Service. JAMA Surgery [Internet]. 2017;152(12). Available from: <http://www.embase.com/search/results?subaction=viewrecord&from=export&id=L620071714> <http://dx.doi.org/10.1001/jamasurg.2017.3949>
60. Chwastiak LA, Davydow DS, McKibbin CL, Schur E, Burley M, McDonell MG, et al. The Effect of Serious Mental Illness on the Risk of Rehospitalization Among Patients With Diabetes. Psychosomatics. 2014;55(2):134–43.
61. Clark BJ, Keniston A, Douglas IS, Beresford T, Macht M, Williams A, et al. Healthcare utilization in medical intensive care unit survivors with alcohol withdrawal. Alcoholism: Clinical and Experimental Research. 2013;37(9):1536–43.
62. Dailey EA, Cizik A, Kasten J, Chapman JR, Lee MJ. Risk factors for readmission of orthopaedic surgical patients. Journal of Bone and Joint Surgery - Series A. 2013;95(11):1012–9.
63. Daratha KB, Barbosa-Leiker C, M HB, Short R, Layton ME, McPherson S, et al. Co-occurring mood disorders among hospitalized patients and risk for subsequent medical hospitalization. General Hospital Psychiatry. 2012;34(5):500–5.
64. Feller DJ, Akiyama MJ, Gordon P, Agins BD. Readmissions in HIV-infected inpatients: A large cohort analysis. Journal of Acquired Immune Deficiency Syndromes. 2016;71(4):407–12.
65. Fleming MM, Liu F, Luo J, Zhang Y, Pei KY. Predictors of 30 d readmission following percutaneous cholecystostomy. Journal of Surgical Research. 2019;233:1–7.
66. Jørgensen M, Mainz J, Egstrup K, Johnsen SP. Quality of Care and Outcomes of Heart Failure Among Patients With Schizophrenia in Denmark. The American Journal of Cardiology. 2017 Sep 15;120(6):980–5.
67. Kheir MM, Kheir YNP, Tan TL, Ackerman CT, Rondon AJ, Chen AF. Increased Complications for Schizophrenia and Bipolar Disorder Patients Undergoing Total Joint Arthroplasty. Journal of Arthroplasty. 2018;33(5):1462–6.
68. Lau CSM, Siracuse BL, Chamberlain RS. Readmission after COPD exacerbation scale: Determining 30-day readmission risk for COPD patients. International Journal of COPD. 2017;12:1891–902.
69. Lu MLR, De Venecia TA, Goyal A, Rodriguez Ziccardi M, Kanjanahattakij N, Shah MK, et al. Psychiatric conditions as predictors of rehospitalization among African American patients hospitalized with heart failure. Clinical Cardiology. 2017;40(11):1020–5.
70. Moore BJ, White S, Washington R, Coenen N, Elixhauser A. Identifying Increased Risk of Readmission and In-hospital Mortality Using Hospital Administrative Data: The AHRQ Elixhauser Comorbidity Index. Medical Care. 2017;55(7):698–705.
71. Paxton EW, Inacio MCS, Singh JA, Love R, Bini SA, Namba RS. Are There Modifiable Risk Factors for Hospital Readmission After Total Hip Arthroplasty in a US Healthcare System? Clin Orthop Rel Res. 2015 Nov;473(11):3446–55.
72. Shah R, Haydek C, Mulki R, Qayed E. Incidence and predictors of 30-day readmissions in patients hospitalized with chronic pancreatitis: A nationwide analysis. Pancreatology. 2018;18(4):386–93.
73. Singh G, Zhang W, Kuo YF, Sharma G. Association of Psychological Disorders With 30-Day Readmission Rates in Patients With COPD. Chest. 2016 Apr;149(4):905–15.
74. Pugh MJ, Zeber JE, Copeland LA, Tabares JV, Cramer JA. Psychiatric disease burden profiles among veterans with epilepsy: the association with health services utilization. Psychiatric services (Washington, DC). 2008 Aug;59(8):925–8.
75. Shim RS, Druss BG, Zhang S, Kim G, Oderinde A, Shoyinka S, et al. Emergency department utilization among Medicaid beneficiaries with schizophrenia and diabetes: The consequences of increasing medical complexity. Schizophrenia Research. 2014;152(2–3):490–7.
76. Copeland LA, Zeber JE, Wang CP, Parchman ML, Lawrence VA, Valenstein M, et al. Patterns of primary care and mortality among patients with schizophrenia or diabetes: A cluster analysis approach to the retrospective study of healthcare utilization. BMC Health Services Research [Internet]. 2009;9. Available from: <http://www.embase.com/search/results?subaction=viewrecord&from=export&id=L355136201> <http://dx.doi.org/10.1186/1472-6963-9-127>
77. Kontopantelis E, Olier I, Planner C, Reeves D, Ashcroft DM, Gask L, et al. Primary care consultation rates among people with and without severe mental illness: a UK cohort study using the Clinical Practice Research Datalink. BMJ Open. 2015;5(12):10.
78. Lichstein JC, Domino ME, Beadles CA, Ellis AR, Farley JF, Morrissey JP, et al. Use of medical homes by patients with comorbid physical and severe mental illness. Medical Care. 2014;52(3 SUPPL. 2):S85–91.
79. Parks J, Svendsen D, Singer P, Foti ME. Morbidity and mortality in people with serious mental illness [Internet]. Virginia, US: National Association of State Mental Health Program Directors (NASMHPD) Medical Directors Council; 2006 [cited 2019 Nov 19]. Available from: <http://northernlakescmh.com/wp-content/uploads/2010/03/686.pdf>
80. Peckham E, Brabyn S, Cook L, Tew G, Gilbody S. Smoking cessation in severe mental ill health: what works? an updated systematic review and meta-analysis. BMC Psychiatry [Internet]. 2017 Jul 14 [cited 2019 Nov 19];17. Available from: https://[www.ncbi.nlm.nih.gov/pmc/articles/PMC5513129/](http://www.ncbi.nlm.nih.gov/pmc/articles/PMC5513129/)
81. Galletly CA, Foley DL, Waterreus A, Watts GF, Castle DJ, McGrath JJ, et al. Cardiometabolic risk factors in people with psychotic disorders: the second Australian national survey of psychosis. Aust N Z J Psychiatry. 2012 Aug;46(8):753–61.
82. Temmingh HS, Williams T, Siegfried N, Stein DJ. Risperidone versus other antipsychotics for

people with severe mental illness and co‐occurring substance misuse. Cochrane Database Syst Rev [Internet]. 2018 Jan 22 [cited 2019 Nov 19];2018(1). Available from: https://[www.ncbi.nlm.nih.gov/pmc/articles/PMC6491096/](http://www.ncbi.nlm.nih.gov/pmc/articles/PMC6491096/)

83. Bradshaw T, Mairs H. Obesity and Serious Mental Ill Health: A Critical Review of the Literature. Healthcare (Basel). 2014 Apr 1;2(2):166–82.
84. De Hert M, Cohen D, Bobes J, Cetkovich-Bakmas M, Leucht S, Ndetei DM, et al. Physical illness in patients with severe mental disorders. II. Barriers to care, monitoring and treatment guidelines, plus recommendations at the system and individual level. World Psychiatry. 2011 Jun;10(2):138–51.
85. Moore L, Cisse B, Batomen Kuimi BL, Stelfox HT, Turgeon AF, Lauzier F, et al. Impact of socio- economic status on hospital length of stay following injury: a multicenter cohort study. BMC Health Serv Res [Internet]. 2015 Jul 25 [cited 2019 Nov 19];15. Available from: https://[www.ncbi.nlm.nih.gov/pmc/articles/PMC4513757/](http://www.ncbi.nlm.nih.gov/pmc/articles/PMC4513757/)
86. Finley CR, Chan DS, Garrison S, Korownyk C, Kolber MR, Campbell S, et al. What are the most common conditions in primary care? Can Fam Physician. 2018 Nov;64(11):832–40.
87. NHS England. The Five Year Forward View for Mental Health [Internet]. 2016 [cited 2018 Jun 9]. Available from: https://[www.england.nhs.uk/wp-content/uploads/2016/02/Mental-Health-](http://www.england.nhs.uk/wp-content/uploads/2016/02/Mental-Health-) Taskforce-FYFV-final.pdf

**Table 1. The impact of comorbid severe mental illness on inpatient admissions**

| **Authors** | **Population (n)** | **Comorbid SMI (n)** | **SMI assessment** | **Type of admission** | **Control for**  **comorbidities/medical illness severity** | **Control for other variables** | **Main findings** | **NOS** |
| --- | --- | --- | --- | --- | --- | --- | --- | --- |
| Basta et al. | All women who | Psychosis | Elixhauser | Complicated | Elixhauser | Age, insurance type, | Psychosis was associated with an | 8 |
| 2016(22) | underwent | (n=NR) | Comorbidity | lymphedema inpatient | Comorbidity Index, | tobacco use | increased risk of complicated | Good |
|  | lumpectomy or |  | Index | admission within 2 years | cancer in situ vs |  | lymphedema inpatient admission | quality |
|  | mastectomy with a  concurrent axillary |  |  | of initial surgical  procedure | invasive cancer, type  of surgery |  | within two years of surgery  (OR=2·15, CI^95^=1·51-3·06) |  |
|  | lymph node |  |  |  |  |  |  |  |
|  | procedure for a |  |  |  |  |  |  |  |
|  | diagnosis of breast |  |  |  |  |  |  |  |
|  | cancer |  |  |  |  |  |  |  |
|  | (n=56,075) |  |  |  |  |  |  |  |
| Becker et al. | Al residents of | Bipolar | ICD-9 diagnostic | Number of | Charlson Comorbidity | Age, gender, race | Bipolar disorder was associated | 7 |
| 2010(20) | nursing homes in | disorder | codes from | hospitalisations for | Index, diagnosis of |  | with an increased likelihood of | Good |
|  | Florida | (n=657) | Medicaid | ACSCs over 3-year | dementia, alcohol-use |  | hospitalisation for ACSCs during | quality |
|  | (n=72,251) | Major | inpatient claims | study period | disorder, drug-use disorder |  | the study period (HR=1·79, CI^95^=1·50-2·15, *p*<0·001) |  |
|  |  | psychotic |  |  |  |  |  |  |
|  |  | disorder |  |  |  |  | Major psychotic disorder was |  |
|  |  | (n=10,141) |  |  |  |  | associated with an increased |  |
|  |  |  |  |  |  |  | likelihood of hospitalisation for |  |
|  |  |  |  |  |  |  | ACSCs during the study period  (HR=1·44, CI^95^=1·36-1·52, |  |
|  |  |  |  |  |  |  | *p*<0·001) |  |
| Becker et al. | All residents of | Bipolar | ICD-9 diagnostic | Number of | Charlson Comorbidity | Age, gender, race | In people under 65 years, bipolar | 7 |
| 2012(23) | assisted living | disorder | codes from | hospitalisations for | Index, diagnosis of |  | disorder was not associated with | Good |
|  | facilities in Florida | (<65y: n=215, | Medicaid | ACSCs over 5-year | dementia, alcohol-use |  | an increased likelihood of | quality |
|  | (n=16,208) | ≥65y: n=139) | inpatient claims | study period | disorder, drug-use |  | hospitalisation for ACSCs during |  |
|  | Separate analyses | Major |  |  | disorder |  | the study period (HR=1·15,  CI^95^=0·75-1·76, *p*=0·534)· Major |  |
|  | carried out for those | psychotic |  |  |  |  | psychotic disorder was associated |  |
|  | under (n=8217) and | disorder |  |  |  |  | with a *reduced* likelihood of |  |
|  | those 65 years or  older (n=7991) | (<65y:  n=6953, ≥65y: |  |  |  |  | hospitalisation (HR=0·69,  CI^95^=0·58-0·84, *p*<0·001) |  |
|  |  | n=2132) |  |  |  |  |  |  |
|  |  |  |  |  |  |  | In people 65 years or older, |  |
|  |  |  |  |  |  |  | neither bipolar disorder  (HR=0·87, CI^95^=0·58-1·31, |  |

|  |  |  |  |  |  |  | *p*=0·433) nor major psychotic  disorder (HR=0·93, CI^95^=0·82- |  |
| --- | --- | --- | --- | --- | --- | --- | --- | --- |
|  |  |  |  |  |  |  | 1·05, *p*=0·238) was associated |  |
|  |  |  |  |  |  |  | with an increased likelihood of |  |
|  |  |  |  |  |  |  | hospitalisation for ACSCs |  |
| Bresee et al. | All patients on the | Schizophrenia | ICD-9 and ICD- | Hospitalisation (for any | Diagnosis of coronary | Age, sex, SES, urban | Patients with schizophrenia were | 8 |
| 2012(24) | Administrative | (n=28,755) | 10 diagnostic | reason) over the 2-year | artery disease and/or | vs rural dwelling | more likely to be hospitalised for | Good |
|  | Database of Alberta  Health and Wellness |  | codes from  physician claims | study period; ≥1 yearly  hospitalisation | diabetes |  | any reason over the study period  (OR=5·67, CI^95^=5·50-5·84), and | quality |
|  | (1995-2006) |  | data and hospital |  |  |  | were more likely to have one or |  |
|  | (n=2,310,391) |  | discharge data |  |  |  | more hospitalisations per year (OR=7·88, CI^95^=7·54-8·23) |  |
| Chen et al. | Patients with | Psychosis | Not specified | Risk of medical | No | Age, race, marital | Psychosis was associated with an | 6 |
| 2007(25) | Parkinson’s Disease | (n=4429) |  | hospitalisation over a 12- |  | status | increased risk of medical | Poor |
|  | (n=43,772) |  |  | month period |  |  | hospitalisations over a 12-month | quality* |
|  |  |  |  |  |  |  | period in patients with |  |
|  |  |  |  |  |  |  | Parkinson’s disease (OR=1·75, CI^95^=1·61-1·90, *p*<0·0001) |  |
| Cramer et al. | Patients with diabetes | Psychosis | ICD-9 diagnostic | Number of inpatient | Diabetic acute | Gender | Psychosis was associated with an | 6 |
| 2010(26) | with at least two | (n=NR) | codes from | admissions during the | complications, lower |  | increased risk of inpatient | Fair |
|  | hospitalisations in the |  | Medicaid claims | 12-month study period | extremity disease, |  | admissions over a 12-month | quality |
|  | study period (12 |  | data |  | ischaemic heart |  | period among diabetic patients |  |
|  | months)  (n=695) |  |  |  | disease, congestive  heart failure, stroke, |  | with multiple hospitalisations  (OR=2·15, CI^95^=1·18-3·92) |  |
|  |  |  |  |  | cardiac arrhythmia, |  |  |  |
|  |  |  |  |  | COPD, |  |  |  |
|  |  |  |  |  | drug/alcohol/substance |  |  |  |
|  |  |  |  |  | abuse |  |  |  |
| Davydow et al. | Patients hospitalised | Bipolar | Diagnosis | Hospitalisations for | Charlson Comorbidity | Age, sex, calendar | Patients with SMI (bipolar | 9 |
| 2016(27) | for ACSCs | disorder | recorded in the | ACSCs during the 15- | Index | period, marital status, | disorder and/or schizophrenia) | Good |
|  |  | (n = 25,648) | Danish Civil | year study |  | education level, | were at increased risk of | quality |
|  | (n=5,945,540) |  | Registration |  |  | substance abuse, | hospitalisation for all chronic and |  |
|  |  | Schizophrenia (n = 42,558) | System |  |  | primary care use | acute ACSCs (IRR=1·41, CI^95^=1·37-1·45, *p*<0·001) |  |
|  |  |  |  |  |  |  | When looked at separately, both |  |
|  |  |  |  |  |  |  | patients with schizophrenia (IRR=1·47, CI^95^=1·41-1·54, |  |
|  |  |  |  |  |  |  | *p*<0·001) and bipolar disorder |  |

|  |  |  |  |  |  |  | (IRR=1·33, CI^95^=1·27-1·39,  *p*<0·001) had increased risk for ACSCs hospitalisations also |  |
| --- | --- | --- | --- | --- | --- | --- | --- | --- |
| Hendrie et al. | Any patient receiving | Schizophrenia | ICD-9 diagnostic | Hospital admissions | A diagnosis of arthritis, | Age, sex, race, | In fully adjusted analysis, there | 6 |
| 2014(28) | care at Wishard | (n=757) | codes in | during the 10-year study | coronary artery | smoking | was no difference between | Good |
|  | Health Services who |  | electronic hospital | period | disease, cancer, heart |  | patients with schizophrenia and | quality |
|  | were over or reached |  | records or in any |  | failure, COPD, |  | patients without in terms of |  |
|  | the age of 65y during |  | of the linked |  | dementia, diabetes, |  | admissions to hospital (*p*=0·811) |  |
|  | the study period (10 |  | datasets |  | alcohol abuse, |  |  |  |
|  | years) |  |  |  | hypertension, |  |  |  |
|  | (n=31,588) |  |  |  | hyperthyroid, |  |  |  |
|  |  |  |  |  | hypothyroid, liver |  |  |  |
|  |  |  |  |  | disease, renal disease, |  |  |  |
|  |  |  |  |  | stroke, substance abuse |  |  |  |
| Hsieh et al. | Patients on the | Bipolar | Acute admission | Frequency of non- | No | Age, gender, | Patients with bipolar disorder | 7 |
| 2012(21) | Taiwanese National | disorder | ICD-9 diagnostic | psychiatric |  | urbanisation level of | (*M*=0·37, *SD*=1·11) had a | Poor |
|  | Health Research | (n=4067) | codes | hospitalisations for those |  | the residential area, | significantly higher number of | quality* |
|  | Institute |  |  | with bipolar disorder and |  | monthly income | non-psychiatric hospitalisations |  |
|  | (n=16,268) |  |  | matched controls over 2- |  |  | compared to matched controls |  |
|  |  |  |  | year study period |  |  | (*M*=0·15, *SD* =0·48) over the |  |
|  |  |  |  |  |  |  | study period (*p*<0·0001) |  |
| Hunter et al. | Costliest 5% of | Bipolar | ICD-9 diagnostic | Number of | AHRQ comorbidity | Age, sex, | Having SMI was associated with | 8 |
| 2015(29) | Veterans Association | disorder, | codes and chronic | hospitalisations (includes | measures | race/ethnicity, marital | a higher number of inpatient | Good |
|  | patients | schizophrenia, | condition | psychiatric care) over the |  | status, documented | hospital admissions over the | quality |
|  | (n = 261,515) | other | indicators | 12-month study period |  | homelessness during | study period (*p*<0·001)· Patients |  |
|  |  | psychosis | established by |  |  | year of investigation, | with SMI had on average 2·2 |  |
|  |  | (n=33,119) | AHRQ |  |  | correlation within | admissions, whereas patients with |  |
|  |  |  |  |  |  | facilities | no mental health conditions had |  |
|  |  |  |  |  |  |  | on average 1·7 admissions |  |
| Krein et al. | Patients with diabetes | Bipolar | ICD-9 diagnostic | Number of inpatient | No | Patients matched on | In unadjusted analyses, the | 6 |
| 2006(30) | (n=36,546) | disorder, | codes from | hospital stays over the |  | age | likelihood of having an inpatient | Poor |
|  |  | schizophrenia | National | 12-month study period |  |  | admission was increased for | quality* |
|  |  | (excluding latent | Psychosis Registry |  |  |  | patients with SMI (OR=2·80, CI^95^=2·67-2·94) |  |
|  |  | schizophrenia), |  |  |  |  |  |  |
|  |  | schizoaffective |  |  |  |  |  |  |
|  |  | disorder, other |  |  |  |  |  |  |
|  |  | nonorganic |  |  |  |  |  |  |
|  |  | psychoses, |  |  |  |  |  |  |

|  |  | paranoid states, affective  psychoses |  |  |  |  |  |  |
| --- | --- | --- | --- | --- | --- | --- | --- | --- |
|  |  | (n=18,273) |  |  |  |  |  |  |
| Kurdyak et al. | Patients with diabetes | Schizophrenia | Ontario Health | Number of | Johns Hopkins ACG | Age, sex, rural | Patients with schizophrenia had | 8 |
| 2017(31) | (n=1,131,375) | (n=26,259) | Insurance Plan | hospitalisations for | System, duration of | residence, | an increased risk of | Good |
|  |  |  | records detailing | diabetic complications; | diabetes | neighbourhood | hospitalisation for a diabetic | quality |
|  |  |  | three  schizophrenia- | number of  hospitalisations for any |  | income,  neighbourhood | complication (OR=1·35,  CI^95^=1·28-1·43) and an increased |  |
|  |  |  | related physician | non-mental health reason |  | material deprivation, | risk of hospitalisation for any |  |
|  |  |  | visits in 36 | excluding trauma in the |  | past year service use | non-mental health reason |  |
|  |  |  | months, or a  hospitalisation for | 2-year study period |  |  | excluding trauma (OR=1·85,  CI^95^=1·79-1·92) |  |
|  |  |  | schizophrenia |  |  |  |  |  |
| Lafeuille et al. | Patients with | Schizophrenia | ICD-9 diagnostic | Number of | Patients were matched | Patients were matched | Compared to matched non- | 8 |
| 2014(32) | substance | (n=24,652) | codes – at least | hospitalisations over the | 1:1 based on | 1:1 based on | schizophrenic controls, patients | Good |
|  | dependence/abuse, |  | two primary or | 10-year study period | propensity scores using | propensity scores on | with schizophrenia had | quality |
|  | obesity, diabetes, |  | secondary |  | the Charlson | age, gender, state, and | significantly higher rates of |  |
|  | metabolic syndrome,  hyperlipidemia, |  | schizophrenia  diagnoses |  | Comorbidity Index | year of index  admission | hospitalisations over the study  period (IRR=2·23, CI^95^=2·14- |  |
|  | hypertension, |  | recorded during |  |  |  | 2·33, *p*<0·005) |  |
|  | coronary artery |  | study period |  |  |  |  |  |
|  | disease, congestive |  |  |  |  |  |  |  |
|  | heart failure, HIV, |  |  |  |  |  |  |  |
|  | hepatitis C, or COPD |  |  |  |  |  |  |  |
|  | (n=49,304) |  |  |  |  |  |  |  |
| Lemke et al. | All persons enrolled | Bipolar | John Hopkins | Inpatient hospitalisations | Charlson Comorbidity | Age, sex number of | Patients with bipolar disorder of | 8 |
| 2012(33) | on the IMS Health | disorder | ACG system | over the 2-year study | Index, morbidity | prior hospitalisations, | all ages who had experienced a | Good |
|  | Plan Claims | (approximately |  | period | burden modelled using | emergency department | prior hospitalisation in the base | quality |
|  | Database for at least | n=23,160) |  |  | the John Hopkins ACG | episodes not resulting | year had an increased risk of |  |
|  | 6 months in 2006 |  |  |  | System in which a | in inpatient | inpatient hospitalisation |  |
|  | (n=4,632,226) |  |  |  | person is uniquely  assigned to one of 106 | hospitalisations,  outpatient visits, | compared to those without  (OR=1·15 CI^95^=1·06-1·25)· This |  |
|  |  |  |  |  | groups - this model | markers for dialysis | was the same for patients with |  |
|  |  |  |  |  | included 24 groups and | service, nursing | bipolar disorder aged ≥18y to |  |
|  |  |  |  |  | 98 expanded diagnostic | services, and other | <65y who had no prior |  |
|  |  |  |  |  | clusters | major procedures | hospitalisation in the base year (OR=1·68 CI^95^=1·58-1·79) but |  |
|  |  |  |  |  |  |  | there was no significantly |  |

|  |  |  |  |  |  |  | increased risk for patients with bipolar disorder ≥65y who had no  prior hospitalisation in the base |  |
| --- | --- | --- | --- | --- | --- | --- | --- | --- |
|  |  |  |  |  |  |  | year (OR=1·17 CI^95^=0·91-1·52)· |  |
| Li et al. 2008(34) | Patients with ACSCs | Bipolar | ICD-9 diagnostic | Hospitalisations for | Elixhauser | Age, gender, race, | Patients with bipolar disorder | 7 |
|  | (e.g. asthma, heart | disorder | codes from | ACSCs or ‘marker’ | Comorbidity Index | insurance, HMO, | (OR=1·87, CI^95^=1·53-2·30), | Good |
|  | failure, hypertension) and patients with 'marker' conditions | (n=2032)  Schizophrenia | secondary diagnoses | conditions over the 12- month study period |  | income, tobacco use, admission type | schizophrenia (OR=1·83, CI^95^=1·50-2·24), and other psychoses (OR=2·64, CI^95^=1·78- | quality |
|  | (e.g. appendicitis, | (n=2714) |  |  |  |  | 3·90) all had increased |  |
|  | acute myocardial |  |  |  |  |  | likelihoods of being admitted to |  |
|  | infarction, hip/femur | Other |  |  |  |  | hospital for an ACSC or ‘marker’ |  |
|  | fracture). | psychoses |  |  |  |  | condition within the study period |  |
|  |  | (n=795) |  |  |  |  |  |  |
|  | Conditions listed |  |  |  |  |  |  |  |
|  | extensively in |  |  |  |  |  |  |  |
|  | original paper |  |  |  |  |  |  |  |
|  | (n=155,312) |  |  |  |  |  |  |  |
| Lin et al. 2011(35) | All patients included | Schizophrenia | ICD-9 diagnostic | Number of | No | Age, sex, level of | Patients with schizophrenia had a | 6 |
|  | in the Longitudinal | (n=2503) | codes recorded as | hospitalisations for |  | urbanisation, | 3·26-fold higher risk of ACSC | Poor |
|  | Health Insurance |  | a principal | ambulatory care |  | geographic location, | hospitalisation than age and sex- | quality* |
|  | Database released by  the Taiwan National |  | diagnosis in the  year prior to the | sensitive conditions  (ruptured appendix, |  | monthly income | matched controls (RR=3·26,  CI^95^=3·00-3·54, *p*<0·001)· When |  |
|  | Health Research |  | study period | asthma, cellulitis, |  |  | conditions known to be highly |  |
|  | Institute |  |  | congestive heart failure, |  |  | correlated with schizophrenia |  |
|  | (n=22,527) |  |  | diabetes, gangrene, |  |  | were removed from the analyses |  |
|  |  |  |  | hypokalaemia, |  |  | (asthma, diabetes, hypertension), |  |
|  |  |  |  | immunisable conditions, |  |  | patients with schizophrenia had a |  |
|  |  |  |  | malignant hypertension, |  |  | 2·46-fold higher risk of ACSC |  |
|  |  |  |  | pneumonia, |  |  | hospitalisations compared to the |  |
|  |  |  |  | pyelonephritis, and |  |  | comparison group, after adjusting |  |
|  |  |  |  | perforated or bleeding  ulcer) over the 5-year |  |  | for potential confounders  (RR=2·46, CI^95^=2·12-2·86, |  |
|  |  |  |  | study period |  |  | *p*<0·001)· |  |
| Mai et al. 2011(36) | People on the | Schizophrenia | ICD-9 diagnostic | Number of potentially | Charlson Comorbidity | Age, sex, indigenous | Compared to people with no | 8 |
|  | electoral roll | (approximately | codes from the | preventable | Index | status, level of social | mental health conditions, patients | Good |
|  | (n=433,388) | n=5847) | Mental Health Registry in | hospitalisations (ACSCs: vaccine-preventable e.g. |  | disadvantage, level of residential remoteness, | with schizophrenia (RR=2·25, CI^95^=2·12-2·39) and patients | quality |
|  |  |  |  | flu; chronic e.g. diabetes |  |  | with other psychoses (RR=2·36, |  |

|  | The mental health | Other | Western | complications; acute e.g. |  | year at start of follow- | CI^95^=2·26-2·47) were more likely |  |
| --- | --- | --- | --- | --- | --- | --- | --- | --- |
|  | cohort comprised people on both the electoral roll and the Mental Health Registry in Western Australia | psychoses (approximately n=9327) | Australia. | appendicitis, and adverse drug events) over the 15.5-year study period |  | up | to have a potentially preventable hospitalisation (PPHs)·  The leading cause of excess PPHs in patients with schizophrenia were nutritional deficiencies, adverse drug events, convulsions and epilepsy, congestive heart failure, and influenza and pneumonia |  |
|  |  |  |  |  |  |  | The leading cause of excess PPHs in patients with other psychoses were convulsions and epilepsy, pyelonephritis, adverse drug events, other vaccine- preventable conditions, and gangrene· |  |
| Minen et al. | Patients who visited | Bipolar | ICD-9 diagnostic | Inpatient stays over the | No | No | Patients with bipolar disorder had | 7 |
| 2014(37) | the ED with a primary diagnosis of migraine  (n=2872) | disorder (n=NR) | codes recorded in the Partners Research Patient Data Registry, Massachusetts | 10-year study period |  |  | 1·6 times more inpatient stays than patients with other psychiatric disorders·  No inferential analyses were carried out on individual psychiatric conditions, and no comparison was made between patients with bipolar disorder and those without any psychiatric diagnosis | Poor quality* |
| Sayers et al. | Patients ≥65y with | Bipolar | Identified using | Estimated additional | Elixhauser | Age, sex, race, SES | Patients with bipolar disorder had | 8 |
| 2007(38) | congestive heart | disorder | AHRQ’s Clinical | number of | Comorbidity Index |  | an estimated 0·38 (30%) | Good |
|  | failure (n=21,429) | (n=58)  Psychosis | Classifications software (ICD-9 diagnostic codes) | hospitalisations within the 12-month study period |  |  | additional number of hospitalisations compared to patients with no psychiatric | quality |
|  |  | (n=534) |  |  |  |  | illness (*p*=0·001) |  |
|  |  |  |  |  |  |  | Patients with psychosis had an estimated 0·3 (24%) additional |  |

|  |  |  |  |  |  |  | number of hospitalisations compared to patients with no psychiatric illness (*p*<0·001) |  |
| --- | --- | --- | --- | --- | --- | --- | --- | --- |
| Schoepf et al. | Patients admitted for | Schizophrenia | ICD-10 | Number of hospital | No | No | Patients with schizophrenia had | 7 |
| 2014(39) | medical treatment | (n=1418) | diagnostic codes | admissions over the |  |  | almost twofold higher number of | Poor |
|  | across three hospitals |  |  | 11.5-year study period |  |  | admissions to hospital within the | quality* |
|  | (n=15,598) |  |  | study period |  |  | study period (*M*=11·5, *SD*=0·4) |  |
|  |  |  |  |  |  |  | compared to non-schizophrenic |  |
|  |  |  |  |  |  |  | controls (*M*=6·3, *SD*=0·1) |  |
|  |  |  |  |  |  |  | (*p≤*0·001) |  |
| Sullivan et al. | Patients visiting the | ‘Psychotic’ | ICD-9 diagnostic | Whether or not | Proxies for illness | Age, gender, ethnicity | Patients with ‘psychosis’ were no | 7 |
| 2006(40) | ED with a primary | group which | codes | hospitalisation occurred | severity were time of |  | more likely than patients with no | Good |
|  | diagnosis of diabetes | included |  | at the end of the ED visit | arrival at the ED |  | mental illness to be hospitalised | quality |
|  | (n=4275) | patients with bipolar |  |  | (11pm-7am compared with all other times), |  | at the end of the ED visit (OR=0·77, CI^95^=0·45-1·33) |  |
|  |  | disorder and |  |  | and mode of arrival |  |  |  |
|  |  | schizophrenia |  |  | (ambulance vs other) |  |  |  |
|  |  | (n=136) |  |  |  |  |  |  |

ACG = Adjusted Clinical Groups; ACSC = ambulatory care sensitive condition; AHRQ = Agency for Healthcare Research and Quality; CI = confidence interval; COPD = chronic obstructive pulmonary disorder; ED = emergency department; HIV: human immunodeficiency virus; HMO = health maintenance organisation; HR = hazard ratio; ICD = International Statistical Classification of Diseases and Related Health Problems; IRR = incidence rate ratio; M = mean; NOS = Newcastle-Ottawa Scale; NR = not reported; OR = odds ratio; SD = standard deviation; SES = socioeconomic status; SMI = severe mental illness; RR = risk ratios; PPH = potentially preventable hospitalisations

*If studies failed to adjust for physical comorbidities/illness severity in their analyses, they were deemed to be of poor quality regardless of ‘stars’ recorded using the NOS.

**Table 2. The impact of comorbid severe mental illness on length of hospital stay**

| **Authors** | **Population (n)** | **Comorbid SMI (n)** | **SMI assessment** | **Type of LOS** | **Control for**  **comorbidities/medical illness severity** | **Control for other variables** | **Main findings** | **NOS** |
| --- | --- | --- | --- | --- | --- | --- | --- | --- |
| Bailey et al. | Patients who | Schizophrenia | DSM-IV | Surgical LOS | Elixhauser Comorbidity | Age, sex, race, payer | Compared with patients who did | 8 |
| 2018(41) | underwent one of the | (n=5234) | diagnosis in |  | Index; surgery type; | type (US), median | not have a psychiatric diagnosis, | Good |
|  | four most common |  | patient records |  | laparoscopic surgery; | income quartile, | patients with schizophrenia had | quality |
|  | surgeries |  |  | Likelihood of | emergent or urgent | hospital bed size, | an increased risk of prolonged |  |
|  | (cholecystectomy and common duct  exploration, |  |  | prolonged LOS, i.e. LOS greater than the  75th percentile (OR) | surgery | hospital ownership, hospital teaching  status | length of hospital stay (OR=1·64, CI^95^=1·52-1·78, *p*<0·001) |  |
|  | colorectal resection, |  |  |  |  |  |  |  |
|  | excision and lysis of |  |  |  |  |  |  |  |
|  | peritoneal adhesions, |  |  |  |  |  |  |  |
|  | appendectomy) |  |  |  |  |  |  |  |
|  | (n=579,851) |  |  |  |  |  |  |  |
| Banta et al. | Patients with | Schizophrenia, | ICD-9 diagnostic | LOS for index | No | No | Compared with patients with no | 6 |
| 2010(42) | congestive heart | PTSD, bipolar | codes recorded in | hospitalisation for |  |  | psychiatric comorbidity (*M*=6·9 | Poor |
|  | failure | disorder | patient notes in | heart failure |  |  | days, *SD*=9·4 days), those with | quality* |
|  | (n=15,497) | (n=564) | the 12 months |  |  |  | comorbid SMI only (i·e· no |  |
|  |  |  | prior to | LOS in days (mean, |  |  | comorbid depression or anxiety) |  |
|  |  |  | hospitalisation for | SD) |  |  | (*M*=6·7 days, *SD*=7·3 days) did |  |
|  |  |  | heart failure |  |  |  | not differ significantly in terms |  |
|  |  |  |  |  |  |  | of LOS for their index |  |
|  |  |  |  |  |  |  | hospitalisation for heart failure |  |
|  |  |  |  |  |  |  | (*p*=0·62)** |  |
| Beydoun et al. | Population sample of | Psychoses | AHRQ | Association between | AHRQ comorbidity | Age, sex, race, median | Having comorbid psychosis was | 8 |
| 2015(43) | patients with | (n=6652) | comorbidity | psychosis and | measures | household income, | associated with increased average | Good |
|  | Alzheimer’s disease |  | measure | increased LOS (beta) |  | insurance status, | LOS over the study period (12 | quality |
|  | (n=99,260) |  |  |  |  | admissions day | months) (β=2·05, *SE*=0·15, |  |
|  |  |  |  |  |  | (weekday v weekend), | *p*<0·001) |  |
|  |  |  |  |  |  | bed size of hospital, |  |  |
|  |  |  |  |  |  | ownership of hospital, |  |  |
|  |  |  |  |  |  | location/teaching |  |  |
|  |  |  |  |  |  | status of hospital, |  |  |
|  |  |  |  |  |  | region of the hospital |  |  |
| Bressi et al. | Patients hospitalised | Schizophrenia | ICD-9 diagnostic | LOS in days (mean, | APR-DRG illness | Age, sex, race, payer | Patients with schizophrenia | 7 |
| 2006(19) | for medical | (n=NR) | codes recorded as | no SD provided) | severity algorithm | type, region, discharge | incurred hospital stays on | Good |
|  | conditions |  | secondary |  |  | to long-term care, bed | average 0·86 days longer than | quality |
|  | (n=1,617,710) |  | diagnosis |  |  | size of hospital, |  |  |

|  |  |  |  |  |  | hospital location/teaching status | patients without schizophrenia in the adjusted model, *p*<·0001 |  |
| --- | --- | --- | --- | --- | --- | --- | --- | --- |
| Briskman et al. | Patients hospitalised | Bipolar | Identified from | Duration of | No | Age (matched | The duration of hospitalisation | 6 |
| 2012(18) | in general medicine | disorder, | medical records | hospitalisation (no |  | controls) | was significantly longer for | Poor |
|  | departments | schizophrenia, |  | means provided) |  |  | patients with comorbid bipolar | quality* |
|  | (n=192) | (n=93) |  |  |  |  | disorder and/or schizophrenia |  |
|  |  |  |  |  |  |  | compared to patients with no |  |
|  |  |  |  |  |  |  | psychiatric diagnosis (*p*<0·035)· |  |
|  |  |  |  |  |  |  | No descriptive statistics |  |
|  |  |  |  |  |  |  | provided· |  |
| Carter et al. | Patients with heart | Bipolar | ICD-10 | LOS in days (mean, | No | No | LOS was significantly longer in | 7 |
| 2016(44) | failure | disorder, | diagnostic codes | no SD, mean |  |  | patients with bipolar disorder | Poor |
|  | (n=31,760) | schizophrenia | recorded as | difference, CI) |  |  | (*M*=20·4 days) compared to | quality* |
|  |  | (n=237) | secondary |  |  |  | patients with no psychiatric |  |
|  |  |  | diagnosis |  |  |  | disorder (*M*=11·2 days) (*Mean*  *difference*=8·8 days, CI^95^=3·5- |  |
|  |  |  |  |  |  |  | 14·2 days, *p*<0·001) |  |
|  |  |  |  |  |  |  | There was no difference in terms |  |
|  |  |  |  |  |  |  | of LOS between patients with |  |
|  |  |  |  |  |  |  | schizophrenia (*M*=13 days) and |  |
|  |  |  |  |  |  |  | patients with no psychiatric |  |
|  |  |  |  |  |  |  | disorder (*M*=11·2 days) (*Mean*  *difference*=1·4 days, CI^95^=-2·1- |  |
|  |  |  |  |  |  |  | 4·9) |  |
| Cholankeril et al. | Elderly patients | Psychosis | ICD-9 diagnostic | LOS mean difference | Elixhauser Comorbidity | Age, gender, ethnicity | Patients with psychosis had a | 8 |
| 2016(45) | hospitalised for colon | (n=1340) | codes and HCUP- | in days | Index, surgical covariates |  | longer hospital stay (*Mean* | Good |
|  | cancer-directed |  | NIS comorbidity |  | (post-operative delerium, |  | *difference* = 6·4 days, *p*<0·0001) | quality |
|  | surgery |  | software |  | post-operative fistula or |  |  |  |
|  | (n=98,797) |  |  |  | ileus, indication for |  |  |  |
|  |  |  |  |  | reoperation, post-op |  |  |  |
|  |  |  |  |  | DVT, post-operative |  |  |  |
|  |  |  |  |  | respiratory complications, |  |  |  |
|  |  |  |  |  | intra-abdominal abscess) |  |  |  |
| Dolp et al. | Burn patients with | Schizophrenia | NR | Likelihood of | Inhalation injury, | Age, sex | Having comorbid schizophrenia | 5 |
| 2018(46) | burns over 10% of | (n=NR) |  | exceeding average | %TBSA burn |  | increased the likelihood that a | Fair |
|  | total body surface  area |  |  | LOS (OR) |  |  | patient would exceed the average  LOS (OR=2·93, CI^95^=1·06-8·08) | quality |

|  | (n=583) |  |  |  |  |  |  |  |
| --- | --- | --- | --- | --- | --- | --- | --- | --- |
| Falsgraf et al. | Trauma patients | Bipolar disorder | Inpatient hospital | LOS in days (mean, | No | No | Patients with bipolar disorder | 7 |
| 2017(47) | (n=26,502) | (approximately | records | SD) |  |  | (*M*=10·1 days *SD*=14·7 days, | Poor |
|  |  | n=212) |  |  |  |  | *p*=0·02) and patients with | quality* |
|  |  |  |  |  |  |  | schizophrenia (*M*=14·2 days, |  |
|  |  | Schizophrenia |  |  |  |  | *SD*=21·7 days, *p*<0·001) |  |
|  |  | (approximately |  |  |  |  | experienced significantly longer |  |
|  |  | n=344) |  |  |  |  | LOS than patients with trauma |  |
|  |  |  |  |  |  |  | and no psychiatric comorbidity |  |
|  |  |  |  |  |  |  | (*M*=6·2 days, *SD*=14·4 days) |  |
| Gholson et al. | Patients undergoing | Schizophrenia | ICD-8 diagnostic | LOS in days (mean, | Charlson Comorbidity | Age, sex, smoking, | Patients with schizophrenia | 8 |
| 2018(48) | total joint | (n=953) | codes | SD) | Index | race, use of | (*M*=3·85 days, *SD* = 2·14 days) | Good |
|  | arthroplasty |  |  |  |  | corticosteroids, | had a longer LOS than patients | quality |
|  | (n=505,840) |  |  |  |  | osteonecrosis of the | without schizophrenia (*M*=3·22 |  |
|  |  |  |  |  |  | hip, spasm of muscle, | days, *SD*=1·32 days), *p*<0·001 |  |
|  |  |  |  |  |  | gait abnormality, |  |  |
|  |  |  |  |  |  | contracture of joint, |  |  |
|  |  |  |  |  |  | pelvic region, and |  |  |
|  |  |  |  |  |  | thigh, vitamin D |  |  |
|  |  |  |  |  |  | deficiency, surgery |  |  |
|  |  |  |  |  |  | type |  |  |
| Hendrie et al. | Patients over 65y | Schizophrenia | ICD-9 diagnostic | LOS in days (mean, | No | No | Patients with schizophrenia had | 6 |
| 2014(28) | receiving care at | (n=757) | codes in | SD) over the 10-year |  |  | more total hospital days (*M*=58·9 | Poor |
|  | Wishard Health |  | electronic | study period |  |  | days, *SD*=94·2 days) than | quality* |
|  | Services, US |  | hospital notes |  |  |  | patients without schizophrenia |  |
|  | (n=31,588) |  |  |  |  |  | (*M*=31·1 days, *SD*=44·1 days), |  |
|  |  |  |  |  |  |  | *p*<0·001 |  |
| Hsieh et al. | Patients on the | Bipolar disorder | Acute admission | Inpatient LOS in | No | Age, gender, | Over two years, patients with | 7 |
| 2012(21) | Taiwanese National | (n=4067) | ICD-9 diagnostic | days (mean, SD) over |  | urbanisation level of | bipolar disorder (*regression-* | Poor |
|  | Health Research |  | codes | the 2-year study |  | the residential area, | *adjusted annual M*=1·70 days, | quality* |
|  | Institute (case-control |  |  | period |  | monthly income | *SD*=1·31 days) had longer |  |
|  | design) |  |  |  |  |  | inpatient LOS than matched |  |
|  | (n=16,268) |  |  |  |  |  | controls without bipolar disorder |  |
|  |  |  |  |  |  |  | (*regression-adjusted annual* |  |
|  |  |  |  |  |  |  | *M*=1·51, *SD*=1·75), *p*<0·0001 |  |
| Hunter et al. | Costliest 5% of | Bipolar | ICD-9 diagnostic | Medical-surgical | AHRQ comorbidity | Age, sex, | Patients with SMI had longer | 8 |
| 2015(29) | Veterans Association | disorder, | codes and chronic | LOS in days; long- | measures | race/ethnicity, marital | long-term care LOS (*M*=22·9 | Good |
|  | patients |  | condition | term care LOS |  | status, documented | days) compared to patients with | quality |

|  | (n = 261,515) | schizophrenia, | indicators | (means only) over the |  | homelessness during | no mental health conditions |  |
| --- | --- | --- | --- | --- | --- | --- | --- | --- |
|  |  | other psychosis | established by | 12-month study |  | year of investigation, | (*M*=11·4 days)· *P*<0·001· |  |
|  |  | (n=33,119) | AHRQ | period |  | correlation within |  |  |
|  |  |  |  |  |  | facilities | However, patients with SMI had |  |
|  |  |  |  |  |  |  | shorter medical-surgical LOS |  |
|  |  |  |  |  |  |  | (M=6·4 days) compared to |  |
|  |  |  |  |  |  |  | patients with no mental health |  |
|  |  |  |  |  |  |  | conditions (*M*=8·7 days), |  |
|  |  |  |  |  |  |  | *p*<0·001 |  |
| Kaplan et al. | Patients with IBD | Psychosis | Elixhauser | Association between | Elixhauser Comorbidity | Age, sex, race, | Psychosis was associated with a | 8 |
| 2011(49) | who had undergone | (n=348) | Comorbidity | psychosis and | Index | primary health insurer, | 22% higher LOS (antilogarithm | Good |
|  | IBD-related surgery  (n = 35,588) |  | Index | increased LOS  (antilogarithms of |  | emergency admission | of regression coefficient=1·22,  CI^95^=1·16-1·29) | quality |
|  |  |  |  | regression coefficient |  |  |  |  |
|  |  |  |  | presented to provide |  |  |  |  |
|  |  |  |  | a percentage change |  |  |  |  |
|  |  |  |  | in resource use) |  |  |  |  |
| Krein et al. | Patients with diabetes | Bipolar | ICD-9 diagnostic | LOS in days (mean, | No | Patients matched on | Patients with SMI had longer | 6 |
| 2006(30) | (n=36,546) | disorder, | codes from | SD) over the 12- |  | age | mean LOS (*M*=12·0 days, | Poor |
|  |  | schizophrenia | National | month study period |  |  | *SD*=15·9 days) than patients | quality* |
|  |  | (excluding | Psychosis |  |  |  | without SMI (*M*=8·2 days, |  |
|  |  | latent | Registry |  |  |  | *SD*=11·4 days) |  |
|  |  | schizophrenia), |  |  |  |  |  |  |
|  |  | schizoaffective |  |  |  |  |  |  |
|  |  | disorder, other |  |  |  |  |  |  |
|  |  | nonorganic |  |  |  |  |  |  |
|  |  | psychoses, |  |  |  |  |  |  |
|  |  | paranoid states, |  |  |  |  |  |  |
|  |  | affective |  |  |  |  |  |  |
|  |  | psychoses |  |  |  |  |  |  |
|  |  | (n=18,273) |  |  |  |  |  |  |
| Lafeuille et al. | Patients with | Schizophrenia | ICD-9 diagnostic | Likelihood of | Patients were matched 1:1 | Patients were matched | Patients with schizophrenia had a | 8 |
| 2014(32) | substance | (n=24,652) | codes – at least | prolonged LOS (not | based on propensity | 1:1 based on | significantly higher risk of | Good |
|  | dependence/abuse, |  | two primary or | defined) (IRR) | scores using the Charlson | propensity scores on | prolonged LOS compared to non- | quality |
|  | obesity, diabetes,  metabolic syndrome, |  | secondary  schizophrenia |  | Comorbidity Index | age, gender, state, and  year of index | schizophrenic matched controls  (IRR=2·17, CI^95^=1·90-2·47, |  |
|  | hyperlipidemia, |  | diagnoses |  |  | admission | *p*<0·005) |  |
|  | hypertension, |  | recorded during |  |  |  |  |  |
|  | coronary artery |  | study period |  |  |  |  |  |
|  | disease, congestive |  |  |  |  |  |  |  |

|  | heart failure, HIV,  hepatitis C, or COPD |  |  |  |  |  |  |  |
| --- | --- | --- | --- | --- | --- | --- | --- | --- |
|  | (n=49,304) |  |  |  |  |  |  |  |
| Maeda et al. | Patients undergoing | Schizophrenia | ICD-10 | Association between | Charlson Comorbidity | Age, sex, surgery | Having schizophrenia was | 7 |
| 2014(50) | major surgery | (n=104) | diagnostic codes | psychosis and | Index, delirium, | type, ambulance use, | associated with an increased LOS | Good |
|  | (cardiovascular, |  | from medical | increased LOS (beta) | intubation, haemodialysis | data year on clinical | (β=0·48, CI^95^=0·32-0·64, | quality |
|  | intrathoracic, |  | records |  | related to surgery | outcomes | *p*<0·001) |  |
|  | intraperitoneal, and |  |  |  |  |  |  |  |
|  | suprainguinal- |  |  |  |  |  |  |  |
|  | vascular procedures |  |  |  |  |  |  |  |
|  | requiring general |  |  |  |  |  |  |  |
|  | anaesthesia, |  |  |  |  |  |  |  |
|  | excluding |  |  |  |  |  |  |  |
|  | percutaneous |  |  |  |  |  |  |  |
|  | procedures and |  |  |  |  |  |  |  |
|  | obstetric surgery) |  |  |  |  |  |  |  |
|  | (n=5569) |  |  |  |  |  |  |  |
| Menendez et al. | Patients with a lower | Schizophrenia | ICD-9 diagnostic | LOS in days (mean, | No | No | Patients with schizophrenia had a | 7 |
| 2013(51) | extremity fracture | (approximately | codes recorded in | SD) |  |  | longer LOS (*M*=11·0 days, | Poor |
|  | (n=10,669,449) | n=64,000) | National Hospital |  |  |  | *SD*=21·0 days) compared to | quality* |
|  |  |  | Discharge Survey |  |  |  | patients with no psychiatric |  |
|  |  |  | database |  |  |  | diagnosis (*M*=7·2 days, *SD*=8·3 |  |
|  |  |  |  |  |  |  | days), *p*<0·001 |  |
| Protty et al. | Patients who had | Schizophrenia | ICD-10 | LOS in days (mean, | No | No | Patients with schizophrenia | 7 |
| 2017(52) | experienced their | (n=236) | diagnostic codes | CI, mean difference) |  |  | (*M*=15·67 days, CI^95^=11·93- | Poor |
|  | first acute coronary |  | from electronic |  |  |  | 19·42 days) had a longer LOS | quality* |
|  | syndrome |  | patient records of |  |  |  | compared to patients with no |  |
|  | (n=57,668) |  | previous hospital admissions |  |  |  | psychiatric diagnosis (*M*=9·78 days, CI^95^=9·66-9·91), *p*<0·001 |  |
| Sams et al. | Patients undergoing | Psychosis | Charlson and | LOS in days (means, | No | No | Patients with psychosis had | 7 |
| 2012(53) | total hip arthroplasty | (n=NR) | Elixhauser | medians, no SD) |  |  | longer elective LOS (*M*=5·1 | Poor |
|  | (n=23,444) |  | Comorbidity |  |  |  | days) compared to the entire | quality* |
|  |  |  | Indices |  |  |  | sample (*M*=4·4 days) |  |
|  |  |  |  |  |  |  | Patients with psychosis had |  |
|  |  |  |  |  |  |  | longer non-elective LOS |  |
|  |  |  |  |  |  |  | (*M*=9·32 days) compared to the |  |
|  |  |  |  |  |  |  | entire sample (*M*=7·24 days), |  |
|  |  |  |  |  |  |  | *p*<0·0001 |  |

| Sayers et al. | Patients ≥65y with | Bipolar disorder | Identified using | Estimated additional | Elixhauser Comorbidity | Age, sex, race, SES | Patients with bipolar disorder had | 8 |
| --- | --- | --- | --- | --- | --- | --- | --- | --- |
| 2007(38) | congestive heart | (n=58) | AHRQ’s Clinical | mean LOS in days | Index |  | an estimated additional mean | Good |
|  | failure |  | Classifications | over 12-month study |  |  | LOS of 1·43 days (32%, *p*=0·02) | quality |
|  | (n=21,429) | Psychosis | software (ICD-9 | period |  |  | and patients with psychosis had |  |
|  |  | (n=534) | diagnostic codes) |  |  |  | an estimated additional mean of |  |
|  |  |  |  |  |  |  | 1·06 days (24%, *p*<0·001) |  |
|  |  |  |  |  |  |  | compared to patients with no |  |
|  |  |  |  |  |  |  | psychiatric comorbidity |  |
| Schoepf et al. | Patients admitted for | Schizophrenia | ICD-10 | LOS in days (mean, | No | No | Patients with schizophrenia | 7 |
| 2014(39) | medical treatment | (n=1418) | diagnostic codes | SD) |  |  | (*M*=8·1 days, *SD*=0·6 days) had | Poor |
|  | across three hospitals |  |  |  |  |  | a longer average LOS at index | quality* |
|  | (n=15,598) |  |  |  |  |  | hospitalisation compared to non- |  |
|  |  |  |  |  |  |  | schizophrenic controls (*M*=3·4 |  |
|  |  |  |  |  |  |  | days, *SD*=1·0 days), *p*<0·001 |  |
| Siddiqui et al. | Patients admitted | Schizophrenia | ICD-10 | LOS in days (mean, | Charlson Comorbidity | Age, sex, SES, | The average LOS was longer for | 8 |
| 2018(54) | with one of five | (n=34) | diagnostic codes | SD) over the 5-year | Index | financial year, primary | patients with schizophrenia | Good |
|  | chronic medical |  | entered as | study period |  | physical diagnosis | (*M*=9·9 days, *SD*=8·7 days) | quality |
|  | conditions: cancer |  | secondary | (obtained directly |  |  | compared to patients without |  |
|  | (lung, colorectal), |  | diagnosis during | from author) |  |  | (*M*=5·5 days, *SD*=8·7 days)† |  |
|  | COPD, Type 2 |  | the course of the |  |  |  |  |  |
|  | diabetes, ischaemic |  | hospital |  |  |  | Using fully adjusted negative |  |
|  | heart disease, or |  | admission |  |  |  | binomial regression, |  |
|  | stroke |  |  |  |  |  | schizophrenia was associated |  |
|  | (n=16,898) |  |  |  |  |  | with a 91·2% longer LOS (CI^95^=39·3%-162·6%)† |  |
| Tarrier et al. | Burn injury patients | Psychosis | ICD-10 | LOS in days (mean, | No | No | Patients with psychosis had a | 6 |
| 2005(55) | admitted to an | (n=9) | diagnostic codes | SD) |  |  | longer LOS (*M*=40·4 days, | Poor |
|  | inpatient burns unit |  | recorded in |  |  |  | *SD*=37·1 days) compared to non- | quality* |
|  | (n=27) |  | hospital’s patient |  |  |  | psychotic controls (*M*=13·0 days, |  |
|  |  |  | administration |  |  |  | *SD*=18·9 days), *p*=0·004 |  |
|  |  |  | system |  |  |  |  |  |
| Uldall et al. | AIDS patients | Bipolar | ICD-9 diagnostic | LOS in days | No | No | The median LOS for patients | 7 |
| 1998(56) | admitted to hospital | disorder, | codes in hospital | (median) over the 3- |  |  | with schizophrenia (22 days), | Poor |
|  | for medical-surgical | schizophrenia, | records | year study period |  |  | psychosis (10 days), and bipolar | quality* |
|  | reasons | psychosis |  |  |  |  | disorder (9 days) was higher than |  |
|  | (n=1295) | (n=NR) |  |  |  |  | the median stay for those with no |  |
|  |  |  |  |  |  |  | psychiatric illness (7 days)· |  |

|  |  |  |  |  |  |  | However, this difference was not statistically significant |  |
| --- | --- | --- | --- | --- | --- | --- | --- | --- |
| Willers et al. | Patients with | Schizophrenia, | The presence of | Association between | Diagnosis of atrial | Age, sex, marital | There was no association | 9 |
| 2018(57) | ischaemic stroke | psychosis | at least one care | psychosis and | fibrillation and/or | status, born outside | between psychosis and increased | Good |
|  | (n=46,350) | (n=389) | event related to  psychosis or | increased LOS (beta) | hypertension, ADL-  dependency, prior stroke, | EU, living alone,  living arrangements | LOS in patients with ischaemic  stroke (β=0·06, CI^95^=-0·06-0·19) | quality |
|  |  |  | schizophrenia | LOS in days (mean, | inpatient care prior to |  |  |  |
|  |  |  | according to ICD-  10 diagnostic | CI) over the 4-year  study period | stroke, unconscious at  arrival, NIHSS score at |  | (Comorbid psychosis LOS:  *M*=25·1 days, CI^95^=21·1-29·2 |  |
|  |  |  | codes |  | arrival |  | days, no psychosis LOS: *M*=20·5  days, CI^95^=20·2-20·8 days) |  |

ADL = Activities of Daily Living; AHRQ = Agency for Healthcare Research and Quality; AIDS = Acquired Immune Deficiency Syndrome; APR-DRG = All-Patient Refined Diagnostic Related Groups; CI =

confidence interval; COPD = chronic obstructive pulmonary disorder; DSM-IV = Diagnostic and Statistical Manual of Mental Disorders-IV; DVT = deep vein thrombosis; EU = European Union; HCUP-NIS = Healthcare Cost and Utilization Project – National Inpatient Sample; HIV = Human Immunodeficiency Virus; IBD = inflammatory bowel disease; ICD = International Statistical Classification of Diseases and Related Health Problems; IRR = incident rate ratio; LOS = length of stay; M = mean; NIHSS = National Institute for Health Stroke Scale; NOS = Newcastle-Ottawa Scale; NR = not reported; OR = odds ratio; PTSD; post-traumatic stress disorder; SD = standard deviation; SE = standard error; SES = socioeconomic status; SMI = severe mental illness; TBSA = total burn surface area; US = United States

*If studies failed to adjust for physical comorbidities/illness severity in their analyses, they were deemed to be of poor quality regardless of ‘stars’ recorded using the NOS

**Independent t-test carried out by author AR using data provided in paper

†Raw data obtained through personal correspondence with authors

**Table 3. The impact of comorbid severe mental illness on hospital readmissions**

| **Authors** | **Population (n)** | **Comorbid SMI (n)** | **SMI assessment** | **Type of readmission** | **Control for**  **comorbidities/medical illness severity** | **Control for other variables** | **Main findings** | **NOS** |
| --- | --- | --- | --- | --- | --- | --- | --- | --- |
| Ahmedani et al. | Patients hospitalised | Bipolar | ICD-9 diagnostic | 30-day hospital | No | No | 30-day readmission rates for | 7 |
| 2015(58) | for heart failure, | disorder | codes for SMI | readmission (all- |  |  | bipolar disorder (21·2%, | Poor |
|  | acute myocardial | (n = 696) | recorded at least | cause) |  |  | *p*<0·001), schizophrenia (21·1%, | quality* |
|  | infarction, or |  | twice in any |  |  |  | *p*<0·05) and other psychoses |  |
|  | pneumonia | Schizophrenia | secondary care |  |  |  | (30·4%, *p*<0·001) were |  |
|  | (n=89,406) | (n = 257) | setting in the 12 |  |  |  | significantly higher than rates for |  |
|  |  |  | months prior to |  |  |  | patients with no psychiatric |  |
|  |  | Other | hospitalisation |  |  |  | diagnosis (16·5%) |  |
|  |  | psychoses |  |  |  |  |  |  |
|  |  | (n = 502) |  |  |  |  |  |  |
| Ali et al. 2017(59) | Patients admitted for | Psychosis | ICD-10 | 30-day hospital | 30+ comorbid conditions | Sex, age categories, | Psychosis was significantly | 8 |
|  | total hip arthroplasty | (n = 960) | diagnostic codes | readmission (all- | included in analysis, type | SES, ethnicity, index | associated with increased risk of | Good |
|  | (n=514,455) |  |  | cause, surgical,  return-to-theatre) | of surgical procedure,  return to theatre during | LOS, number of prior  emergency admissions | all-cause 30-day readmission  (OR=1·51, CI^95^=1·23-1·86, | quality |
|  |  |  |  |  | index admission |  | *p*<0·001), surgical 30-day |  |
|  |  |  |  |  |  |  | readmission (OR=1·73,  CI^95^=1·33-2·25, *p*<0·001), and |  |
|  |  |  |  |  |  |  | return-to-theatre 30-day |  |
|  |  |  |  |  |  |  | readmissions (OR=1·83, CI^95^=1·16-2·87, *p*=0·009) |  |
| Chwastiak et al. | Patients with diabetes | Bipolar | ICD-9 diagnostic | 30-day hospital | Elixhauser Comorbidity | Age, gender, payer | Having SMI was associated with | 8 |
| 2014(60) | who had a medical- | disorder, | codes | readmission, | Index | type (US), number of | an increased risk of 30-day | Good |
|  | surgical  hospitalisation | schizophrenia,  psychotic |  | readmission  throughout study |  | hospitalisations in the  12 months prior, was | hospital readmission (OR=1·24,  CI^95^=1·07-1·44, *p*=0·006)· | quality |
|  | (n=80,907) | disorders, |  | period (24 months) |  | the index |  |  |
|  |  | delusional |  | (for medical-surgical |  | hospitalisation through | Having SMI was also associated |  |
|  |  | disorders, |  | illness) |  | the ED?, LOS | with a 14% greater risk of |  |
|  |  | nonorganic |  |  |  |  | readmission throughout the study |  |
|  |  | psychoses (n = 1820) |  |  |  |  | period (24 months) (HR= 1·14, CI^95^=1·05-1·23, *p*=0·002) |  |
| Clark et al. | Patients with alcohol | Bipolar | ICD-9 diagnostic | Likelihood of having | Charlson Comorbidity | Age, homelessness | Bipolar disorder was associated | 7 |
| 2013(61) | withdrawal admitted | disorder | codes for SMI | ≥3 hospital | Index; UHC severity of |  | with a higher risk of having 3 or | Good |
|  | to medical ICU** | (n = 121) | recorded twice in | readmissions in the | illness quartiles; diagnosis |  | more readmissions to hospital in | quality |
|  | (n=1178) | Schizophrenia | the preceding  three years | 12 months following  discharge (all-cause) | of depression and anxiety |  | the year following initial  discharge (OR=1·93, CI^95^=1·08- |  |
|  |  | (n = 50) |  |  |  |  | 3·46, *p*=0·027)· Schizophrenia |  |

|  |  |  |  |  |  |  | was not (OR=1·61, CI^95^=0·71- 3·63, *p*=0·252) |  |
| --- | --- | --- | --- | --- | --- | --- | --- | --- |
| Dailey et al. | Patients admitted for | Psychosis | Elixhauser | 30-day hospital | No | No | Psychosis was associated | 6 |
| 2013(62) | orthopaedic surgical | (n = 53) | comorbidity index | readmission (all- |  |  | (unadjusted analysis) with an | Poor |
|  | procedures |  |  | cause) |  |  | increased risk of 30-day hospital | quality* |
|  | (n=3261) |  |  |  |  |  | readmission (OR=3·58, CI^95^=1·59-8·08, *p*=0·001) |  |
| Daratha et al. | Patients admitted to | Bipolar | ICD-9 diagnostic | Likelihood of | Elixhauser Comorbidity | Age, gender, index | Patients with bipolar disorder | 7 |
| 2012(63) | hospital for medical | disorder | codes recorded as | subsequent medical | Index, substance | hospitalisation primary | were at an increased risk of | Good |
|  | illness | (n=9019) | secondary | hospitalisations | disorders, and interactions | diagnosis, LOS, index | further inpatient admissions | quality |
|  | (n=925,705) |  | diagnoses on | following index | between substance | hospitalisation via ED, | compared to those without during |  |
|  |  | Schizophrenia | index admission | hospitalisation over | disorders and SMI | primary payer, 12- | the study period (HR=1·13, |  |
|  |  | (n=6868) | to hospital | the follow-up period |  | month count of | CI99=1·08-1·17, *p*<0·001) |  |
|  |  |  |  | (ranged from one to |  | previous |  |  |
|  |  |  |  | 72 months post- |  | hospitalisations | However, patients with comorbid |  |
|  |  |  |  | surgery) |  |  | schizophrenia were no more |  |
|  |  |  |  |  |  |  | likely than those without to return |  |
|  |  |  |  |  |  |  | for inpatient medical (HR=1·00, |  |
|  |  |  |  |  |  |  | CI99=0·95-1·05, *p*=0·92) |  |
| Davydow et al. | Patients hospitalised | Bipolar | Diagnosis | 30-day hospital | Charlson Comorbidity | Age, sex, calendar | Bipolar disorder and | 9 |
| 2016(27) | for ACSCs | disorder | recorded in the | readmission (for | Index | period, marital status, | schizophrenia were examined | Good |
|  | (n=5,945,540) | (n = 25,648) | Danish Civil | same ACSC or |  | education level, | together· SMI was associated | quality |
|  |  |  | Registration | different ACSC) |  | substance abuse, | with an increased risk of 30-day |  |
|  |  | Schizophrenia  (n = 42,558) | System |  |  | primary care use | readmission for the same ACSC  (IRR=1·13, CI^95^=1·04-1·23, |  |
|  |  |  |  |  |  |  | p<0·001) and 30-day readmission |  |
|  |  |  |  |  |  |  | for a different ACSC (IRR=1·30, CI^95^=1·20-1·41, p<0·001) |  |
| Feller et al. | Patients admitted to | Psychosis | ICD-9 codes | 30-day hospital | Elixhauser Comorbidity | Sex, SES, race, | Psychosis was associated with an | 8 |
| 2016(64) | general medical | (n = 1872) | present on patient | readmission | Index; diagnosis of | location, ever left | increased risk of 30-day hospital | Good |
|  | hospital with HIV  (n=16,558) |  | discharge | (medical-surgical) | depression; substance  abuse | hospital against  medical advice, | readmission (OR=1·43,  CI^95^=1·27-1·62, *p*<0·01) | quality |
|  |  |  |  |  |  | inpatient admissions, |  |  |
|  |  |  |  |  |  | ED admissions, access |  |  |
|  |  |  |  |  |  | to stable housing |  |  |
| Fleming et al. | Patients admitted for | Psychosis | ICD-9 diagnostic | 30-day hospital | No | No | 2·72% (n=19) of patients who | 7 |
| 2018(65) | percutaneous | (n = 118) | codes | readmission (all- |  |  | were readmitted within 30 days | Poor |
|  | cholecystostomy |  |  | cause) |  |  | were psychotic compared with | quality* |

|  | (n=3368) |  |  |  |  |  | 3·71% (n=99) of patients who were not readmitted· There was no significant difference between these groups (*p*=0·21) |  |
| --- | --- | --- | --- | --- | --- | --- | --- | --- |
| Jorgensen et al. | Patients admitted | Schizophrenia | Identified from | 28-day hospital | Previous myocardial | Age, sex, alcohol | Patients with schizophrenia did | 8 |
| 2017(66) | with heart failure | (n = 108) | the Danish | readmission (all non- | infarction, stroke, COPD, | intake, smoking habits | not have a higher risk of 28-day | Good |
|  | (n=36,718) |  | Schizophrenia Registry | psychiatric) | hypertension, diabetes; left ventricular ejection |  | hospital readmission (OR=1·77, CI^95^=0·79-3·92) | quality |
|  |  |  |  |  | fraction (measure of heart |  |  |  |
|  |  |  |  |  | failure severity) |  |  |  |
| Kheir et al. | Patients admitted for | Bipolar | ICD-9 diagnostic | 90-day, 12-month, | Charlson Comorbidity | Age, gender, BMI, | Patients with SMI were more | 7 |
| 2018(67) | total joint | disorder, | codes | and 24-month aseptic | Index | joint type, LOS, | likely to experience aseptic | Good |
|  | arthroplasty  (n=579) | schizophrenia  (n = 156) |  | hospital readmission  (related to index |  | operative time,  primary vs revision | hospital readmission at 90 days  (OR=2·92, CI^95^=1·45-5·88, | quality |
|  |  |  |  | surgery) |  | surgery | *p*=0·003), one year (OR=2·24, CI^95^=1·39-3·60, *p*=0·001), and  two years (OR=1·83, CI^95^=1·18- |  |
|  |  |  |  |  |  |  | 2·86, *p*=0·008) |  |
| Lau et al. 2017(68) | Patients admitted to | Psychosis | Extracted from | 30-day hospital | Alcohol abuse, anaemia, | Age, gender, race, | Derivation cohort: Psychosis was | 6 |
|  | hospital with COPD | (derivation | the HCUP SID | readmission (related | congestive heart failure, | income, payer (US) | associated with an increased risk | Good |
|  | (derivation cohort: n=339,389; | cohort: n= 22,228; |  | to COPD) | depression, diabetes, drug abuse, liver disease, solid |  | of 30-day hospital readmission (OR=1·19, CI^95^=1·13-1·25, | quality |
|  | validation cohort: | validation |  |  | tumour without |  | *p*<0·01) |  |
|  | n=258,113) | cohort: |  |  | metastases |  |  |  |
|  |  | n=13,745) |  |  |  |  | Validation cohort: Psychosis was |  |
|  |  |  |  |  |  |  | associated with an increased risk |  |
|  |  |  |  |  |  |  | of 30-day hospital readmission  (OR=1·16, CI^95^=1·08-1·24, |  |
|  |  |  |  |  |  |  | *p*<0·01) |  |
| Lu et al. 2017(69) | Patients admitted to | Bipolar | Electronic | 30-day hospital | Hypertension, diabetes, | Age, sex, living | Patients with schizophrenia | 8 |
|  | hospital with | disorder | medical records – | readmission related to | chronic kidney disease, | situation, marital | (OR=4·92, CI^95^=2·49-9·71, | Good |
|  | decompensated heart | (n = 11) | diagnostic codes | heart failure; any | coronary artery disease; | status | *p*<0·001) and patients with | quality |
|  | failure (n=611) | Schizophrenia | not specified | hospital readmission over 3-year study | heart failure severity was measured using peak |  | bipolar disorder (OR=3·44, CI^95^=1·19-10·00, *p*=0·02) had |  |
|  |  | (n = 40) |  | period (all-cause) | troponin I, left ventricular |  | increased risk of 30-day hospital |  |
|  |  |  |  |  | ejection fraction, B-type |  | readmission |  |
|  |  |  |  |  | natriuretic peptide |  |  |  |
|  |  |  |  |  |  |  | Patients with schizophrenia (2·33,  CI^95^=1·51-3·61, *p*<0·001) and |  |

|  |  |  |  |  |  |  | patients with bipolar disorder (OR=2·08, CI^95^=1·05-4·11, |  |
| --- | --- | --- | --- | --- | --- | --- | --- | --- |
|  |  |  |  |  |  |  | *p*=0·03) also had increased risk |  |
|  |  |  |  |  |  |  | for any hospital readmission over |  |
|  |  |  |  |  |  |  | the study period |  |
| Moore et al. | All nonmaternal | Psychosis | ICD-9 codes | 30-day hospital | Elixhauser Comorbidity | No | Psychosis was associated with | 8 |
| 2017(70) | inpatients over a one- | (n = 505,575) | listed on | readmission (all- | Index |  | increased risk of 30-day hospital | Good |
|  | year period in 18 US states |  | discharge record | cause) |  |  | readmission (OR=1·34, CI^95^=1·33-1·35, no *p* value | quality |
|  | (n=10,777,210) |  |  |  |  |  | reported) |  |
| Paxton et al. | Patients admitted for | Psychosis | Elixhauser | 30-day hospital | Elixhauser Comorbidity | Age, sex, race, BMI, , | Psychosis was associated with | 8 |
| 2015(71) | total hip arthroplasty | (n = 755) | Comorbidity | readmission (related | Index; ASA physical | discharge disposition, | increased risk of 30-day hospital | Good |
|  | (n=12,030) |  | Index | to index surgery) | status score; in-hospital  medical complications; | LOS, whether surgeon  had total joint | readmission (OR=1·32,  CI^95^=1·03-1·69, *p*=0·028) | quality |
|  |  |  |  |  | in-hospital surgical | arthroplasty |  |  |
|  |  |  |  |  | complications | fellowship, surgeon’s |  |  |
|  |  |  |  |  |  | average yearly volume |  |  |
|  |  |  |  |  |  | of procedures |  |  |
|  |  |  |  |  |  | performed, hospital |  |  |
|  |  |  |  |  |  | volume |  |  |
| Shah et al. | Patients hospitalised | Psychosis | Elixhauser | 30-day hospital | Elixhauser Comorbidity | Age, gender, | Psychosis was associated with | 8 |
| 2018(72) | with chronic | (n = 1921) | Comorbidity | readmission (all- | Index; acute pancreatitis, | household income, | increased risk of 30-day hospital | Good |
|  | pancreatitis (n=25,259) |  | Index | cause) | pseudocyst, benign pancreatic neoplasms, | alcohol abuse, smoking, primary | readmission (HR=1·12, CI^95^=1·03-1·23, *p*=0·007) | quality |
|  |  |  |  |  | cholangitis, pancreatic | payer information, |  |  |
|  |  |  |  |  | surgery, endoscopic | weekend vs weekday |  |  |
|  |  |  |  |  | retrograde | admission, LOS, |  |  |
|  |  |  |  |  | cholangiopancreatography | discharge disposition, |  |  |
|  |  |  |  |  |  | hospital ownership |  |  |
|  |  |  |  |  |  | status, bed size, |  |  |
|  |  |  |  |  |  | metropolitan status |  |  |
| Singh et al. | Older adults (66+) | Psychosis | Elixhauser | 30-day hospital | Use of mechanical | Age, sex, region, year | Psychosis was associated with | 8 |
| 2016(73) | hospitalised for | (n = 4511) | Comorbidity | readmission (all- | ventilator, ICU admission | of discharge, discharge | increased risk of 30-day hospital | Good |
|  | COPD (n=135,498) |  | Index | cause) | during hospitalisation; comorbid psychological | destination, race, SES, LOS | readmission (OR=1·18, CI^95^=1·10-1·27) | quality |
|  |  |  |  |  | disorders |  |  |  |

ACSC = ambulatory care sensitive condition; ASA = American Society of Anesthesiologists; BMI = body mass index; CI = confidence interval; COPD = chronic pulmonary obstructive disorder; ED = emergency department; HCUP SID = Healthcare Cost and Utilization Project Stat Inpatient Database; HIV = human immunodeficiency virus; HR = hazard ratio; ICD = International Statistical Classification of Diseases and

Related Health Problems; ICU = intensive care unit; IRR = incident rate ratio; LOS = length of stay; NOS = Newcastle-Ottawa Scale; OR = odds ratio; SES = socioeconomic status; SMI = severe mental illness; TJA

= total joint arthroplasty; UHC = University Health System Consortium; US = United States

*If studies failed to adjust for physical comorbidities/illness severity in their analyses, they were deemed to be of poor quality regardless of ‘stars’ recorded using the NOS.

**77% of index admissions were for medical illnesses other than alcohol withdrawal; therefore, this study was included in the review

**Table 4. The impact of comorbid severe mental illness on emergency department use**

| **Authors** | **Population (n)** | **Comorbid SMI (n)** | **SMI assessment** | **Type of ED use** | **Control for**  **comorbidities/medical illness severity** | **Control for other variables** | **Main findings** | **NOS** |
| --- | --- | --- | --- | --- | --- | --- | --- | --- |
| Bresee et al. | All patients on the | Schizophrenia | ICD-9 and ICD- | ED visit over the 2- | Diagnosis of coronary | Age, sex, SES, urban | Patients with schizophrenia were | 8 |
| 2012(24) | Administrative | (n=28,755) | 10 diagnostic | year study period; ≥1 | artery disease and/or | vs rural dwelling | more likely to visit the ED over | Good |
|  | Database of Alberta Health and Wellness |  | codes from physician claims | yearly ED visit | diabetes |  | the 2-year study period (OR=3·57, CI^95^=3·44-3·71), and | quality |
|  | (1995-2006) |  | data and hospital |  |  |  | were also more likely to have ≥1 |  |
|  | (n=2,310,391) |  | discharge data |  |  |  | ED visit per year (OR=3·47, CI^95^=3·38-3·56) |  |
| Clark et al. | Patients with alcohol | Bipolar | ICD-9 diagnostic | Likelihood of ED or | Charlson/Deyo score, | Age, gender, race, | Having bipolar disorder was | 7 |
| 2013(61) | withdrawal admitted | disorder | codes for SMI | urgent care visit | illness severity score | payer source, smoker, | associated with a higher risk of an | Good |
|  | to medical ICU** | (n = 121) | recorded twice in | within a year of | based on admission | homeless, | ED or urgent care visit within one | quality |
|  | who did not die and  were not | Schizophrenia | the preceding  three years | hospital discharge | diagnosis and  comorbidities, depression, |  | year of hospital discharge  (HR=2·03, CI^95^=1·24-3·62, |  |
|  | rehospitalised in the  6-year study period | (n = 50) |  |  | anxiety |  | *p*=0·008), but schizophrenia was  not (HR=1·97, CI^95^=0·72-5·31, |  |
|  | (n=656) |  |  |  |  |  | *p*=0·124) |  |
| Hsieh et al. | Patients on the | Bipolar | Acute admission | Frequency of non- | No | Age, gender, | Patients with bipolar disorder had | 6 |
| 2012(21) | Taiwanese National | disorder | ICD-9 diagnostic | psychiatric ED visits |  | urbanisation level of | a significantly higher number of | Poor |
|  | Health Research | (n=4067) | codes | for those with bipolar |  | the residential area, | emergency care visits (*M*=2·50, | quality* |
|  | Institute (case-control |  |  | disorder and matched |  | monthly income | *SD*=3·35) compared to matched |  |
|  | design) |  |  | controls over 2-year |  |  | controls (*M*=1·51, *SD* =1·52) |  |
|  | (n=16,268) |  |  | study period |  |  | over the study period (*p*<0·0001) |  |
| Hunter et al. | Costliest 5% of | Bipolar | ICD-9 diagnostic | Number of ED visits | AHRQ comorbidity | Age, sex, | Patients with SMI had more ED | 8 |
| 2015(29) | Veterans Association | disorder, | codes and chronic | over the 12-month | measures | race/ethnicity, marital | visits over the study period | Good |
|  | patients | schizophrenia, | condition | study period |  | status, documented | (*M*=2·6) compared to patients | quality |
|  | (n=261,515) | other | indicators |  |  | homelessness during | with no mental health conditions |  |
|  |  | psychosis | established by |  |  | year of investigation, | (*M*=1·8) (*p*<0·001) |  |
|  |  | (n=33,119) | AHRQ |  |  | correlation within |  |  |
|  |  |  |  |  |  | facilities |  |  |
| Kheir et al. | Patients admitted for | Bipolar | ICD-9 diagnostic | Pre-operative ED | Cohort and controls | Also matched on age, | A significantly higher proportion | 7 |
| 2018(67) | total joint | disorder, | codes | visit | matched using Charlson | gender, BMI, joint, | of patients with SMI had a pre- | Good |
|  | arthroplasty | schizophrenia |  |  | Comorbidity Index | LOS, operative time, | operative ED visit (19/156, 12%) | quality |
|  | (n=579) | (n=156) |  |  |  | revision | compared to matched controls |  |
|  |  |  |  |  |  |  | (15/423, 3·5%) (*p*<0·001) |  |

| Kurdyak et al. 2017(31) | Patients with diabetes (n=1,131,375) | Schizophrenia (n=26,259) | Ontario Health Insurance Plan  records detailing | Number of ED visits for diabetic  complications; | Johns Hopkins ACG System, duration of  diabetes | Age, sex, rural residence,  neighbourhood | Patients with schizophrenia had an increased risk of an ED visit  for a diabetic complication | 8  Good quality |
| --- | --- | --- | --- | --- | --- | --- | --- | --- |
|  |  |  | three | number of ED visits |  | income, | (OR=1·34, CI^95^=1·28-1·41) and |  |
|  |  |  | schizophrenia- | for any non-mental |  | neighbourhood | an increased risk of an ED visit |  |
|  |  |  | related physician | health reason |  | material deprivation, | for any non-mental health reason |  |
|  |  |  | visits in 36  months, or a | excluding trauma  over the 2-year study |  | past year service use | excluding trauma (OR=1·72,  CI^95^=1·68-1·77) |  |
|  |  |  | hospitalisation for | period |  |  |  |  |
|  |  |  | schizophrenia |  |  |  |  |  |
| Lafeuille et al. | Patients with | Schizophrenia | ICD-9 diagnostic | Number of ED visits | Patients were matched 1:1 | Patients were matched | Compared to matched non- | 8 |
| 2014(32) | substance | (n=24,652) | codes – at least | over the 10-year | based on propensity | 1:1 based on | schizophrenic controls, patients | Good |
|  | dependence/abuse, |  | two primary or | study period | scores using the Charlson | propensity scores on | with schizophrenia had | quality |
|  | obesity, diabetes, |  | secondary |  | Comorbidity Index | age, gender, state, and | significantly higher rates of ED |  |
|  | metabolic syndrome, hyperlipidemia, |  | schizophrenia diagnoses |  |  | year of index admission | visits over the study period (IRR=2·08, CI^95^=1·95-2·23, |  |
|  | hypertension, |  | recorded during |  |  |  | *p*<0·005) |  |
|  | coronary artery |  | study period |  |  |  |  |  |
|  | disease, congestive |  |  |  |  |  |  |  |
|  | heart failure, HIV, |  |  |  |  |  |  |  |
|  | hepatitis C, or COPD |  |  |  |  |  |  |  |
|  | (n=49,304) |  |  |  |  |  |  |  |
| Minen et al. | Patients who visited | Bipolar | ICD-9 diagnostic | Number of ED visits | No | No | Patients with bipolar disorder had | 7 |
| 2014(37) | the ED with a | disorder | codes recorded in | over the 10-year |  |  | 1·85 times more emergency | Poor |
|  | primary diagnosis of | (n=NR) | the Partners | study period |  |  | department visits than patients | quality* |
|  | migraine |  | Research Patient |  |  |  | with other psychiatric disorders· |  |
|  | (n=2872) |  | Data Registry, |  |  |  |  |  |
|  |  |  | Massachusetts |  |  |  | No inferential analyses were |  |
|  |  |  |  |  |  |  | carried out on individual |  |
|  |  |  |  |  |  |  | psychiatric conditions, and no |  |
|  |  |  |  |  |  |  | comparison was made between |  |
|  |  |  |  |  |  |  | patients with bipolar disorder and |  |
|  |  |  |  |  |  |  | those without any psychiatric |  |
|  |  |  |  |  |  |  | diagnosis |  |

| Pugh et al. | Patients (veterans) | Bipolar | ICD-9 diagnostic | Number of ED | Epilepsy chronicity, | Age, sex, race, marital | Epilepsy patients with SMI were | 8 |
| --- | --- | --- | --- | --- | --- | --- | --- | --- |
| 2008(74) | with epilepsy | disorder, | codes | attendances over the | epilepsy severity, physical | status, patients with | more likely to attend an ED over | Good |
|  | (n=23,752) | schizophrenia, |  | 12-month study | comorbidities | service-connected | the study period compared to | quality |
|  |  | other |  | period |  | disability (therefore no | epilepsy patients without |  |
|  |  | psychoses  (n=1412) |  |  |  | co-payment required)  versus patients with a | psychiatric disease (OR=1·4,  CI^95^=1·2-1·7, *p*<0·01) |  |
|  |  |  |  |  |  | required co-payment |  |  |
| Shim et al. | Patients with diabetes | Schizophrenia | ICD-9 diagnostic | Total ED visits, ED | No | No | Diabetic patients with | 7 |
| 2014(75) | (n=340,786) | (n=23,913) | codes from claims | visits related to |  |  | schizophrenia had more total ED | Poor |
|  |  |  | data | diabetes, ED visits |  |  | visits per year (*M*=7·5, *SD*=8·1) | quality* |
|  |  |  |  | related to other |  |  | compared to diabetic patients |  |
|  |  |  |  | medical causes over |  |  | without schizophrenia (*M*=4·7, |  |
|  |  |  |  | the 2-year study |  |  | *SD*=4·4), *p*<0·01· |  |
|  |  |  |  | period |  |  |  |  |
|  |  |  |  |  |  |  | Diabetic patients with |  |
|  |  |  |  |  |  |  | schizophrenia had more ED visits |  |
|  |  |  |  |  |  |  | related to diabetes per year |  |
|  |  |  |  |  |  |  | (*M*=0·3, *SD*=0·7) compared to |  |
|  |  |  |  |  |  |  | diabetic patients without |  |
|  |  |  |  |  |  |  | schizophrenia (*M*=0·2, *SD*=0·5), |  |
|  |  |  |  |  |  |  | *p*<0·01· |  |
|  |  |  |  |  |  |  | Diabetic patients with |  |
|  |  |  |  |  |  |  | schizophrenia had more ED visits |  |
|  |  |  |  |  |  |  | related to other medical causes |  |
|  |  |  |  |  |  |  | per year (*M*=6·9, *SD*=7·6) |  |
|  |  |  |  |  |  |  | compared to diabetic patients |  |
|  |  |  |  |  |  |  | without schizophrenia (*M*=4·4, |  |
|  |  |  |  |  |  |  | *SD*=4·1), *p*<0·01· |  |

ACG = adjusted clinical groups; AHRQ = Agency for Healthcare Research and Quality; BMI = body mass index; CI = confidence interval; COPD = chronic obstructive pulmonary disorder; ED = emergency

department; HIV = human immunodeficiency virus; HR = hazard ratio; ICD = International Statistical Classification of Diseases and Related Health Problems; ICU = intensive care unit; IRR = incident rate ratio; LOS = length of hospital stay; M = mean; NOS = Newcastle-Ottawa Scale; NR = not reported; OR = odds ratio; SES = socioeconomic status; SD = standard deviation; SMI = severe mental illness

*If studies failed to adjust for physical comorbidities/illness severity in their analyses, they were deemed to be of poor quality regardless of ‘stars’ recorded using the NOS

**Table 5. The impact of comorbid severe mental illness on primary care use**

| **Authors** | **Population (n)** | **Comorbid SMI (n)** | **SMI assessment** | **Type of ED use** | **Control for**  **comorbidities/medical illness severity** | **Control for other variables** | **Main findings** | **NOS** |
| --- | --- | --- | --- | --- | --- | --- | --- | --- |
| Bresee et al. | All patients on the | Schizophrenia | ICD-9 and ICD- | Number of GP | Diagnosis of coronary | Age, sex, SES, urban | Patients with schizophrenia were | 8 |
| 2012(24) | Administrative | (n=28,755) | 10 diagnostic | encounters in the 2- | artery disease and/or | vs rural dwelling | more likely to have an encounter | Good |
|  | Database of Alberta Health and Wellness |  | codes from physician claims | year study period, ≥4 yearly GP encounters | diabetes |  | with their GP over the study period (OR=7·57, CI^95^=5·92- | quality |
|  | (1995-2006) |  | data and hospital |  |  |  | 9·69), and were more likely to |  |
|  | (n=2,310,391) |  | discharge data |  |  |  | have four or more GP encounters  per year (OR=3·60, CI^95^=3·49- |  |
|  |  |  |  |  |  |  | 3·71) |  |
| Copeland et al. | Patients with diabetes | Schizophrenia | ICD-9 diagnostic | Number of primary | No | No | Cluster analysis identified four | 5 |
| 2009(76) | (n=201,357) | (n=13,025) | codes from at | care visits over the 5- |  |  | clusters of primary care use over | Poor |
|  |  |  | least one | year study period |  |  | the study period: (1) increasing | quality |
|  |  |  | Veteran’s |  |  |  | use; (2) consistent use; (3) low to |  |
|  |  |  | Association |  |  |  | decreasing use; (4) high to |  |
|  |  |  | inpatient stay or |  |  |  | decreasing use· The proportion of |  |
|  |  |  | two outpatient |  |  |  | patients with diabetes only and |  |
|  |  |  | visits on different |  |  |  | diabetes + schizophrenia is |  |
|  |  |  | dates |  |  |  | presented below |  |
|  |  |  |  |  |  |  | Increasing: diabetes only 7%, |  |
|  |  |  |  |  |  |  | schizophrenia+diabetes 8% |  |
|  |  |  |  |  |  |  | Consistent: diabetes only 33%, |  |
|  |  |  |  |  |  |  | schizophrenia +diabetes 28% |  |
|  |  |  |  |  |  |  | Low decreasing: diabetes only |  |
|  |  |  |  |  |  |  | 52%, schizophrenia +diabetes |  |
|  |  |  |  |  |  |  | 54% |  |
|  |  |  |  |  |  |  | High decreasing: diabetes only |  |
|  |  |  |  |  |  |  | 8%, schizophrenia +diabetes 10% |  |
|  |  |  |  |  |  |  | The diabetes only group |  |
|  |  |  |  |  |  |  | dominates in the ‘consistent use’ |  |
|  |  |  |  |  |  |  | cluster· However, patients with |  |
|  |  |  |  |  |  |  | comorbid schizophrenia dominate |  |
|  |  |  |  |  |  |  | in the other three clusters· |  |
| Hunter et al. | Costliest 5% of | Bipolar | ICD-9 diagnostic | Number of primary | AHRQ comorbidity | Age, sex, | Patients with SMI had more | 8 |
| 2015(29) | Veterans Association | disorder, | codes and chronic | care appointments | measures | race/ethnicity, marital | primary care visits over the study | Good |
|  | patients | schizophrenia, | condition | over the 12-month |  | status, documented | period (*M*=6·1) compared to | quality |
|  | (n = 261,515) |  | indicators | study period |  | homelessness during |  |  |

|  | other  psychosis | established by  AHRQ |  | year of investigation,  correlation within | patients with no mental health  conditions (*M*=5·1) (*p*<0·001) |  |
| --- | --- | --- | --- | --- | --- | --- |
|  | (n=33,119) |  |  | facilities |  |  |
| Kontopantelis et | Patients in UK Schizophrenia, | Primary care | Number of primary | Hypertension, asthma, Patients matched on | Patients with SMI consulted more | 8 |
| al. 2015(77) | registered with affective | Read Codes | care visits over the | hypothyroidism, age, sex, and primary | in primary care than patients | Good |
|  | primary care practice psychoses |  | 12-year study period. | osteoarthritis, chronic care practice | without SMI across the 12-year | quality |
|  | (different data are (bipolar |  | This includes face-to- | kidney disease, coronary | study period· The difference |  |
|  | reported for each of disorder or |  | face, telephone, and | heart disease, epilepsy, | between these groups increased |  |
|  | the 12 fiscal years other |  | other (mail/email, | COPD, cancer, stroke, | after the introduction of QOF in |  |
|  | separately. We report unspecified |  | referrals, secondary | heart failure, rheumatoid | 2004· |  |
|  | sample sizes from the affective |  | care episode, other | arthritis, dementia, and |  |  |
|  | most recent year – psychosis), |  | administrative tasks) | psoriasis | Consultation rates for people with |  |
|  | 2011/12) other types of |  |  |  | SMI increased across all types |  |
|  | (n=5,069,748) psychosis |  |  |  | over time· For people with SMI, |  |
|  | (n=31,807) |  |  |  | the mean number of consultations |  |
|  |  |  |  |  | was 92% higher in 2011/12 |  |
|  |  |  |  |  | compared with 2000/01  (IRR=1·92, CI^95^=1·91-1·93)· For |  |
|  |  |  |  |  | matched control cases the |  |
|  |  |  |  |  | increase was smaller at 75% (IRR=1·75, CI^95^=1·74-1·75) |  |
| Lichstein et al. | Patients with two or Schizophrenia | ICD-9 diagnostic | Use of medical | Total number of chronic Age, gender, race, | Patients with schizophrenia had | 8 |
| 2014(78) | more chronic (n=10,166) | codes | homes over the 3- | conditions (between 2 and ethnicity | an 8·2% lower probability of | Good |
|  | conditions: major |  | year study period. | 8), included all diagnosis | having a medical home visit over | quality |
|  | depressive disorder, |  | Medical homes | indicators for major | the study period compared to |  |
|  | schizophrenia, |  | provide a model of | depressive disorder, | patients without schizophrenia or |  |
|  | hypertension, |  | primary care that is | diabetes, asthma, | depression (*p*<0·01)· |  |
|  | diabetes, |  | patient-centred, | hypertension, |  |  |
|  | hyperlipidaemia, |  | comprehensive, team- | hyperlipidaemia, seizure | Patients with schizophrenia had |  |
|  | seizure disorder, |  | based, co-ordinated, | disorder, and COPD. | one fewer (-1·02, *SE*=0·10) |  |
|  | asthma, COPD |  | accessible and |  | medical home visits over the |  |
|  | (n=105,542) |  | focused on quality |  | study period compared to patients |  |
|  |  |  | and safety. |  | without schizophrenia or |  |
|  |  |  |  |  | depression (*p*<0·01) |  |

| Pugh et al. | Patients (veterans) | Bipolar | ICD-9 diagnostic | High primary care | Epilepsy chronicity, | Age, sex, race, marital Epilepsy patients with SMI were | 8 |
| --- | --- | --- | --- | --- | --- | --- | --- |
| 2008(74) | with epilepsy | disorder, | codes | use (top 20% of | epilepsy severity, physical | status, patients with no more likely to be high primary | Good |
|  | (n=23,752) | schizophrenia, |  | patients with high | comorbidities | service-connected care users over the study period | quality |
|  |  | other |  | utilisation) over the |  | disability (therefore no than epilepsy patients without |  |
|  |  | psychoses  (n=1412) |  | 12-month study  period |  | co-payment required) psychiatric disease (OR=0·9,  versus patients with a CI^95^=0·7-1·1) |  |
|  |  |  |  |  |  | required co-payment |  |

AHRQ = Agency for Healthcare Research and Quality; CI = confidence interval; COPD = chronic obstructive pulmonary disorder; GP = general practitioner; ICD = International Statistical Classification of Diseases

and Related Health Problems; IRR = incident rate ratio; NOS = Newcastle-Ottawa Scale; OR = odds ratio; QOF = Quality Outcomes Framework; SES = socioeconomic status; SMI = severe mental illness; UK = United Kingdom

*If studies failed to adjust for physical comorbidities/illness severity in their analyses, they were deemed to be of poor quality regardless of ‘stars’ recorded using the NOS.

Figure 1 PRISMA flow diagram	[Click here to access/download;Figure;Figure 1_PRISMA flow diagram.docx](https://www.editorialmanager.com/pmedicine/download.aspx?id=651042&guid=c707d6a4-04f2-4a01-b4ea-1c4f143132d1&scheme=1)

124 full-text articles excluded:

- 25 conference abstracts
- 4 full-text unavailable
- 1 incorrect definition of PD
- 16 incorrect definition of SMI
- 1 non-English language article
- 2 patients <16 years
- 1 study protocol
- 2 systematic reviews
- 39 the impact of SMI on health service utilisation was not assessed
- 16 wrong outcome
- 16 wrong patient group
- 1 reported incorrect results

196 full-text articles assessed for eligibility

1 provided through author correspondence

1 identified through secondary search

3321 excluded because of unsuitability

3515 identified for title and abstract screening

8 identified through secondary search

1113 excluded because of duplication

4620 references retrieved for review of title or abstract identified by database search

72 studies included after full- text screening

61 studies examined inpatient service use (hospital admissions, length of hospital stay, readmission rates), emergency department use, and/or primary care service use

11 articles excluded from present study:

- 4 examined the impact of personality disorder
- 3 examined medical costs only
- 4 examined ‘other’ health services only

**Figure 1.** PRISMA Flowchart of study selection

Figure 2 forest plot	[Click here to access/download;Figure;Figure 2_IP admission forest plot.docx](https://www.editorialmanager.com/pmedicine/download.aspx?id=651043&guid=3cb7d7b8-9808-43d6-b125-aec1af8025f6&scheme=1)

**Figure 2.** The impact of severe mental illness on inpatient hospital admissions. Several studies included analyses for different samples and included different outcomes. Therefore, several effect estimates have been entered from the same studies.

Figure 3 forest plot	[Click here to access/download;Figure;Figure 3_LOS forest plot.docx](https://www.editorialmanager.com/pmedicine/download.aspx?id=651044&guid=a10f0036-a32a-479d-b79d-9bee761b823b&scheme=1)

**Figure 3.** The impact of severe mental illness on length of hospital stay (days). Standardised mean difference values are presented. Two means were entered into the meta-analysis from Falsgraf et al. [47] as data were presented for patients with bipolar disorder and schizophrenia separately.

Figure 4 forest plot	[Click here to access/download;Figure;Figure 4_30day forest plot.docx](https://www.editorialmanager.com/pmedicine/download.aspx?id=651046&guid=d3980427-79c2-495b-8bee-948f02a258e8&scheme=1)

**Figure 4.** The impact of severe mental illness on 30-day hospital readmission rate. Several studies included analyses for different samples and included different outcomes. Therefore, several effect estimates have been entered from the same studies.

Figure 5 forest plot	[Click here to access/download;Figure;Figure 5_ED forest plot.docx](https://www.editorialmanager.com/pmedicine/download.aspx?id=651047&guid=158dc9af-a2ca-4d9a-b1cd-3360f342d3c4&scheme=1)

**Figure 5.** The impact of severe mental illness on use of emergency departments. Two studies performed analyses on different outcomes. Therefore, two effect estimates have been entered for these studies.

Appendix 1

[Click here to access/download](https://www.editorialmanager.com/pmedicine/download.aspx?id=651024&guid=8ebebfb3-9b91-4464-a82a-f5dda74449f5&scheme=1)

[Supporting Information Appendix 1_Search Strategy.docx](https://www.editorialmanager.com/pmedicine/download.aspx?id=651024&guid=8ebebfb3-9b91-4464-a82a-f5dda74449f5&scheme=1)

Appendix 2

[Click here to access/download](https://www.editorialmanager.com/pmedicine/download.aspx?id=651025&guid=e74d41ae-9575-4c18-9020-fd8efddebf58&scheme=1)

[Supporting Information Appendix 2_Excluded studies.docx](https://www.editorialmanager.com/pmedicine/download.aspx?id=651025&guid=e74d41ae-9575-4c18-9020-fd8efddebf58&scheme=1)

Appendix 3

[Click here to access/download](https://www.editorialmanager.com/pmedicine/download.aspx?id=651026&guid=4edbe255-8802-4616-a68c-81ffceed0186&scheme=1)

[Supporting Information](https://www.editorialmanager.com/pmedicine/download.aspx?id=651026&guid=4edbe255-8802-4616-a68c-81ffceed0186&scheme=1)

[Appendix 3_SubgroupAnalysis_Tables.docx](https://www.editorialmanager.com/pmedicine/download.aspx?id=651026&guid=4edbe255-8802-4616-a68c-81ffceed0186&scheme=1)

Appendix 4

[Click here to access/download](https://www.editorialmanager.com/pmedicine/download.aspx?id=651027&guid=f76a4b7b-9c13-4598-a0af-f1a6cca50a58&scheme=1)

[Supporting Information Appendix 4_Funnel plots_Draft2.docx](https://www.editorialmanager.com/pmedicine/download.aspx?id=651027&guid=f76a4b7b-9c13-4598-a0af-f1a6cca50a58&scheme=1)
