# Supplementary material for: Severe mental illness and health service utilisation for nonpsychiatric medical disorders: A systematic review and meta-analysis
Source: PLoS Med. 2020 Sep 14;17(9):e1003284. doi: 10.1371/journal.pmed.1003284 (PMC7489517; doi:10.1371/journal.pmed.1003284)
Supplement: Attachment — Submitted filename: Response to reviewer comments_3.docx [file pmed.1003284.s008.docx]

At this stage, we need to make sure that certain production issues are addressed in order to ensure a smooth handover to our production team, should the manuscript be formally accepted.

Can you please address the following issues as soon as possible? Your paper has been sent back to enable you to make these changes.

1. Please indicate the corresponding author on the title page with an asterisk (*) and include the corresponding author's email address. Only an asterisk and the email address itself are required.

**The corresponding author has been indicated using an asterisk and email address provided on title page.**

2. Please remove author degree titles from the byline of your manuscript file.

**There are no author degree titles in the byline of the manuscript.**

3. We have noted that you have the following present in your manuscript: List of keywords/ Financial Disclosure/ Author Contributions

We have removed these section from your manuscript file as this information is automatically pulled from the submission form. Please make sure that the information in the submission form is correct.

**The List of Keywords and Author Contributions have been deleted from the manuscript, as well as the Role of the Funding Source from the title page.**

4. PLOS does not publish Appendices. Please incorporate them into the main manuscript, or upload each of your Appendices as a Supporting Information file in the form "S1 Text", “S2 Text” etc, and refer to it throughout the manuscript as such. For more information, please see <https://eur03.safelinks.protection.outlook.com/?url=http%3A%2F%2Fjournals.plos.org%2Fplosbiology%2Fs%2Fsupporting-information&amp;data=01%7C01%7Camy.ronaldson%40kcl.ac.uk%7C5bed45201e684307d5a908d828cdbee3%7C8370cf1416f34c16b83c724071654356%7C0&amp;sdata=C1KmVnaHxceG3ydmgIRVZWPsDRHd%2Fjjq%2BZfCsV47GMQ%3D&amp;reserved=0>. <https://eur03.safelinks.protection.outlook.com/?url=https%3A%2F%2Fjournals.plos.org%2Fplosmedicine%2Fs%2Flatex&amp;data=01%7C01%7Camy.ronaldson%40kcl.ac.uk%7C5bed45201e684307d5a908d828cdbee3%7C8370cf1416f34c16b83c724071654356%7C0&amp;sdata=i2LWgpQOIQkcGYYNOu%2Fas61ei6Wx29cRswnflSzG5a4%3D&amp;reserved=0>

**I have uploaded the Appendices as separate Supporting Information files and have referred to them throughout the document as advised (i.e. S1 Appendix, S2 Appendix, etc).**

5. Please ensure that all Supporting Information files are clearly labled in the file inventory. i.e. S1_Fig.tif, S1_Table.xlsx, S1_Text.doc, S1_Data.xlsx, S1_PRISMA_Checklist.docx, etc.

Each file type should be numbered starting at 1. Please also make sure that the file description matches the file name (e.g. S1_Fig.tif is listed as S1 Fig).

**We have ensured that all the Supporting Information files are clearly labelled in the file inventory and have included a Supporting Information legend after the Reference section in the main manuscript.**

6. Please make sure all references are cited in ascending numerical order in the text, e.g. on page 43, reference 63 is cited before reference 62. Reference 64 is cited on page 36 and your reference 65 is cited on page 38. Reference 83 is cited on page 44. Reference 84 is cited on page 46. Reference 73 is not cited.

**I am not sure how these inconsistencies occurred as I used a reference manager software throughout (Zotero). I have now reinserted all the references and believe any issues have been resolved. Please note that in the Tables at the end of the manuscript, the references will not necessarily be in numerical order as they have already been cited in the main text. There is also considerable overlap in terms of cited studies across the sections of the review meaning that the references will not be in order in the Tables.**

7. Please note that your figures do not currently comply with our publication requirements. For further instructions to assist with your Figure revisions, please refer to our Figure Guidelines at: <https://eur03.safelinks.protection.outlook.com/?url=http%3A%2F%2Fjournals.plos.org%2Fplosmedicine%2Fs%2Ffigures&amp;data=01%7C01%7Camy.ronaldson%40kcl.ac.uk%7C5bed45201e684307d5a908d828cdbee3%7C8370cf1416f34c16b83c724071654356%7C0&amp;sdata=tLzgjAfW63Y3kgwJxHnYDi5FNTrU8qsQ7q2qcxM4ics%3D&amp;reserved=0>.

If you need assistance, please contact us directly at [graphics@jjeditorial.com](mailto:graphics@jjeditorial.com) so we can further help with this matter. Please send any relevant source files and tell us what application(s) and what operating system you are using.

- Figures 1-5 have been submitted in Indexed Color, a color space that we do not accept for Figure files. Please provide us with Figures in RGB (millions of colors), 8 bits/channel, saved as a high-resolution TIFF or EPS.

- Please crop Figures 1-5 to remove the whitespace around the figures themselves. PLOS recommends a 2-point whitespace border, but more will cause the figure to be incorrectly placed in the published PDF.

- Figure 1 exceeds the dimension limits for figures. Figures must be less than 19.05 cm in width and less than 22.225 cm in height (excluding white space around your artwork) and between 300-600 pixels/inch in resolution. Please make the necessary adjustments and re-upload your figure(s).

-In Photoshop: Image→Image Size. Make sure the Resample Image checkbox is NOT checked. If the Width is over 19.05 cm, type 19.05 in the Width box (19.05 cm is our maximum allowable width for figures).

-In GIMP: Image→Scale Image. Set the units of measurement, in the pull down menu next to Height, to millimeters. If the Width is over 190.5 mm, type 190.5 in the Width box (19.05 cm is our maximum allowable width for figures) and hit Tab. The new Height of the figure will appear, scaled proportionately to the change in Width.

- Figures 2, 3, 4, and 5 look like they were originally at a lower resolution and will appear blurry when published. This error cannot be fixed by simply increasing the resolution of your figures with image editing software. Instead, please carefully review the PLOS Figure Guidelines and provide figure files that were originally created at a minimum of 300 pixels/inch (ppi) and that are sized between 6.68-19.05 cm wide and no more than 22.225 cm tall (excluding any white space around your artwork). Additionally, please ensure your figures are in RGB colour mode.

**I have now liaised with the Graphics Department at PLOS Medicine who very kindly helped me to format the Figures correctly.**
